# Regeneration of severely damaged lungs using an interventional cross-circulation platform

**DOI:** 10.1038/s41467-019-09908-1

**Published:** 2019-05-07

**Authors:** Brandon A. Guenthart, John D. O’Neill, Jinho Kim, Dawn Queen, Scott Chicotka, Kenmond Fung, Michael Simpson, Rachel Donocoff, Michael Salna, Charles C. Marboe, Katherine Cunningham, Susan P. Halligan, Holly M. Wobma, Ahmed E. Hozain, Alexander Romanov, Gordana Vunjak-Novakovic, Matthew Bacchetta

**Affiliations:** 1 Department of Biomedical Engineering, Columbia University Medical Center, Columbia University, New York, NY 10032 USA; 2 Department of Surgery, Columbia University Medical Center, Columbia University, New York, NY 10032 USA; 3 Department of Biomedical Engineering, Stevens Institute of Technology, Hoboken, NJ 07030 USA; 4 Department of Clinical Perfusion, Columbia University Medical Center, Columbia University, New York, NY 1003 USA; 5 Institute of Comparative Medicine, Columbia University Medical Center, Columbia University, New York, NY 10032 USA; 6 Department of Pathology and Cell Biology, Columbia University Medical Center, Columbia University, New York, NY 10032 USA; 7 Department of Medicine, Columbia University Medical Center, Columbia University, New York, NY 10032 USA; 8 Department of Thoracic and Cardiovascular Surgery, Vanderbilt University, Nashville, TN 37232 USA

**Keywords:** Regeneration, Respiratory tract diseases, Organ transplantation, Biomedical engineering

## Abstract

The number of available donor organs limits lung transplantation, the only lifesaving therapy for the increasing population of patients with end-stage lung disease. A prevalent etiology of injury that renders lungs unacceptable for transplantation is gastric aspiration, a deleterious insult to the pulmonary epithelium. Currently, severely damaged donor lungs cannot be salvaged with existing devices or methods. Here we report the regeneration of severely damaged lungs repaired to meet transplantation criteria by utilizing an interventional cross-circulation platform in a clinically relevant swine model of gastric aspiration injury. Enabled by cross-circulation with a living swine, prolonged extracorporeal support of damaged lungs results in significant improvements in lung function, cellular regeneration, and the development of diagnostic tools for non-invasive organ evaluation and repair. We therefore propose that the use of an interventional cross-circulation platform could enable recovery of otherwise unsalvageable lungs and thus expand the donor organ pool.

## Abstract (web summary)

Gastric aspiration severely injures donor lungs, frequently making them unacceptable for transplantation. Here the authors show that an interventional cross-circulation platform enables the regeneration of severely damaged lungs in a swine model of gastric aspiration injury.

## Introduction

Organ transplantation has overcome significant technical, immunological, and ethical hurdles to become a lifesaving treatment^1^. The full potential of organ transplantation remains constrained by the global shortage of organs—a problem that has been described as “the greatest crisis facing biomedicine today”^2^. Notably, up to 80% of donated lungs are not utilized^3^, often as a result of injury at the time of death (e.g., trauma, pulmonary contusion, aspiration, ventilator-associated lung injury). Major efforts to expand the donor pool are underway^4–6^, including ex vivo lung perfusion (EVLP), which is under clinical investigation as a means to evaluate and improve marginal quality donor lungs^7^. With the use of hyperosmotic and blood-based perfusates, EVLP has been shown to decrease pulmonary edema and enable ~6 h of normothermic perfusion prior to transplantation^8,9^. EVLP is also used as an investigational platform for the delivery of therapeutic agents including antibiotics^10^, pulmonary surfactant^11,12^, immunomodulatory viral vectors^13,14^, and mesenchymal stem cells^15,16^.

Despite the clinical use of EVLP since its introduction in 2012 and expansion of criteria for donor lungs, the annual number of lung transplants in the United States has not dramatically increased^17^. Consequently, there is a compelling need to extend organ recovery capabilities beyond the limited category of marginal lungs to severely damaged lungs, such as those injured by aspiration of gastric contents (hydrochloric acid, bile acids, gastric enzymes, and food particulates^18,19^). Gastric aspiration results in damage to the pulmonary epithelium, leading to pneumonitis and respiratory insufficiency^18^. Previous attempts to recover lungs injured by gastric aspiration using EVLP have been unsuccessful^20^, having resulted in increased edema^21^ and failure to reduce levels of inflammatory cytokines^22^ or improve tissue morphology^23^ (Supplementary Table 1–3).

We previously reported the development of an extracorporeal organ support platform that utilized cross-circulation to radically extend the duration of time that lungs can be maintained outside the body (normothermic perfusion time 37.7 ± 1.8 h; total extracorporeal preservation time 56.2 ± 0.1 h)^24^. Following these prolonged extracorporeal preservation times, lungs met all transplantation criteria and showed maintenance of pulmonary vasoregulation, microvascular and bronchial epithelial tight junctions, alveolar type II pneumocyte surfactant recycling, airway submucosal gland secretory activity, and a coordinated mucociliary escalator. Based on these results, we hypothesized that normothermic perfusion via cross-circulation could enable therapeutic interventions leading to functional recovery and regeneration of severely damaged lungs.

In this study, we use an established clinically relevant large animal model of gastric aspiration to recapitulate severe injury that currently renders a large number of donated lungs unsalvageable for clinical transplantation. We investigate the ability of the cross-circulation platform to: (i) provide prolonged normothermic extracorporeal support of severely damaged lungs outside the body, and (ii) enable continuous assessment of lung regeneration at the molecular, cellular, and tissue levels. Severely damaged lungs are subjected to multiple therapeutic interventions—bronchoalveolar lavage, surfactant replacement, and alveolar recruitment—on an interventional cross-circulation platform that provided physiologic systemic regulation. We also apply non-invasive diagnostics (exosomes, surface thermography) in extracorporeal lungs on cross-circulation support, and establish benchmarks of lung repair and regeneration and corresponding scope of analyses (Supplementary Table 4).

## Results

### Experimental design

**Fig. 1:**
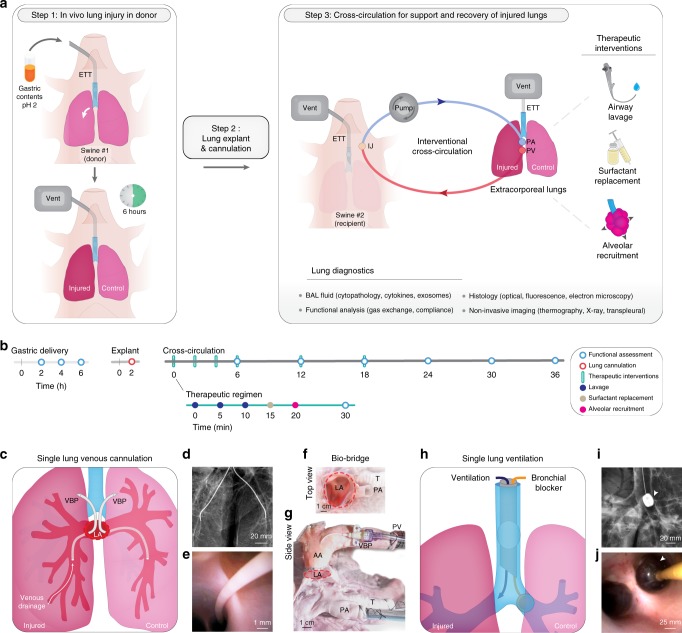
Experimental overview and technique for single lung functional assessment. **a** Setup of interventional cross-circulation procedures. Lungs were subjected to injury in vivo by aspiration via bronchoscopic delivery of standardized gastric contents, explanted after 6 h, cannulated, and recovered by cross-circulation. ETT endotracheal tube, IJ internal jugular vein, PA pulmonary artery, PV pulmonary vein. Detailed descriptions and photographs of in vivo injury, lung cannulation procedures, and cross-circulation equipment and setup are available in Supplementary Fig. 1–3 and Supplementary Movie 1. **b** Timeline of gastric aspiration injury and lung recovery by therapeutic interventions and cross-circulation. Single lung venous cannulation technique: **c** Schematic of venous cannulae placed in the left and right pulmonary veins and exiting out of the left atrial (LA) cuff to venous blood ports (VBP), allowing for blood–gas analysis of venous drainage from injured and control lungs. **d** X-ray confirming position of cannulae within the left and right pulmonary veins. **e** Fiber optic image confirming cannulation of a pulmonary vein. Management of pulmonary venous drainage. **f** LA cuff after removal of the heart, shown in relation to the pulmonary artery (PA) and trachea (T). **g** Lungs fully cannulated prior to cross-circulation. Aortic arch (AA) serving as a bio-bridge between the LA cuff and the pulmonary venous (PV) drainage cannula. Placed and secured through the bio-bridge are selective VBPs (only one VBP is visualized on this side view). Single lung ventilation technique: **h** Schematic of bronchial blocker placed in one main stem bronchus, enabling isolated ventilation and data acquisition from a single lung. **i** X-ray confirming position of bronchial blocker within main stem bronchus with balloon inflated (arrow). **j** Bronchoscopy showing balloon inflated (arrow) and secured in main stem bronchus of unventilated lung

**Fig. 2:**
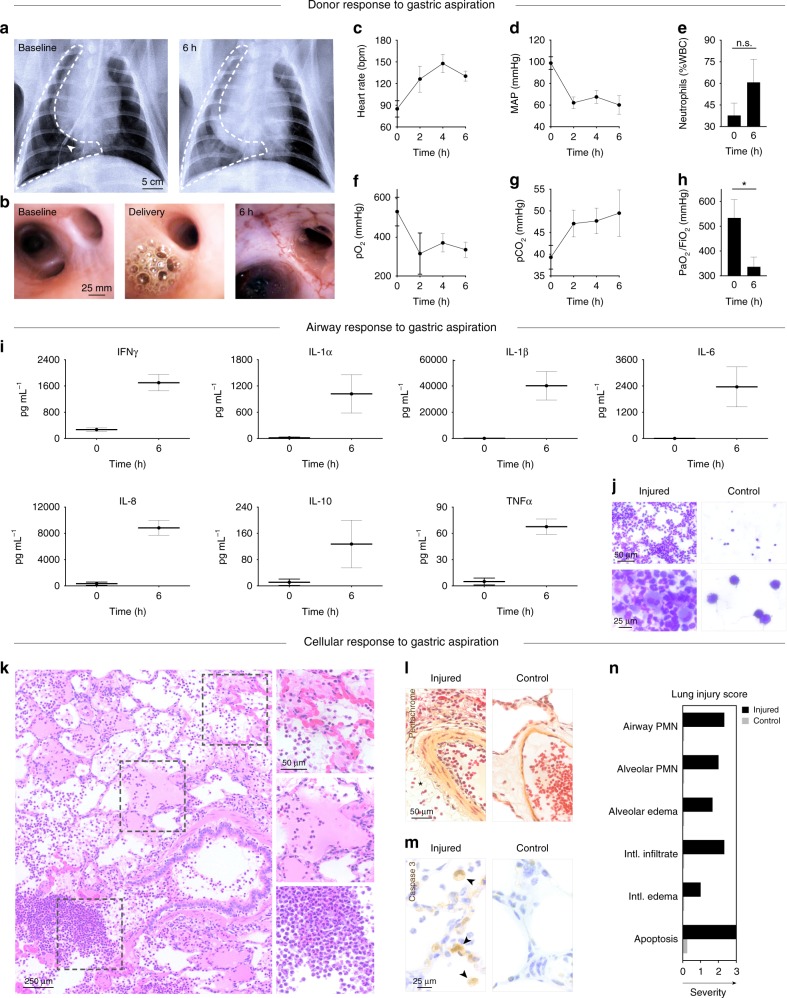
In vivo response to gastric aspiration injury. **a** Donor chest X-ray at baseline and 6 h after gastric aspiration. Arrow indicates tip of bronchoscope within the injured lung. **b** Bronchoscopic analysis of pulmonary airways at baseline, at the time of aspiration, and 6 h after gastric aspiration. Systemic response of donor following gastric aspiration: **c** heart rate, **d** mean arterial pressure (MAP), and **e** neutrophils (% white blood cells (WBC)), Student’s *t*-test for 0 h versus 6 h, not significant (n.s.). Lung function of donor following aspiration: **f** oxygenation (pO_2_), **g** ventilation (pCO_2_), and (**h**) PaO_2_/FiO_2_ ratio, Student’s *t*-test for 0 h versus 6 h, *p *<* 0.05. **i** Inflammatory response measured in donor bronchoalveolar lavage (BAL) fluid at baseline and 6 h after gastric aspiration injury; for all graphs shown, Student’s *t*-test for 0 h versus 6 h, *p* *<* 0.001. **j** Kwik-Diff staining of BAL fluid smears from injured and control lungs 6 h after gastric aspiration. **k** H&E staining of injured lung 6 h after gastric aspiration, with high magnification insets demonstrating regions of alveolar hemorrhage, alveolar edema and cellular exudate, and exudate of leukocytes into alveoli. **l** Pentachrome staining 6 h after gastric aspiration. Perivascular edema and infiltration noted in the injured lung (star). **m** Caspase 3 immunostaining, a marker of early apoptosis (arrows), 6 h after gastric aspiration. **n** Lung injury score obtained at 6 h after gastric aspiration. PMN polymorphonuclear leukocytes. All graphs represent data from *n* *=* 8 lungs. All values represent mean ± standard deviation

To induce lung injury, gastric contents (2 mL kg^−1^; pH 2) were delivered into a single lung of an anesthetized swine via flexible video bronchoscopy, and an acute injury was allowed to develop for 6 h (Fig. 1a; Supplementary Movie 2). Progression of lung injury was assessed every 2 h by radiography, bronchoscopy, bronchoalveolar lavage fluid analysis, and serologic and hemogas analyses (Fig. 2, Supplementary Table 5). Upon explant, extensive lung consolidation, cellular derangement, peribronchial edema, and regions of alveolar hemorrhage, edema, and pervasive infiltration of leukocytes were observed (Supplementary Fig. 1). Lungs were cannulated, and pulmonary venous drainage was managed with cannulas placed through a vascular bio-bridge to enable single lung functional assessment (i.e., gas exchange in injured and control lungs; Fig. 1c–g; Supplementary Fig. 2a–h). An endotracheal tube was inserted and secured in the extracorporeal lungs to enable continuous ventilation throughout the duration of the study. Periodic use of bronchial blockers enabled single lung assessment of compliance of injured and control lungs (Fig. 1h–j; Supplementary Fig. 2i–m). Following cannulation of the internal jugular veins of the recipient, lungs were connected to the extracorporeal circuit and cross-circulation was established. All cross-circulation procedures were conducted in an operating room and utilized clinical equipment and supporting devices (Supplementary Fig. 3).

### Validation of in vivo lung injury after gastric aspiration

Video bronchoscopy was used to confirm unilateral delivery of gastric contents into lungs (Fig. 2b; Supplementary Movie 2). Six hours after in vivo delivery of gastric contents, chest X-rays showed marked unilateral lung injury characterized by increased radiopacity, basilar atelectasis, and areas of consolidation with air bronchograms (Fig. 2a). Bronchoscopy revealed severe unilateral airway inflammation, edema, and mucus secretions (Fig. 2b) resulting from the insult of gastric contents. Lung donors experienced hemodynamic instability (increased heart rate, decreased MAP; Fig. 2c, d), significantly decreased gas exchange capacity (Fig. 2f–h), and developed a severe inflammatory response (significant elevation in levels of IFNγ, IL-1α, IL-1β, IL-6, IL-8, TNFα in the bronchoalveolar lavage (BAL) fluid, *p* < 0.001; Fig. 2i), consistent with previously described effects of large volume aspiration^25^. Significantly more inflammatory cells, including lymphocytes, polymorphonuclear cells, and mast cells, were observed in BAL fluid from injured lungs compared to BAL fluid from control lungs (Fig. 2j**;** Supplementary Fig. 1j).

Histologic and immunohistochemical evaluation revealed dramatic inflammatory response, cellular derangement, and peribronchial edema, with localized regions of alveolar hemorrhage, alveolar edema, pervasive infiltration of leukocytes (Fig. 2k; Supplementary Fig. 1e–h), perivascular edema, vascular intimal thickening, and extravasation of inflammatory cells (Fig. 2l) in injured lungs. Immunostaining for caspase 3, a marker of early apoptosis, showed numerous apoptotic cells throughout injured lungs 6 h after gastric aspiration, while apoptotic cells were very rarely observed in control lungs (Fig. 2m). Pathologic assessments were performed in a randomized and blinded fashion according to a previously established protocol^24,26,27^ (Supplementary Table 6) to obtain lung injury scores of injured and control lungs at baseline and 6 h after gastric aspiration. Injured lungs received significantly higher injury scores than control lungs across all injury categories (Fig. 2n; Supplementary Fig. 1k).

### Therapeutic intervention in lungs during cross-circulation

**Fig. 3:**
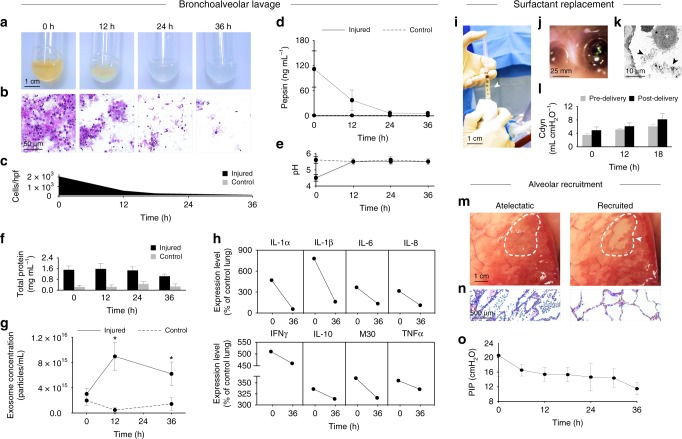
Therapeutic interventions in injured lungs during cross-circulation. **a** Gross imaging of BAL fluid samples collected from injured lungs over 36 h of cross-circulation. **b** Periodic acid-Schiff staining of BAL fluid smears obtained from injured lungs. **c** Quantification of cells in BAL fluid samples. **d** Pepsin concentration, **e** pH, **f** total protein, and **g** exosome concentration in BAL fluid samples over 36 h of cross-circulation, Student’s *t*-test for Injured versus Control, *p *<* 0.001. **h** Mean cytokine expression levels in injured lungs, normalized as a percent of mean levels in control lungs, measured in BAL fluid between initiation of cross-circulation (0 h) and conclusion of cross-circulation (36 h). Cytokine concentrations (mean ± standard deviation) throughout 36 h of cross-circulation in Supplementary Fig. 5c. Surfactant replacement: **i** Bronchoscopic delivery of therapeutic surfactant into injured lung (arrow). **j** Bronchoscopic image demonstrating therapeutic surfactant delivered into airways within the injured lung. **k** Transmission electron micrograph (TEM) demonstrating abundant surfactant (arrows) within alveoli following delivery. Alveolar macrophage (star) and adjacent lymphocyte observed within the same alveoli. **l** Dynamic compliance (Cdyn) of injured lungs pre- and post-delivery of therapeutic surfactant over the first 18 h of cross-circulation. **m** Alveolar recruitment: before recruitment (atelectatic) and after recruitment (recruited). Arrow and dotted line indicates a region of recruitment. **n** Silver reticulin staining of collapsed consolidated lung, and recruited lung with open alveoli. **o** Peak inspiratory pressure (PIP) of injured lungs throughout 36 h of cross-circulation. All graphs represent data from *n* *=* 8 lungs. All values represent mean ± standard deviation

Bronchoalveolar lavage (performed with a flexible video bronchoscope, Supplementary Movie 4), surfactant replacement, and alveolar recruitment maneuvers were conducted according to a specified sequence of interventions and functional assessments (Fig. 1b). Analysis of BAL fluid from injured lungs demonstrated significant decreases in turbidity, sediment (gastric contents, airway secretions, and debris), proteinaceous contents, and cell counts (0 h, 1816 ± 244 cells; 36 h, 120 ± 31 cells; *p* < 0.001) over 36 h of cross-circulation (Fig. 3a–c). The concentration of pepsin in BAL fluid from injured lungs also decreased significantly over 36 h of cross-circulation (0 h, 111.9 ± 43.2 ng mL^−1^; 36 h, 5.8 ± 3.9 ng mL^−1^; *p* < 0.001), while the pH (0 h, 4.5 ± 0.2) normalized to the pH of BAL fluid from control lungs (5.5 ± 0.1) by 12 h of cross-circulation (Fig. 3d, e). Total protein in BAL fluid from injured lungs gradually decreased over 36 h of cross-circulation (0 h, 1.51 ± 0.29 mg mL^−1^; 36 h, 1.04 ± 0.16 mg mL^−1^) but remained consistently elevated compared to BAL fluid from control lungs (0 h, 0.25 ± 0.13 mg mL^−1^; 36 h, 0.28 ± 0.18 mg mL^−1^, Fig. 3f).

Interestingly, the concentration of exosomes in BAL fluid from injured lungs was significantly elevated at 12 h of cross-circulation (injured, 8.96 × 10^15^ ± 2.24 × 10^15^ particles mL^−1^; control, 4.88 × 10^14^ ± 3.90 × 10^14^ particles mL^−1^; *p* < 0.01) but trended downward between 12 and 36 h of cross-circulation (Fig. 3g). Correlation between injury/inflammation and exosome concentration has been previously reported in other pathologic conditions^28,29^, and these results suggest that the concentration of exosomes in BAL fluid may serve as a diagnostic marker of extracorporeal lung injury and recovery. Exosomes in BAL fluids from injured and control lungs had comparable mean diameters and size distributions (Supplementary Fig. 4d–f). In parallel, the effect of interventional cross-circulation on the inflammatory response in injured lungs was analyzed by quantification of cytokines in BAL fluid. GM-CSF, IFNγ, IL-1α, IL-1β, IL-2, IL-4, IL-6, IL-8, IL-12, IL-18, M30, and TNFα all decreased over 36 h of cross-circulation (Fig. 3h, Supplementary Table 9, Supplementary Fig. 4c).

Following airway lavage, exogenous surfactant was delivered via video bronchoscopy into each lobe of injured lungs (Fig. 3i, j; Supplementary Fig. 8g), and was observed advancing distally through the airway tree during positive pressure ventilation, consistent with the transport and deposition of liquid plugs through small airways^30,31^ (Supplementary Movie 5). Transmission electron microscopy confirmed the presence of abundant surfactant in distal airspaces (Fig. 3k; Supplementary Fig. 8h). Dynamic compliance of injured lungs consistently increased after surfactant delivery and throughout the duration of interventional cross-circulation (Fig. 3l). Following surfactant replacement, alveolar recruitment maneuvers were performed by adjusting ventilator settings to recruit additional lung volumes^32,33^. Gross imaging of the lung surface (Fig. 3m, Supplementary Movie 6) and histologic analysis (Fig. 3n) showed that atelectatic regions throughout the injured lung were successfully recruited over 36 h of interventional cross-circulation. Peak inspiratory pressures (PIP) during standard ventilation gradually decreased from 20.6 ± 5.8 cmH_2_O to 11.5 ± 1.7 cmH_2_O over 36 h of cross-circulation (Fig. 3o), consistent with lung recovery and recruitment of additional lung volumes.

### Functional assessment of lungs during cross-circulation

**Fig. 4:**
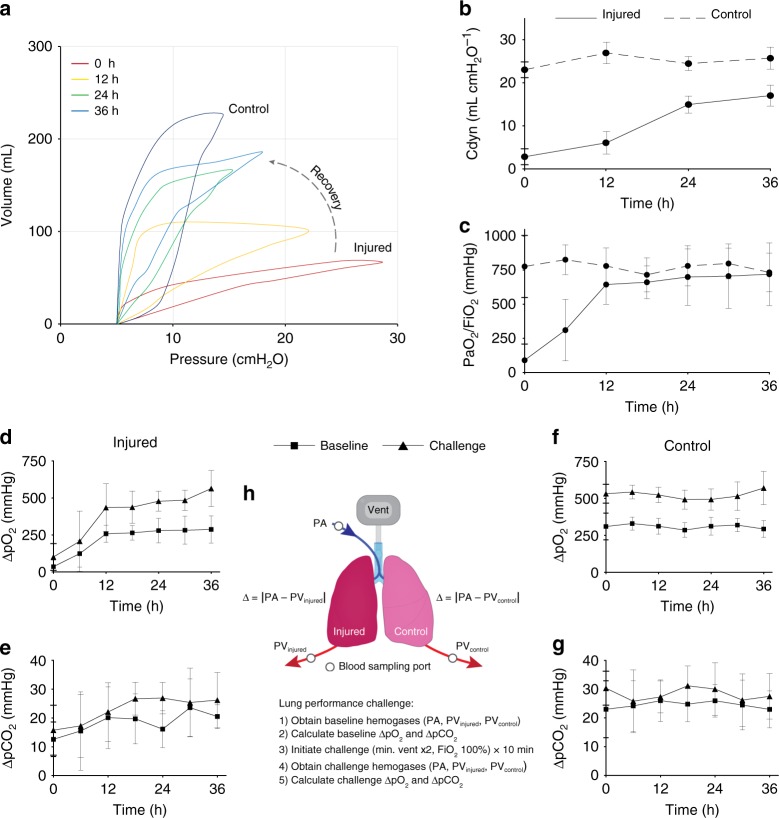
Extracorporeal lung performance and functional recovery. **a** Pressure-volume (PV) loops: injured lungs demonstrating initial derangement in PV loops and recovery towards normal hysteresis observed in control lungs. **b** Dynamic compliance (Cdyn) over 36 h of cross-circulation. **c** PaO_2_/FiO_2_ over 36 h of cross-circulation. Responses in ΔpO_2_ and ΔpCO_2_ to 10 min lung performance challenges throughout 36 h of cross-circulation in **d, e** injured and **f, g** control lungs. **h** Schematic depicts arterial and single lung venous blood sample collection and performance challenge protocol. All graphs represent data from *n* *=* 8 lungs. All values represent mean ± standard deviation

Lung weight significantly decreased over 18 h (0 h, 0.75 ± 0.06 kg; 18 h, 0.61 ± 0.05 kg, *p* < 0.05) and remained stable through 36 h (18 h, 0.61 ± 0.05 kg; 36 h, 0.54 ± 0.06 kg, *p* > 0.05) of cross-circulation (Supplementary Fig. 5e). Trans-pulmonary pressure gradients (TPG) were tightly maintained at 5–15 mmHg throughout cross-circulation (Supplementary Fig. 3j). Significant improvements of pressure–volume (PV) loops and dynamic compliance of injured lungs were observed throughout 36 h of cross-circulation. Initially, injured lungs displayed significant derangement of PV loops (PIP: 28.7 ± 4.4 cmH_2_O, volume: 67.9 ± 16.3 mL; injured, 0 h, red loop, Fig. 4a) and significantly reduced dynamic compliance (injured, 2.83 ± 1.86 mL cmH_2_O^−1^; control, 23.03 ± 1.84 mL cmH_2_O^−1^; *p* < 0.01; Fig. 4b). After 36 h of interventional cross-circulation, injured lungs recovered normal hysteresis (PIP: 17.9 ± 3.6 cmH_2_O, volume: 186.6 ± 23.8 mL; injured, 36 h, blue loop, Fig. 4a) similar to that of control lungs (PIP: 14.5 ± 2.1 cmH_2_O, volume: 227.5 ± 12.4 mL; control, 36 h, dark blue loop, Fig. 4a). Injured lungs maintained on 40% FiO_2_ had an average initial PaO_2_ of 36 ± 28 mmHg, corresponding to an average initial PaO_2_/FiO_2_ ratio of 90 ± 70 mmHg, and demonstrated steady improvements in gas exchange function through the first 12 h of cross-circulation (12 h, PaO_2_: 257 ± 57 mmHg, PaO_2_/FiO_2_: 643 ± 144 mmHg). Control lungs maintained an average PaO_2_ of 309 ± 57 mmHg, and PaO_2_/FiO_2_ of 773 ± 142 mmHg (Fig. 4c).

Injured and control lungs demonstrated differential capacities to exchange gas. Response to increasing minute ventilation by 100% and increasing FiO_2_ from 40 to 100% was demonstrated by differences (Δ) in baseline and challenge levels of pulmonary artery and vein hemogases (Fig. 4h), ΔpO_2_ (oxygenation, Fig. 4d, f) and ΔpCO_2_ (ventilation, Fig. 4e, g). Notably, injured lungs achieved ΔpO_2_ values of control lungs after 12 h of cross-circulation (Fig. 4d) and ΔpCO_2_ values of control lungs after 30 h of cross-circulation (Fig. 4e).

### Recovery of injured lungs during cross-circulation

**Fig. 5:**
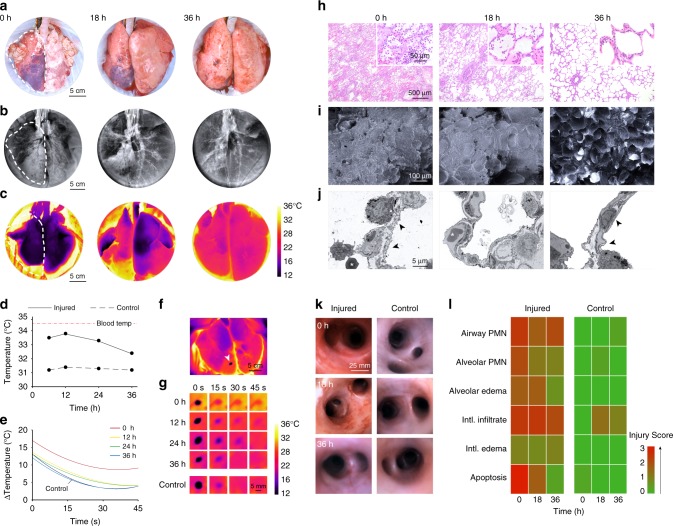
Multi-scale analysis of extracorporeal lung recovery. Macroscopic appearance: **a** photographic, **b** radiographic, and **c** thermographic analysis of lungs throughout 36 h of cross-circulation. Dotted line indicates the injured lung. **d** Average recorded surface temperature of injured and control lungs throughout 36 h of cross-circulation. **e** Difference in temperature (Δ) between hypothermic lung region and average temperature of surrounding pleura over 45 s of rewarming. Surface cooling and rewarming challenge: **f** thermographic image of lungs following contact with a conductive hypothermic probe (arrow), and **g** time lapse sequence of injured and control lungs during rewarming at 0, 12, 24, and 36 h of cross-circulation. Microscopic appearance: **h** H&E staining, **i** scanning electron microscopic, and **j** transmission electron microscopic analysis of injured lungs throughout 36 h of cross-circulation. Type I alveolar epithelial cell membranes disrupted at 0 h and restored at 36 h (arrows). Red blood cells (stars) shown in the alveolar space at 0 h and within a capillary at 18 h. **k** Bronchoscopic analysis of airways in injured and control lungs throughout 36 h of cross-circulation. **l** Lung injury scoring of injured and control lungs at 0, 18, and 36 h of cross-circulation. All graphs represent data from *n* *=* 8 lungs. All values represent mean ± standard deviation

Injured lungs had sizable areas of consolidation and discoloration suggestive of pulmonary edema and hemorrhage that were reduced after 18 h and resolved after 36 h of cross-circulation (Fig. 5a; Supplementary Fig. 5a–c). Radiography revealed severe unilateral injury with marked lobar and multi-focal opacities and the presence of air bronchograms throughout injured lungs (Fig. 5b; Supplementary Fig. 5d), while contralateral control lungs maintained normal radiographic appearance. Progressive clearance was observed throughout interventional cross-circulation, with recovery of aerated lung volume in injured lungs after 36 h.

Thermography was used to assess global and local changes in lung surface temperatures throughout 36 h of cross-circulation. Injured and contralateral control lungs were rewarmed in the normothermic organ chamber and achieved steady state surface temperatures within 30 min of reperfusion (Supplementary Fig. 3l, m; Supplementary Movie 3). Notably, through 18 h of cross-circulation, injured lungs showed more heterogeneous and elevated surface temperatures compared to contralateral regions of control lungs (Fig. 5c; Supplementary Fig. 8a). Regions of injured lungs with elevated surface temperatures correlated with areas of consolidation and hemorrhage, consistent with perfused parenchyma with diminished ventilation. After 36 h of cross-circulation, no significant differences in surface temperatures were observed across injured lungs or between injured and control lungs (Fig. 5c, d; Supplementary Fig. 8a, b). These results suggest that surface thermography is a useful diagnostic imaging modality for non-invasive assessment of extracorporeal lung recovery.

Lung surface regions (diameter: 8 mm) were topically cooled by contact with a hypothermic probe (Fig. 5f; Supplementary Fig. 8c, d). In control lungs, surface temperatures returned to baseline within 20 s (Supplementary Fig. 8e), while injured lungs consistently demonstrated protracted recovery, requiring more than 45 s to return to baseline (Fig. 5g; Supplementary Movie 7). Differences in surface temperatures between topically cooled regions and surrounding areas were quantified using video thermography and image analysis software. Temperature differences decreased exponentially over time (Fig. 5e, Supplementary Fig. 8f), with time constants for injured lungs (0 h, τ = 71.4 s; 12 h, τ = 39.1 s; 24 h, τ = 38.5 s; 36 h, τ = 36.2 s) gradually decreasing to values of time constants for control lungs (0 h, τ = 33.3 s; 12 h, τ = 30.3 s; 24 h, τ = 32.2 s; 36 h, τ = 30.3 s). These results suggest that in addition to global surface thermography, regional thermodynamic imaging may be a useful diagnostic tool for real-time, non-invasive assessment of extracorporeal organs to identify and monitor injury resulting in ventilation–perfusion mismatch.

The airways of injured lungs were inflamed and clearly distinguishable from airways of control lungs. After 18 h of interventional cross-circulation, airway bronchoscopy showed reduced erythema of injured airways, with resolving inflammation of the bronchial mucosa (Fig. 5k). After 36 h, only minimal residual inflammation was observed in airways of injured lungs. Histologic analyses of control lungs revealed normal tissue histomorphology with intact columnar bronchial epithelium and alveolar architecture, and no evidence of pulmonary edema or inflammation throughout 36 h of cross-circulation (Supplementary Fig. 5f, 7c), consistent with previous studies wherein our group demonstrated robust maintenance of healthy lungs over 36 h of cross-circulation support^24^.

In contrast, injured lungs displayed pervasive inflammation and disruption of the lung parenchyma, with extensive airway congestion, interstitial and alveolar edema, hemorrhage, leukocyte infiltration, leakage of lymphatic and plasma proteins, and cellular debris (Fig. 5h, 0 h). Scanning and transmission electron microscopy showed severe tissue derangement, exudation, and loss of stereotypical saccular structures (Fig. 5i, 0 h), with alveolar spaces containing particulate debris, extravascular erythrocytes consistent with alveolar hemorrhage, alveolar septal thickening, neutrophil extravasation, and an impaired blood–gas barrier with severely compromised respiratory epithelium, disruption of type I pneumocyte cell membranes, and atypical cytomorphology of type II pneumocytes (Fig. 5j, 0 h; Supplementary Fig. 7b). Markers of injury were drastically reduced after 18 h of interventional cross-circulation, with evidence of partial restoration of the alveolar epithelial lining, and complete resolution by 36 h of interventional cross-circulation (Fig. 5h–j, 36 h; Supplementary Fig. 7b).

Pathologic assessment of injured and control lungs was performed in a randomized blinded fashion to obtain categorical and composite lung injury scores according to a previously established scoring rubric^24^ (Supplementary Table 6). Lung injury was evaluated by quantification of polymorphonuclear (PMN) cells in airways and alveoli, early and late apoptotic cells, interstitial and alveolar edema, and interstitial infiltrates (Supplementary Fig. 6). Lung injury scores trended downward in all categories over 36 h of interventional cross-circulation (Fig. 5l; Supplementary Fig. 5g). Apoptosis was the lung injury category with the most significant reduction in injury score, decreasing from 3.0 at initiation to 0.3 after 36 h of interventional cross-circulation. Control lungs showed no changes in categorical or composite lung injury scores across 36 h of cross-circulation (Fig. 5l**;** Supplementary Fig. 5h). The difference in composite lung injury scores between injured and control lungs decreased throughout 36 h of cross-circulation from 12.1 to 5.7 (Supplementary Fig. 5i), suggesting a high degree of lung recovery.

### Regeneration of injured lungs during cross-circulation

**Fig. 6:**
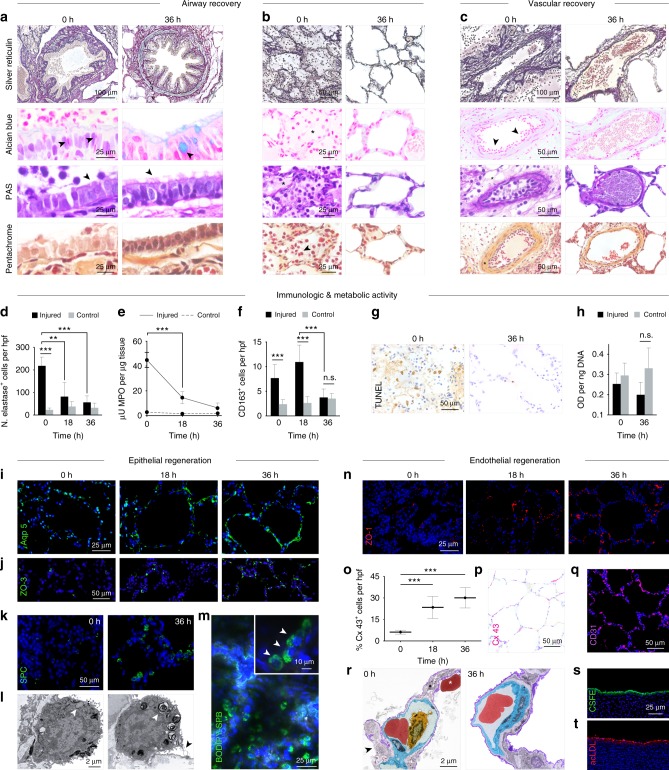
Cellular recovery and regeneration of injured lungs. Histologic analysis of airway and vascular recovery. **a** Large airway: Alcian blue 0 h with marginating PMNs (arrows) and 36 h with goblet cells (arrow), periodic acid-Schiff (PAS) stain demonstrating loss of epithelial integrity and airway cilia at 0 h and restoration of ciliated epithelial lining at 36 h (arrows). **b** Distal airway: Alcian blue and PAS stain 0 h with alveolar edema (star), pentachrome 0 h with alveolar infiltrates (arrow). **c** Vascular network: Alcian blue 0 h with marginating PMNs (arrows), PAS stain 0 h with perivascular edema (star), pentachrome 0 h with intimal thickening (star). **d** Neutrophil quantification. **e** Myeloperoxidase (MPO) activity. **f** Macrophage quantification by CD163 immunostaining. Student’s *t*-tests, ***p *<* 0.001, **p *<* 0.01, or *p* > 0.05, not significant (n.s.). **g** TUNEL staining of injured lungs at 0 h and 36 h. **h** Metabolic activity of injured and control lungs at 0 h and 36 h, Student’s *t*-test for Injured versus Control, not significant (n.s.). **i** Aquaporin 5 (Aqp 5) and (**j**) ZO-3 immunostaining of injured lungs at 0, 18, and 36 h. Recovery of surfactant in type II cells: (**k**) Loss of surfactant protein C (SPC) at 0 h, with recovery at 36 h. **l** Transmission electron micrograph (TEM) of type II cell with loss of lamellar bodies containing surfactant (white arrow) at 0 h, and TEM of type II cell with lamellar bodies containing surfactant (white arrow) with active surfactant secretion (black arrow) at 36 h. **m** Function of type II pneumocytes demonstrated by uptake of fluorescently labeled surfactant protein B (BODIPY-SBP) at 36 h. **n** ZO-1 immunostaining at 0, 18, 36 h. **o** Quantification of connexin 43^+^ cells at 0, 18, 36 h (Student’s *t*-tests, ***p *<* 0.001), with **p** connexin 43 immunostaining at 36 h. **q** CD31 immunostaining at 36 h. **r** TEM (pseudocolored) of pulmonary capillaries at 0 h showing an extravascular red blood cell (red) indicating alveolar hemorrhage (star), disruption of type I cell membrane (purple, arrow), and intra-capillary leukocyte (orange) adjacent to microvascular endothelial cell (blue), and 36 h demonstrating intact blood–gas barrier. Vascular viability and function at 36 h: uptake of **s** CSFE and **t** acetylated low-density lipoprotein (acLDL). All graphs represent data from *n* *=* 8 lungs. All values represent mean ± standard deviation

Silver reticulin staining of injured lungs demonstrated dense cellular debris and mucus within the bronchial lumen, peribronchial edema, and consolidation of alveoli, consistent with severe acute lung injury and disruption of the pulmonary epithelium. Alcian blue staining showed abundant trans-epithelial migration of neutrophils into the airway lumen through pseudostratified airway epithelium, and apparent loss of secretory vesicles containing mucus in goblet cells. Periodic acid-Schiff and pentachrome staining showed damaged, discontinuous columnar epithelium with exposed basement membrane, inflammatory cells, and loss of airway cilia (Fig. 6a). After 36 h of cross-circulation, conducting airways of injured lungs exhibited resolution of edema and inflammation, clearance of luminal debris consistent with the histologic appearance of proximal airways in control lungs, trace evidence of neutrophil infiltration, presence of mucus-filled granules in goblet cells, and intact columnar epithelium with reconstituted ciliated brush border (Fig. 6a). In the respiratory zone of injured lungs, retention of structural fibers in the alveolar extracellular matrix, clearance of alveolar edema and inflammatory cell infiltration, absence of extravascular erythrocytes, reduced perivascular edema, and restored vascular histomorphology were observed (Fig. 6b, c).

Injured lungs also contained significantly more neutrophils than control lungs, with significantly greater myeloperoxidase (MPO) activity in injured lung parenchyma (Fig. 6d, e). By 18 h of cross-circulation, neutrophil numbers and MPO activity were significantly decreased and maintained within normal ranges through 36 h of cross-circulation, consistent with histologic evaluations (Fig. 6a–c). Additionally, the number of CD163^+^ macrophages in injured lungs decreased to normal (control) levels by 36 h of cross-circulation (*p* < 0.001, Fig. 6f). Over the same period, the high initial levels of caspase 3 and TUNEL significantly decreased, and apoptotic cells were rarely observed in injured lungs, indicating clearance of dying and dead cells (Fig. 6g; Supplementary Fig. 6a, b). Interestingly, mitochondrial activity showed no significant differences in metabolism of injured or control lungs during 36 h of cross-circulation support (Fig. 6h).

As aspiration of gastric contents incurs a severe chemical injury to the respiratory epithelium, especially to the type I pneumocytes that comprise the majority of the alveolar surface^34^, the integrity of the alveolar epithelium was assessed. Expression of aquaporin 5, an integral membrane protein that functions as a water channel and marker of type I pneumocytes, was largely lost following aspiration of gastric contents but recovered during interventional cross-circulation (Fig. 6i; Supplementary Fig. 7a). Initially, total depletion of epithelial tight junctions (zonula occludens-3, ZO-3) and loss of barrier function was observed in injured lungs, but the number of tight junctions increased by 18 h of cross-circulation (Fig. 6j; Supplementary Fig. 7d). Expression of surfactant protein C (SPC), a marker of type II pneumocytes, was negligible in injured lungs but became increasingly abundant by 36 h of cross-circulation, with clear punctate distributions suggesting normal intracellular localization within lamellar bodies (Fig. 6k; Supplementary Fig. 7e). Viability and function of type II pneumocytes after 36 h of cross-circulation were confirmed by uptake of BODIPY-labeled surfactant protein B (BODIPY-SPB) delivered into distal regions of injured lungs (Fig. 6m).

Regeneration of pulmonary endothelium after aspiration of gastric contents was also assessed by analysis of the injured pulmonary vascular network, including microvascular tight and gap junctions. Immunostaining for zonula occludens-1 (ZO-1) revealed dramatic loss of tight junctions throughout the pulmonary microvasculature following injury, but recovery to normal expression levels^24^ after 36 h of cross-circulation (Fig. 6n; Supplementary Fig. 7d). Quantification of gap junction alpha-1 protein (connexin 43), a component of gap junctions that enables critical intercellular interactions, showed a similar trend of recovery to previously reported expression levels in healthy controls^35^ (Fig. 6o). Disruption of the alveolar epithelial lining and the presence of inflammatory cells, extravascular erythrocytes, and extracellular debris confirmed lung injury and a compromised blood–gas barrier (Fig. 6r; Supplementary Fig. 7b). After 36 h of cross-circulation, the pulmonary microvasculature of injured lungs appeared intact by immunostaining for CD31 (Fig. 6q), and the transmission electron micrographs (Fig. 6r; Supplementary Fig. 7b) resembled those of control lungs (Supplementary Fig. 7c), with clear restoration of the alveolar epithelial lining and intraseptal capillary geometry and architecture (Supplementary Fig. 7b). Viability of the pulmonary endothelium after 36 h of cross-circulation was confirmed by uptake of carboxyfluorescein succinimidyl ester (CFSE; Fig. 6s) and acetylated low-density lipoprotein (acLDL; Fig. 6t).

## Discussion

We describe the application of interventional cross-circulation—a strategy for prolonged, systemically regulated normothermic extracorporeal organ support—to regenerate severely damaged lungs (Supplementary Movie 1). Our results suggest that lungs injured by gastric aspiration, a common lung injury rendering donor lungs unsuitable for transplantation: (i) can be maintained on cross-circulation support without decline in lung function or tissue integrity, (ii) are amenable to repeated therapeutic interventions, and (iii) display evidence of cellular regeneration and improved function over the course of 36 h.

Aspiration of gastric contents was used as an injury model due to its: (i) reproducibility and established protocols^21,36^, (ii) severity of functional decline and tissue derangement, and (iii) clinical relevance in the context of acute lung injury and lung transplantation. Lung injury occurs in two distinct phases^37^. In the first phase (0–2 h after aspiration), damage results in the disruption of alveolar type I cell membranes and increased microvascular permeability. Alterations in the blood–gas barrier lead to extravasation of fluid and protein into the airways and alveoli, leading to pulmonary edema, decreased compliance, and progressive hypoxia^38^. In the second phase (2–6 h after aspiration), an inflammatory cascade results in neutrophil migration and the release of inflammatory cytokines such as IL-1β and TNFα by activated alveolar macrophages^39,40^. These processes in conjunction with the release of reactive oxygen species, free radicals, and proteases by infiltrating immune cells^41,42^ exacerbate lung injury.

Normothermic cross-circulation allowed for 36 h of repeated therapeutic interventions (Fig. 1b) that aided in the recovery of damaged lungs. Airway lavage was effective in ameliorating the caustic effects of gastric aspiration by removing particulate and cellular debris^43^ and by neutralizing pH, thereby decreasing proteolytic enzyme concentration and activity (Fig. 3, a–h). While the delivery of exogenous surfactants effectively replenishes depleted and degraded endogenous surfactants and improves compliance^44^, beneficial effects only occur after inhibitory plasma proteins and fluid are removed by lavage^45^. Accordingly, in our therapeutic intervention regimen, surfactant replacement was performed after bronchoalveolar lavage. Alveolar recruitment maneuvers improve ventilation–perfusion matching and arterial oxygenation^46^, and have been shown to improve clinical outcomes^47^. Administration of surfactant enhances the spatial distribution of lung ventilation via recruitment, which recovers gas exchange^48^ through restoration of ventilation–perfusion match.

Pepsin, a biomarker of gastric aspiration^49,50^, has recently been linked to allograft injury and bronchopulmonary dysplasia in transplant patients^51^. Pepsin-induced cytotoxicity and release of IL-6 and IL-8 peaks at pH 1.5–2.5^52^. After 12 h of cross-circulation and repeated bronchoalveolar lavage, pepsin concentrations in BAL fluid significantly decreased, pH of BAL fluid normalized to pH > 5, and IL-6 and IL-8 concentrations decreased significantly (Fig. 3, Supplementary Fig. 4). Elevated levels of IL-8 in BAL fluid perpetuate aspiration injury via IL-8 dependent mechanisms^53,54^. Notably, initially high levels of IL-8 in injured lungs normalized to control levels after 12 h of interventional cross-circulation. Similarly, levels of other key inflammatory cytokines (e.g., IFNγ, IL-1β, and TNFα) decreased significantly in BAL fluid from injured lungs over the course of 36 h of interventional cross-circulation (Fig. 3h, Supplementary Fig. 4c, Supplementary Table 9). Gastric aspiration also increases susceptibility to respiratory pathogens and bacterial infections^55,56^. Microbial cultures of BAL fluids from injured and control lungs contained normal respiratory flora and were negative for pathologic growth, demonstrating that repeated interventions in extracorporeal lungs can be performed without introducing pathogens.

Over 36 h of cross-circulation, injured lungs showed recovery of mechanical compliance to 68% of the average compliance of control lungs—a more than six-fold improvement. Significant changes in pressure–volume (PV) loops were consistent with this degree of recovery and did not resemble stereotypical PV loop hysteresis until after 24 h of cross-circulation (Fig. 4a, b). The initial average PaO_2_/FiO_2_ of injured lungs was <90 mmHg (11% of the average PaO_2_/FiO_2_ in control lungs) and increased to >640 mmHg (83% of the average PaO_2_/FiO_2_ in control lungs). Notably, following 36 h of cross-circulation there was no significant difference between the PaO_2_/FiO_2_ of injured and control lungs (Fig. 4c).

Lung injury scores consistently decreased across all categories, with the greatest reductions in alveolar PMN cells, edema, and apoptosis (Fig. 5l, Supplementary Fig. 5g–i; Supplementary Fig. 6). Notably, we demonstrated: (i) longitudinal expression and regeneration of cell surface markers of the pulmonary epithelium and endothelium, and tight and gap junction proteins, (ii) recovery of functional subcellular structures (e.g., intracellular vesicles), and (iii) cellular function and integrity throughout 36 h of extracorporeal support (Fig. 6).

Acute lung injury affects the alveolar epithelium and microvascular endothelium, and specifically reduces the expression of aquaporin 5, which plays an essential role in water transport^57^. Following aspiration, aquaporin 5 was drastically reduced in injured lungs. After 36 h of interventional cross-circulation, aquaporin 5 expression was similar in injured and control lungs (Fig. 6i, Supplementary Fig. 7a). Additionally, epithelial tight junction protein ZO-3 and microvascular tight junction protein ZO-1 displayed similar expression patterns of recovery, with very low initial levels and significantly higher levels reaching those in control lungs after 36 h of cross-circulation. These data, along with decreased lung weight (Supplementary Fig. 5e), improved gas exchange and compliance (Fig. 4), and decreased alveolar edema observed by light and electron microscopy (Figs. 5, 6), strongly suggest that interventional cross-circulation enabled significant cellular regeneration in the airway and vascular compartments, resulting in functional restoration of the alveolar-capillary membrane in extracorporeal lungs.

Decrease in surfactant protein C, a marker of type II pneumocytes^58^, in distal regions of injured lungs resulted in the loss of surfactant-filled lamellar bodies in type II cells. After 36 h of interventional cross-circulation, significant increases in SPC expression and restoration of intracellular surfactant-filled lamellar bodies were observed (Fig. 6k, l). Airway regeneration was demonstrated by the restoration of: (i) contiguous type I cell membranes, (ii) a ciliated epithelial brush border, and (iii) subcellular structures including mucus-filled vesicles in airway goblet cells and surfactant-filled lamellar bodies in alveolar type II cells. These data suggest extensive epithelial cellular regeneration in the airway that has not been demonstrated by other lung support systems (Supplementary Table 1–3). In addition, increased expression of connexin 43 (Fig. 6o, p) correlated with the recovery of severely damaged lungs over 36 h of support, during which the injured pulmonary vascular network not only remained viable but also substantially regenerated. We hypothesize that the degree of cellular regeneration achieved is the result of the beneficial effects of therapeutic interventions, physiologic homeostatic maintenance and prolonged support provided by cross-circulation with a living host.

The present study has several limitations: (1) While the use of inbred swine enabled recovery and regeneration of injured lungs with minimal donor–recipient immunologic cross-reactivity, future studies will need to assess the effects of immunosuppression (necessary for lung transplantation) on lung recovery. (2) This study assessed recovery from lung injury 6 h after gastric aspiration, a period within which a severe inflammatory response develops. Clinically, aspiration events often precede interventional recovery by more than 6 h, and additional studies are needed to extend this approach to lungs enduring a longer exposure to aspiration pneumonitis. Nevertheless, the injury model and therapeutic regimen used in this study may also represent an opportunity to inform clinical practice in cases of known or suspected gastric aspiration (e.g., in trauma patients, patients found down prior to admission to the hospital, or patients intubated in the field by first responders). (3) To maintain appropriate controls, only a single lung was injured in each experiment, while in the clinical setting, patients frequently develop bilateral aspiration pneumonitis. (4) According to multiple assessment criteria, including radiography, pressure–volume loops, compliance, histologic analyses, lung injury scores, and cell-specific functional markers such as aquaporin 5 and surfactant protein C, injured lungs improved to reach transplantation criteria, but did not achieve full recovery. Future studies should investigate the functional capacity of lungs following transplantation, and if longer support and recovery times would be of greater benefit.

Beyond demonstrating extracorporeal regeneration of severely damaged lungs, this study resulted in the development of technical methodologies. During ex vivo organ perfusion, advanced thermography^59^ can be used for the identification of flow and circuit failures^60^, and enable real-time visualization of rewarming during lung reperfusion^24^. We extended thermography to assessing global surface temperatures and conducting localized thermal recovery tests as a diagnostic for non-invasive evaluations of injured extracorporeal lungs. We also found that exosome concentrations in BAL fluids from injured lungs were elevated and increased over the first 12 h of cross-circulation. As injured lungs recovered, exosome concentrations decreased and approached levels in control lungs (Fig. 3g). Profiling of exosome surface markers and cargo was beyond the scope of this study but represents an opportunity for future investigation, as exosomes in BAL fluid may represent a biomarker to diagnose, grade, and monitor the trajectory of lung injury and recovery.

Envisioned clinical applications of an interventional cross-circulation platform are motivated by the thousands of patients who die every year awaiting suitable organs for transplantation. The recovery of an unsuitable organ may represent their only chance for transplantation and survival. We envision that prolonged extracorporeal support by interventional cross-circulation could be used to recover and regenerate severely damaged organs (Supplementary Fig. 9). Furthermore, as humanized swine and immunosuppressive regimens are being developed for applications in xenotransplantation, the utility of a non-human host should also be assessed in a xenogeneic cross-circulation system for the recovery of human donor organs unacceptable for transplantation.

In summary, we report: (i) the development of an interventional extracorporeal organ support platform capable of systemic regulation, (ii) the use of non-invasive diagnostics (dynamic surface thermography, exosomes), and (iii) evidence of cellular regeneration of severely damaged extracorporeal lungs. Furthermore, we demonstrate: longitudinal expression and regeneration of cell markers of the pulmonary epithelium and endothelium, recovery of functional subcellular structures, and cellular function and integrity throughout 36 h of interventional cross-circulation. Our results also establish a benchmark for molecular and cellular analytics of extracorporeal lung regeneration (Supplementary Table 4). We envision that interventional cross-circulation may be used to investigate regeneration of other damaged organs (e.g., hearts, kidneys, livers), where expansion of donor pools through the salvage of severely damaged organs could lead to more organ transplantations.

## Methods

### Animals

Sixteen miniature swine (eight donor–recipient pairs) from a herd of partially inbred swine were utilized in this study. Pairs with well-defined major histocompatibility complex (MHC) loci were selected. Immunogenetic characteristics and selective breeding methods have been previously reported^61^. Animals were 5–8 months of age, with a median weight of 39.2 kg (range: 30.6–53.5 kg). All studies complied with the relevant ethical regulations for animal testing and research. Approval for this study was received from the Institutional Animal Care and Use Committee at Columbia University. Animal care and procedures were conducted in accordance with the US National Research Council of the National Academies *Guide for the Care and Use of Laboratory Animals*, 8th Edition.

### Standardization of gastric contents for gastric aspiration injury model

Gastric contents were collected from six swine (of the same herd) deprived of food for 12 h and standardized according to methods previously described^21,25,36^. Briefly, animals (*n* *=* 6) were sedated, and an orogastric tube (36 Fr) was inserted and engaged to suction. Gastric contents collected from swine were admixed, strained through sterile surgical gauze to remove large particulates, normalized with sterile hydrochloric acid to a standard pH of 2.00 ± 0.05, aliquotted, stored at – 80 °C, and thawed immediately prior to experimental use (Supplementary Fig. 1a). The concentration of total non-sulfated bile acids (416 ± 208 µM) and presence of pepsin in standardized gastric contents were measured and confirmed using a Bile Acid Assay Kit (Sigma–Aldrich) Pepsin ELISA kit (LSBio) according to the manufacturer’s instructions. Microbial cultures of gastric contents were negative for all aliquots.

### In vivo lung injury

Donor swine (*n* *=* 8) underwent general anesthesia via intramuscular induction with Telazol (5 mg kg^−1^, Zoetis) and buprenorphine hydrochloride (0.03 mg kg^−1^, Hospira). Following induction and intubation, the anesthetic regimen consisted of continuous infusions of midazolam (1.5 mg kg^−1^ h^−1^, Akorn), fentanyl citrate (0.1 mg kg^−1^ h^−1^, West-Ward), and inhaled isoflurane (1–5% in oxygen, Henry Schein). Gastric aspiration injury was adapted from previously established injury models^21,25,36^. Following donor sedation, a bronchoscope was introduced into the lower lobe of a single lung, and the location of the bronchoscope tip was confirmed by chest X-ray. Standardized gastric contents (2 mL kg^−1^; pH 2) were delivered bronchoscopically while withdrawing the tip of the bronchoscope to a position approximately 1 cm distal to the carina to avoid introduction of gastric contents into the contralateral (control) lung. This distance was established prior to delivery of standardized gastric contents by measuring the length of the bronchoscope as it was advanced past the carina. After delivery, animals remained under general anesthesia and standardized gastric contents remained in donor lungs for 6 h to allow development of a unilateral acute lung injury in vivo.

### Validation of gastric aspiration injury

Following unilateral bronchoscopic delivery of standardized gastric contents into donor lungs, donor vitals and ventilator settings were continuously recorded. Chest X-rays, airway bronchoscopic images, and hemogases were obtained at baseline and every 2 h for 6 h. Donor blood samples were collected at baseline and 6 h after gastric aspiration to assess serum levels of inflammatory markers (GM-CSF, IFNγ, IL-1α, IL-1β, IL-1rα, IL-2, IL-4, IL-6, IL-8, IL-10, IL-12, IL-18, P-Selectin, and TNFα) by multiplex array analysis (Eve Technologies) and hemolytic markers (D-dimer, fibrinogen, and plasma free hemoglobin) by commercially available enzyme-linked immunosorbant assays (ELISAs). A list of the ELISA kits used is provided in Supplementary Table 11. BAL fluid samples were collected from injured and control lungs at baseline and 6 h after gastric aspiration to assess the airway response to gastric aspiration. Inflammatory markers (GM-CSF, IFNγ, IL-1α, IL-1β, IL-1rα, IL-2, IL-4 IL-6, IL-8, IL-10, IL-12, IL-18, and TNFα) in BAL fluid samples were quantified by multiplex array analysis (Eve Technologies), M30 by commercially available ELISA, and cellular contents in BAL fluid were analyzed by Kwik-Diff staining or periodic acid-Schiff staining. Gross images of injured lungs were obtained prior to explant using an interchangeable lens digital camera (Sony).

### Histopathologic analyses of in vivo lung injury

Tissue samples were collected from lung segments (lower lobes and posterior segments of upper lobes) of injured and control lungs according to a pre-determined lung map (Supplementary Fig. 4a) to avoid sampling bias and enable randomized sampling of injured and control lungs. A surgical stapler with medium/thick reloads was used to obtain lung samples. Specimens were immediately fixed in cold phosphate-buffered 4% paraformaldehyde for 48–72 h, embedded in paraffin, and sectioned at 3 μm or 5 μm thickness. All sections were stained with hematoxylin and eosin (H&E) and pentachrome and examined via light microscopy. Immunohistochemical staining was conducted for neutrophil elastase (airway and alveolar polymorphonuclear cells) and caspase 3 (early apoptotic cells). Late apoptosis was detected on tissue sections using a TdT-mediated dUTP nick end-labelling (TUNEL) assay. Pathologic review was performed by a pulmonary pathologist blinded to the samples and study protocol. All slides (H&E, immunohistochemical staining for neutrophil elastase and caspase 3, and TUNEL) were randomized, arbitrarily numbered, and delivered to the pathologist without reference to experimental time points or conditions^55^. To evaluate gastric aspiration injury, a modified lung injury score was developed from previously described methods^56,57^. Scoring (Supplementary Table 6) was based on airway polymorphonuclear cells per high-power field (hpf) (×40; 0.2 mm^2^; 0 ⇒ 0, 1 ⇒ 1–25, 2 ⇒ 26–50, 3 ⇒ > 50), alveolar polymorphonuclear cells per hpf (×40; 0.2 mm^2^; 0 ⇒ 0, 1 ⇒ 1–25, 2 ⇒ 26–50, 3 ⇒>50), alveolar edema (0 ⇒<5% of all alveoli contained edema fluid, 1 ⇒ 6–25%, 2 ⇒ 26–50%, 3 ⇒>50%), interstitial infiltrate (lymphocytes and neutrophils in the interstitium around vessels and airways and in alveolar septa and pleura; 0 ⇒ none per hpf, 1 ⇒ < 50 per hpf, 2 ⇒ 50–100 per hpf, 3 ⇒ > 100 per hpf), interstitial edema (perivascular and peribronchial spaced expanded with edematous fluid; 0 ⇒ none per hpf, 1 ⇒ 1x width vessel media, 2 ⇒ ≥ 2x width vessel media), and total number of apoptotic cells per hpf (×40; 0.2 mm^2^; 0 ⇒ < 2, 1 ⇒ 3–5, 2 ⇒ 6–10, 3 ⇒>10). Numbers for early and late apoptosis, as quantified by caspase 3 and TUNEL, were combined into a single apoptosis score. Lung injury scores of injured and control lungs were compared at 6 h after gastric aspiration as an index of lung injury following unilateral delivery of standardized gastric contents.

### Procurement of injured lungs

Prior to skin incision, animals were prepped and draped in standard sterile fashion and antibiotics were given (Cefazolin, 30 mg kg^−1^; WG Critical Care). Following median sternotomy, a heparin bolus (30,000 U) was administered intravenously (Sagent Pharmaceuticals), and the main pulmonary artery (PA) was cannulated. Autologous blood was collected via the PA cannula. Blood was stored in citrate-phosphate-dextrose collection bags (Chinook Medical) at 8 °C. Following exsanguination and presence of a non-perfusing cardiac rhythm, a cold anterograde low-potassium dextran flush (Perfadex, Vitrolife) with alprostadil (25 mg kg^−1^, Prostin VR Pediatric, Pfizer) was delivered via the main PA cannula, and the inferior vena cava and left atrial appendage were transected. Concurrently with the flush, topical cooling with sterile ice was applied. Once the flush was complete additional dissection was completed, and the lungs were inflated to a sustained airway pressure of 15 cmH_2_O. With the lungs inflated and all lobes recruited, the trachea was stapled (Endo GIA, Medtronic), and the heart and lungs were explanted en-bloc. Working on a sterile back table, the heart was removed. Cannulation with the bio-bridge was facilitated by leaving a circumferential cuff (height of 3–5 mm) of the left atrium. Next, a retrograde flush with cold Perfadex (20 mL kg^−1^) was performed. To prepare the bio-bridge, the aortic arch was first dissected, and the left subclavian and brachiocephalic branches were stapled. A sufficient length of aorta was left on either side of these vessels to facilitate placement of the pulmonary vein cannula into one end and attachment to the left atrial cuff at the other (Supplementary Fig. 2a–f).

### Lung cannulation

To enable inflow via the pulmonary artery, an 18–20 Fr cannula was secured using a purse-string 5–0 prolene suture, 0 silk ties, and TourniKwik tourniquet (Medtronic). To enable ventilation, a 7.5–8.0 mm cuffed endotracheal tube (Sheridan) was placed within the trachea and secured with 0 silk ties.

### Single lung venous cannulation

The aortic arch, serving as an endothelialized biological bridge (bio-bridge) between the lung and the extracorporeal circuit, was connected to the left atrial cuff with a running 6–0 prolene suture (Ethicon). Outflow through the bio-bridge was facilitated by the placement of a 36 Fr crenellated drainage cannula. This venous drainage cannula was secured in place with 2–0 Ti-Cron ties (Covidien) or 0 silk ties. To assess unilateral gas exchange function of injured or control lungs, two 20 G × 12 cm cannulas (Arrow International) (Supplementary Fig. 2e) were placed through the wall of the bio-bridge (aortic arch) and inserted into the left and right pulmonary veins. The position of each single lung venous cannula was confirmed by X-ray (Fig. 1d; Supplementary Fig. 2g). Intravenous tubing (diameter: 3 mm) was connected to each single lung venous cannula, led out of the organ preservation chamber, and connected to a venous blood port (Supplementary Fig. 2h) to enable single lung venous blood hemogas analysis.

Once cannulated, lungs were flushed with cold normal saline to remove air and prime the cannulas. Within the preservation chamber (XVIVO), a top-loading balance (Denver Instrument Company) was placed, followed by a double-lined organ basin with warm recirculating saline (Supplementary Fig. 3). Lungs were positioned in the prone position within the basin, and the PA, PV, and endotracheal tube were secured. Whole blood collected during donor lung procurement was used to prime the circuit. Next, the circuit was de-aired, and connected to extracorporeal lungs. Initial flow rates were set to 5–10% of the estimated cardiac output, with a target pulmonary artery pressure of <15 mmHg, and pulmonary vein pressure of 3–5 mmHg.

### Cross-circulation

Recipient swine (*n* *=* 8) underwent sedation and general anesthesia in the same fashion as donor swine. All recipients underwent endotracheal intubation and were continuously ventilated throughout the duration of the study. Prior to skin incision, antibiotics (Cefazolin; 30 mg kg^−1^) was given and re-dosed every 8 h. An auricular arterial line (Arrow International) was placed for hemodynamic monitoring and periodic blood sampling. Femoral arterial lines were used if auricular placement was unsuccessful. Exposure of the left and right internal jugular veins was accomplished via bilateral neck cut-downs. Cannulation with 16–18 Fr catheters was performed in standard fashion using the Seldinger technique. A heparin bolus (15,000 U) was administered just prior to cannulation. Immediately prior to initiation of cross-circulation, 1 g methylprednisolone (APP Pharmaceuticals) and 500 mg calcium chloride (Hospira) were intravenously administered to recipient swine. Donor blood was used to prime the circuit, and tubing was spliced to connect the recipient swine to the extracorporeal circuit, marking the start of cross-circulation (Supplementary Fig. 3a, b).

Ventilation was initiated within the first 10 min of cross-circulation while the circuit and lungs were allowed to acclimate to ambient temperature. Initial settings were as follows: volume control mode, respiratory rate 6–8 bpm, tidal volume (TV) 6–8 mL kg^−1^, positive end-expiratory pressure (PEEP) 5 cmH_2_O, and FiO_2_ 40% (Oxylog 3000 plus; Dräger). To recruit atelectatic lung regions, ventilatory maneuvers included increasing PEEP (up to 10 cmH_2_O), TV, and by performing inspiratory hold maneuvers (up to 25 cmH_2_O). Manual recruitment maneuvers were used if ventilation strategies failed. Thermal images of the lung were captured at periodic time points throughout the duration of each procedure using a FLIR T430sc infrared camera.

Circuit elements consisted of the following: main console (Jostra HL-20 pump console; Maquet), disposable pump (Rotaflow centrifugal pump; Maquet), softshell reservoir (Maquet), and three-eighths inch tubing (Smart coated tubing; LivaNova). Continuous monitoring and recording (VIPER clinical interfacing software; G2 v1.26.4; Spectrum Medical) of pressures (pulmonary artery and vein), flows (pulmonary artery and vein), and temperatures were performed. The recipient was maintained on a continuous heparin infusion throughout the duration of cross-circulation (initial rate of 25 U kg^−1^ h^−1^), and activated clotting time (ACT) was measured using a whole blood micro-coagulation system (Hemochron; Accriva Diagnostics). Adjustments to the heparin drip were made in order to maintain a target ACT value of 250–350 s. Physiological parameters of the recipient were continuously monitored and recorded using a multi-parameter Advisor vital signs monitor (SurgiVet). These included: heart rate, electrocardiogram, blood pressure (cuff and arterial line pressure), mean arterial pressure (MAP), oxygen saturation (SpO_2_), end-tidal CO_2_, temperature, and respiratory rate. Analysis of recipient vitals, hemogas, biochemistry, hemolysis, electrolytes, and serum cytokines throughout 36 h of cross-circulation is provided in Supplementary Table 7, 8.

### Therapeutic interventions

Recovery of injured lung function during cross-circulation was facilitated by a series of interventions within the scope of standard clinical practice. A standardized 30-min interventional procedure (Fig. 1b) was performed on injured lungs at 0, 2, 4, 6, 12, and 18 h of cross-circulation: three cycles of bronchoalveolar lavage (15 min), surfactant replacement (5 min), and alveolar recruitment (10 min), followed by functional assessments of injured and control lungs by single lung analysis of hemogases and dynamic compliance. All airway bronchoscopy, bronchoalveolar lavage, and surfactant delivery were performed with a 3.8 mm flexible bronchoscope (aScope 3, Ambu).

### Bronchoalveolar lavage

At 0, 2, 4, 6, 12, and 18 h of cross-circulation, bronchoalveolar lavage was used to clear gastric contents, secretions, and cellular debris from injured lungs. Sterile normal saline (5 mL segment^−1^) was bronchoscopically injected into each injured lung segment and immediately aspirated. To therapeutically intervene in an injured porcine lung (e.g., the left porcine lung consists of two lobes (cranial and caudal) with a total of ten bronchopulmonary segments^62^) the volume of lavage fluid per therapeutic intervention was ~50 mL. Five-minute intervals were interspersed throughout the lavage procedure to minimize interruptions in lung ventilation. Accordingly, similar to previously described protocols^11^, the total volume of lavage fluid was ~300 mL.

### Surfactant replacement

Immediately following bronchoalveolar lavage of injured lungs at 0, 2, 4, 6, 12, and 18 h of cross-circulation, exogenous surfactant (bovine lipid extract surfactant, BLES; Biochemicals) was delivered bronchoscopically into injured lungs. Bovine lipid extract surfactant was stored at –20 °C and warmed to room temperature immediately prior to administration (Supplementary Fig. 8g). During each therapeutic intervention, one vial of bovine lipid extract surfactant (3 mL; 27 mg phospholipids mL^−1^) was administered and dispersed throughout injured lungs. Accordingly, injured lungs received a total dose of bovine lipid extract surfactant of 12.5 mg phospholipid kg^−1^ (donor swine body weight), consistent with surfactant replacement strategies previously described^11^.

### Alveolar recruitment

Following bronchoalveolar lavage and surfactant replacement, alveolar recruitment maneuvers were performed to transiently increase the airway positive pressure gradient, resulting in the opening of non-aerating lung units and increased vital capacity, while avoiding barotrauma and the deleterious effects of hyperinflation. Ventilation strategies included increasing tidal volume by 15–20%, increasing positive end-expiratory pressure (PEEP) up to 12.5 cmH_2_O, and performing inspiratory hold maneuvers (sustained inflation) up to 25 cmH_2_O. When ventilation strategies were insufficient, manual recruitment maneuvers were performed by an experienced lung transplant surgeon in a manner consistent with standard practices used during clinical lung transplantation. During inspiration, gentle manual compression was applied to areas of aerated lung, thereby directing ventilation and recruitment toward areas of non-aerating atelectatic lung. Peak inspiratory pressures were monitored and maintained below 30 cmH_2_O. Accordingly, during each therapeutic intervention, injured lungs were subjected to 10 min of alveolar recruitment maneuvers prior to returning all ventilator settings to a lung protective ventilation strategy. Injured lungs underwent a total alveolar recruitment time of ~60 min.

### Recipient monitoring and blood analysis

Hourly blood samples were drawn from an auricular arterial line (or femoral arterial line if auricular placement was unsuccessful), and analyzed using an epoc point-of-care blood analysis system (Epocal). Additional samples were collected in test-specific specimen vials (BD Vacutainer) every 4 h to obtain the following: complete blood count, liver function tests, basic metabolic panel, lactate dehydrogenase, and coagulation panels (Antech Diagnostics). Additional hemolytic markers (D-dimer, plasma free hemoglobin fibrinogen,) and inflammatory markers (GM-CSF, IFNγ, IL-1α, IL-1β, IL-1rα, IL-2, IL-4, IL-6, IL-8, IL-10, IL-12, IL-18, P-selectin, and TNF-α) were analyzed in triplicate by the Discovery Assay Pig Cytokine Array (Eve Technologies) or by commercially available enzyme-linked immunosorbant assays (ELISAs) according to the manufacturer’s instructions. A list of the ELISA kits used is provided in Supplementary Table 11.

### Extracorporeal lung monitoring and functional analyses

Blood samples were drawn from the main pulmonary artery cannula (blood entering both lungs), main pulmonary vein cannula (blood exiting both lungs), and single lung venous cannulas (blood exiting each lung) every 6 h and analyzed in the same fashion as recipient blood samples. Temporary changes in ventilation settings (minute ventilation and FiO_2_) were made according to a lung performance challenge protocol in order to further assess extracorporeal lung performance every 6 h. Immediately following the challenge, a subsequent set of blood samples were drawn from the pulmonary artery and pulmonary vein cannulas. Dynamic compliance (C_dyn_ = TV/(PIP – PEEP); mL cmH_2_O^−1^) of extracorporeal lungs was calculated every 6 h. Pressure and volume recordings of lungs were measured using a custom measurement system consisting of sensors for pressure and airflow and a data acquisition device (Arduino Uno). Data collected with this system were processed, and pressure–volume loops were generated using MATLAB v. R2016b (Mathworks). Lung weight was obtained every 6 h using the top-loading scale (Denver Instrument Company) within the organ chamber. To ensure accurate recordings, the basin and contents were tared (zeroed) at each time point. X-ray images of lungs were obtained using a PXP-16HF portable X-ray unit (United Radiology Systems) at 2.2 mAs and 90 kVp.

### Single lung ventilation

To enable single lung ventilation and single lung compliance measurements, an endobronchial blocker (7 Fr; Cook Medical) (Supplementary Fig. 2i–k) was inserted into the left or right main stem bronchus under video bronchoscopy (Fig. 1h; Supplementary Fig. 2l). The balloon was then expanded in the main stem bronchus to occlude airflow (Supplementary Fig. 2m) and enable single lung ventilation of the contralateral lung. Additionally, when obtaining measurements from the left lung the endotracheal tube was advanced slightly and the cuff was inflated to enable the occlusion of the right tracheal bronchus (i.e., pig bronchus). Once occluded, tidal volumes were reduced by half and pressure–volume loops and dynamic compliance of single lungs (injured or control) were acquired during single lung ventilation.

### Bronchoalveolar lavage fluid analysis

BAL fluid samples were collected by wedging a 3.8 mm flexible bronchoscope (aScope 3, Ambu) into a subsegmental bronchus of an injured or control lung. Sterile normal saline (5 mL) was injected, aspirated, and collected in a sterile specimen trap (Busse Hospital Disposables). Gross images of BAL fluid samples were acquired throughout 36 h of cross-circulation using an interchangeable lens digital camera (Sony). The pH of BAL fluid samples was measured with a glass double junction microelectrode (Fisher Scientific). BAL fluid samples were centrifuged at 2164 × *g* for 10 min at 4 °C. Snap-frozen supernatants were stored at –80 °C until further processing.

### Cytopathology

The cellular contents of BAL fluid samples were visualized by Kwik-Diff staining or periodic acid-Schiff staining of BAL fluid smears and quantified using a previously described automatic cell counting algorithm (ImageJ)^63^ (Supplementary Fig. 4b).

### Molecular pathology

Inflammatory markers (GM-CSF, IFNγ, IL-1α, IL-1β, IL-1rα, IL-2, IL-4, IL-6, IL-8, IL-10, IL-12, IL-18, M30, and TNFα) in BAL fluid samples collected from injured and control lungs were analyzed in triplicate using either the Eve Technologies Discovery Assay Pig Cytokine Array or commercially available enzyme-linked immunosorbant assays (ELISAs) (Supplementary Table 9). Total protein concentrations of BAL fluid samples were determined using a protein assay (Pierce Coomassie Bradford Protein Assay Kit, ThermoFisher Scientific) with bovine serum albumin as a standard. Pepsin concentrations in BAL fluid samples were measured using a commercially available porcine pepsin ELISA kit (LS Bio) according to the manufacturer’s instructions.

### Microbial cultures

BAL fluid samples collected from injured and control lungs throughout 36 h of interventional cross-circulation were submitted to a diagnostic laboratory (Antech Diagnostics; Lake Success, NY) for bacterial cultures. Microbial cultures of BAL fluid samples collected from injured lungs (*n* *=* 8) and control lungs (*n* *=* 8) were negative for pathologic growth after 72 h in all cases. Of note, all contents within the organ chamber were sterile, and proper sterile technique was stringently adhered to during all tissue sampling and lung manipulation. Between each use the bronchoscope was disinfected and sterilized according to established protocols to prevent bronchoscopy-associated infection^64^.

### Exosomes

BAL fluid samples collected from injured and control lungs throughout 36 h of interventional cross-circulation were submitted for analysis of exosome total concentration, concentration as a function of size, distribution of mean particle size (diameter), and imaging (Supplementary Movie 8) (NanoSight Tracking Analysis, System Biosciences).

### Acetylated LDL uptake assay

To assess the function and viability of the vascular endothelium^65^, at the conclusion of each procedure, biopsies of the pulmonary arteries and veins were collected and placed in a 96-well plate (BD Falcon). Acetylated LDL, Alexa Fluor 594 conjugate (ThermoScientific L35353) was diluted 1:200 in 1X DMEM/F12K (50/50) cell culture media (Corning). Wells received 150 μL of media containing acetylated LDL. Media alone (150 μL) was added to control wells containing vascular biopsies from each source. The multi-well plate was then covered and incubated at 37 °C with gentle shaking for 4 h. Next, samples were washed five times with phosphate-buffered saline followed by fixation in cold phosphate-buffered 4% paraformaldehyde for 48 h. Samples were then embedded in paraffin, sectioned (5-μm thickness), de-paraffinization, stained with DAPI, and examined using a fluorescent microscope (Olympus FSX100).

### Carboxyfluorescein succinimidyl ester uptake assay

To assess the viability of the pulmonary endothelium, carboxyfluorescein succinimidyl ester (CFSE; Affymetrix, eBioscience) was reconstituted in dimethyl sulfoxide at a concentration of 1.06 M and protected from light. At the conclusion of each procedure, CFSE was delivered via intravascular injection with a 20 Ga needle. Following 15 min incubation, samples were washed five times with phosphate-buffered saline, fixed in cold phosphate-buffered 4% paraformaldehyde for 48 h, fixed, embedded, de-paraffinized, and imaged using an Olympus FSX100 microscope.

### BODIPY-surfactant uptake assay

To assess the functional uptake and viability of type II pneumocytes^66^, delivery of a fluorescent BODIPY-labeled surfactant protein B (BODIPY-SPB) was performed into the distal regions of injured lungs. A flexible video bronchoscope and Renegade microcatheter system (Boston Scientific) enabled targeted delivery. Lung samples were collected after 30 min using a surgical stapler (Medtronic). Samples were then dissected, rinsed (Dulbecco’s phosphate-buffered saline), and imaged immediately with an Olympus FSX100 microscope. Further resolution was obtained by incubating injured lung tissue samples (2 × 2 mm) collected after 36 h of cross-circulation with 20 ng mL^−1^ BODIPY-SPB for 30 min in a normoxic incubator at 37 °C. Next, specimens underwent staining for 10 min with CellMask Deep Red plasma-membrane stain, followed by five washings for 1 min each (Dulbecco’s phosphate-buffered saline). Images were acquired with a two-photon confocal laser scanning microscope (Leica TCS SP8). Visualization of the fluorescent signal in a punctate pattern (lamellar bodies) within type II pneumocytes indicated surfactant uptake.

### Metabolic activity assay

To assess changes in lung metabolism, lung tissue samples from injured and control lungs were collected at 0 and 36 h of cross-circulation. Lung parenchyma (~250 μL tissue volume; *n* = 8) was dissected in standard sterile fashion. Fine mincing and gentle homogenization was performed prior to sample placement in a 96-well plate. Alamar Blue Assay^67^ (ThermoFisher) reagent was diluted 1:10 in DMEM cell culture media with 10% fetal bovine serum. Next, 100 μL Alamar Blue reagent was added to wells containing lung sample homogenates. In addition, Alamar Blue alone (100 μL) was added to empty wells (negative controls). The multi-well plate was covered and incubated at 37 °C with gentle shaking for 2 h. Following incubation, well contents were transferred into new 96-well plates, and absorbance (optical density, OD) was measured at 570 nm and normalized to absorbance at 600 nm. Following the metabolic activity assay, the DNA content of each sample was quantified using a Quanti-iT PicoGreen dsDNA Assay kit (Invitrogen) according to the manufacturer’s instructions. Sample digestion was carried out in 250 µg papain mL^−1^ at 60 °C for 4 h and mixed with PicoGreen reagent. To quantify DNA using a standard curve, fluorescence emission was measured at 520 nm with excitation at 480 nm.

### Myeloperoxidase activity assay

To study the activity of myeloperoxidase (MPO), tissue samples were collected at multiple time points throughout cross-circulation. Lung tissue sections (≥10 mg) were snap frozen in liquid nitrogen and stored at –80 °C. MPO assay was conducted according to the manufacturer’s instructions (Abcam, ab111749).

### Thermography

Thermal images of the lungs were captured at selected time points throughout the cross-circulation procedure using a T430sc infrared camera (FLIR) and ResearchIR v. 4.40.6 software (FLIR). Lung surface temperatures were averaged over isomorphic regions of interest in injured and control lungs and plotted relative to the average blood temperature during cross-circulation.

### Hypothermic probe test

To standardize background reflectance and atmospheric effects^68^ prior to each hypothermic probe test, the lung was maintained for a minimum of 10 min enclosed within the organ chamber to allow lung surface temperatures to equilibrate to ambient conditions. For each hypothermic probe test, real-time calibrated thermal videos of the hypothermic probe test were recorded at 60 frames s^−1^. The organ chamber was opened, and a hypothermic probe (aluminum alloy tip, diameter: 8 mm) maintained at 4 °C was immediately and consistently brought into physical contact with the pleural surface of injured or control lungs at pre-determined, designated locations for a duration of 1 s. Contact pressures were calibrated and then standardized across all tests and time points using a manual push–pull force gauge (Topac). Thermal videography of lung regions topically cooled by thermophysical contact with the hypothermic probe was acquired for non-invasive quantitative assessment of localized rewarming in injured and control lungs (Supplementary Movie 7). Thermographic images were extracted from raw video sequences at 15 s intervals. Differences in surface temperatures between topically cooled regions and surrounding surface areas were quantified throughout 36 h of cross-circulation using video thermography and image analysis software (ImageJ 1.51m9). Extracted images were subjected to image processing wherein average pixel values were measured in congruent regions of topically cooled areas and areas unaffected by hypothermic cooling. Differences in mean pixel values were calculated and plotted as a function of time.

### Immunohistochemical staining

Following de-paraffinization, lung sections were subjected to boiling citrate buffer (pH 6.0) for antigen retrieval, and blocked with 10% normal goat serum in phosphate-buffered saline for 2 h at room temperature. Next, primary antibodies were diluted 1:100, applied, and incubated for 12 h at 4 °C or 4 h at room temperature. Secondary antibodies were diluted 1:200 and incubated for 1 h at room temperature. Sections were mounted in Vectashield Mounting Medium with DAPI (Vector Laboratories), and coverslips were applied. Images were obtained using an Olympus FSX100 microscope. To detect late apoptosis, a terminal deoxynucleotidyl transferase-mediated deoxyuridine triphosphate nick end-labeling (TUNEL) assay was used. Immunohistochemical stains for aquaporin 5 (Abcam, ab78486), caspase 3 (Abcam, ab13847), CD163 (Abcam, ab87099), CD31 (Abcam, ab28364), connexin 43 (Abcam, ab11370), neutrophil elastase (Abcam, ab68672), surfactant protein C (Abcam, ab196677), zonula occludens-1 (Santa Cruz Biotechnology, sc-10804), and zonula occludens-3 (Abcam, ab205882) were conducted by the Herbert Irving Comprehensive Cancer Center molecular pathology services at Columbia University Medical Center. A list of antibodies and dilutions used is provided in Supplementary Table 10.

### Histopathologic sample collection and analysis

The location of each lung wedge sample was randomized for each time point prior to the start of the experiment. Each lung was divided into 12 regions as shown in Supplementary Fig. 4a. To avoid sampling bias, a random number generator (www.random.org) was used to assign a lung region to each sample collection time point. Tissue sampling was restricted to the lower half of injured and control lungs to eliminate sampling bias in superior segments of upper lobes of injured lungs with better aeration. A surgical stapler with medium/thick reloads was used to obtain lung samples every 6 h. Specimens were immediately fixed in cold phosphate-buffered 4% paraformaldehyde for 48–72 h, embedded in paraffin, and sectioned at 3 μm or 5 μm thickness. All sections were stained for hematoxylin and eosin and examined under light microscopy. Additional sections were stained for Alcian blue (pH 2), trichrome, silver reticulin, elastic van Gieson, and pentachrome by histology services in the Department of Molecular Pathology at Columbia University Medical Center.

### Blinded pathologic review

Blinded pathologic review was performed by a pulmonary pathologist blinded to the study protocol. All slides (H&E, immunohistochemical staining for neutrophil elastase and caspase 3, and TUNEL; Supplementary Fig. 6 a, b; Supplementary Fig. 7f) were randomized, arbitrarily numbered, and delivered to the pathologist without reference to experimental time points or conditions^55^. To evaluate recovery from injury resulting from aspiration of gastric contents, a modified lung injury score was developed from previously described methods^56,57^. Scoring (Supplementary Table 6) was based on number of airway polymorphonuclear cells per high-power field (hpf) (×40; 0.2 mm^2^; 0 ⇒ 0, 1 ⇒ 1–25, 2 ⇒ 26–50, 3 ⇒ >50), number of alveolar polymorphonuclear cells per hpf (×40; 0.2 mm^2^; 0 ⇒ 0, 1 ⇒ 1–25, 2 ⇒ 26–50, 3 ⇒ >50), alveolar edema (0 ⇒ <5% of all alveoli contained edema fluid, 1 ⇒ 6–25%, 2 ⇒ 26–50%, 3 ⇒ >50%), interstitial infiltrate (number of lymphocytes and neutrophils in the interstitium around vessels and airways and in alveolar septa and pleura; 0 ⇒ 0 per hpf, 1 ⇒ <50 per hpf, 2 ⇒ 50–100 per hpf, 3 ⇒ >100 per hpf), interstitial edema (perivascular and peribronchial spaces expanded with edematous fluid; 0 ⇒ 0 per hpf, 1 ⇒ 1x width vessel media, 2 ⇒ ≥2x width vessel media), and total number of apoptotic cells per hpf (×40; 0.2 mm^2^; 0 ⇒ <2, 1 ⇒ 3–5, 2 ⇒ 6–10, 3 ⇒ >10). Numbers for early and late apoptosis, as quantified by caspase 3 and TUNEL, respectively, were combined into a single apoptosis score. Lung injury scores of injured and control lungs were compared at 6 h after gastric aspiration and periodically throughout interventional cross-circulation procedures as an index of lung injury following unilateral delivery of standardized gastric contents.

### Scanning electron microscopy

Lung samples were obtained from multiple time points (0, 18, and 36 h of cross-circulation), and fixed in formalin for 48 h. Specimens were then rinsed in 70% ethanol, frozen, and lyophilized. Samples were imaged using Zeiss GeminiSEM 300 with accelerating voltage of 2.5 kV.

### Transmission electron microscopy

Lung samples were obtained from multiple time points (0, 18, and 36 h of cross-circulation), and fixed with 2.5% glutaraldehyde in 0.1 M Sorensen’s buffer (pH 7.2). Next, lung samples were post-fixed with 1% OsO_4_ in Sorenson’s buffer for 1 h, and following dehydration, tissues were embedded in Lx-112 (Ladd Research Industries). Sectioning was performed on a PT-XL ultramicrotome (60 nm thickness). Samples were then stained with uranyl acetate and lead citrate, and examined with a JEOL JEM-1200 EXII electron microscope. Images capture was performed using an ORCA-HR digital camera (Hamamatsu) and recorded with AMT Image Capture Engine v.602.569.

### Statistical analysis

No data were excluded from analysis. One-way analysis of variance with Tukey’s multiple comparison post hoc tests and Student’s *t*-tests were performed using Prism Version 7.0e (GraphPad). A value of *p* < 0.05 was considered statistically significant.

### Study design

The study was designed as a proof-of-concept study (*n* = 8) for the purposes of testing interventional procedures and demonstrating the ability to support and recover the function of acutely injured lungs with cross-circulation for 36 h, during which standard therapeutic interventions could be performed and the functional recovery of lungs assessed. Our hypothesis was that cross-circulation could provide a sufficiently robust level of extracorporeal support to enable multiple therapeutic interventions and recovery of extracorporeal lung function. The study was conducted with the minimum number of animals needed to demonstrate reproducibility and achieve statistical significance between time points. Data from this initial study will be used to conduct power analysis for subsequent investigations. Collection of all samples was performed as technical replicates in triplicate.

### Randomization of sampling

Samples of extracorporeal lung collected for histologic, microscopic, and pathologic analyses were collected randomly during procedures according to a pre-determined lung map (Supplementary Fig. 4a) with 12 regions arbitrarily numbered for each lung.

### Blinded review

All analytical assessments were blinded to the maximum practical extent. Pathologic analysis was performed by an independent expert to eliminate bias.

### Reporting summary

Further information on research design is available in the Nature Research Reporting Summary linked to this article.

## Supplementary information

Supplementary Information

Description of Additional Supplementary Files

Supplementary Movie 1

Supplementary Movie 2

Supplementary Movie 3

Supplementary Movie 4

Supplementary Movie 5

Supplementary Movie 6

Supplementary Movie 7

Supplementary Movie 8

Reporting Summary

Source Data

## Data Availability

The authors declare that all data reported in the paper and Supplementary Information will be made available to other investigators if requested. The source data underlying Fig. 2c–i, n; Fig. 3c–e, l; Fig. 4c; Fig. 5d; Fig. 6d–f,o are provided in the Source Data file.
