# Supplementary material for: Regeneration of severely damaged lungs using an interventional cross-circulation platform
Source: Nat Commun. 2019 May 7;10:1985. doi: 10.1038/s41467-019-09908-1 (PMC6504972; doi:10.1038/s41467-019-09908-1)
Supplement: Supplementary file 1 — Supplementary Information [file 41467_2019_9908_MOESM1_ESM.pdf]

Regeneration of severely damaged lungs using an interventional cross-circulation platform

Brandon A. Guenthart[†,1,2], John D. O'Neill[†,1], Jinho Kim[1,3], Dawn Queen[1], Scott Chicotka[2], Kenmond Fung[4], Michael Simpson[2], Rachel Donocoff[5], Michael Salna[2], Charles C. Marboe[6], Katherine Cunningham[1], Susan P. Halligan[1], Holly M. Wobma[1], Ahmed E. Hozain[1,2], Alexander Romanov[5], Gordana Vunjak-Novakovic[1,7,*], Matthew Bacchetta[1,8*]

[1]Department of Biomedical Engineering, Columbia University Medical Center, Columbia University, New York, 10032 NY, USA. [2]Department of Surgery, Columbia University Medical Center, Columbia University, New York, 10032 NY, USA. [3]Department of Biomedical Engineering, Stevens Institute of Technology, Hoboken, 07030 NJ, USA. [4]Department of Clinical Perfusion, Columbia University Medical Center, Columbia University, New York, 10032 NY, USA. [5]Institute of Comparative Medicine, Columbia University Medical Center, Columbia University, New York, 10032 NY, USA. [6]Department of Pathology and Cell Biology, Columbia University Medical Center, Columbia University, New York, 10032 NY, USA. [7]Department of Medicine, Columbia University Medical Center, Columbia University, New York, 10032, NY, USA. [8]Department of Thoracic and Cardiovascular Surgery, Vanderbilt University, Nashville, 37232 TN, USA. [†]Authors contributed equally. *Corresponding authors: gv2131@cvolumbia.edu and matthew.bacchetta@vumc.org

SUPPLEMENTARY INFORMATION

TABLE OF CONTENTS

Supplementary Discussion 3
Introduction …… 3
In vivo response to gastric aspiration and validation of lung injury …… 3
Tissue sampling …… 5
Therapeutic interventions …… 5
Hemodynamic stability of recipients during interventional cross-circulation …… 5
Lung recovery and regeneration …… 6
Advanced lung diagnostics …… 6
Envisioned clinical application of cross-circulation …… 7
References …… 9
Supplementary Figure 1: Validation and characterization of gastric aspiration injury …… 12
Supplementary Figure 2: Cannulation and single lung vascular and airway analytics …… 13
Supplementary Figure 3: Experimental setup …… 14
Supplementary Figure 4: Randomized lung sampling and analyses of BAL fluid …… 15
Supplementary Figure 5: Gross, radiographic, & pathologic analyses of lung recovery .. 16
Supplementary Figure 6: Pathologic immunohistochemical staining, injury scoring …… 17
Supplementary Figure 7: Histological analysis throughout recovery …… 18
Supplementary Figure 8: Lung thermography and surfactant delivery …… 19
Supplementary Figure 9: Envisioned clinical application of cross-circulation …… 20
Supplementary Table 1: Description of studies investigating the use of cross-circulation or *ex vivo* lung perfusion (EVLP) in swine models of aspiration injury …… 21
Supplementary Table 2: Comparison of lung function data reported in studies investigating the use of cross-circulation or *ex vivo* lung perfusion (EVLP) in swine models of aspiration injury …… 22
Supplementary Table 3: Comparison of bronchoalveolar lavage (BAL) fluid and perfusate data reported in studies investigating the use of cross-circulation or *ex vivo* lung perfusion (EVLP) in swine models of aspiration injury …… 23
Supplementary Table 4: Comparison of scope of analysis and data reported in studies investigating the use of cross-circulation or *ex vivo* lung perfusion (EVLP) in swine models of aspiration injury …… 25
Supplementary Table 5: Donor response to gastric aspiration …… 26
Supplementary Table 6: Scoring rubric of lung injury score …… 27
Supplementary Table 7: Recipient safety data …… 28
Supplementary Table 8: Inflammatory cytokines in recipient serum …… 29
Supplementary Table 9: Inflammatory cytokines in BAL fluid …… 30
Supplementary Table 10: Primary and secondary antibodies …… 31
Supplementary Table 11: ELISA product kits …… 32
Supplementary Movies 1–8 …… 33

SUPPLEMENTARY DISCUSSION

***Introduction* –** Current approaches to whole organ culture and *ex vivo* organ perfusion provide limited durations of organ support, as neither technique can prevent the series of adverse events (e.g., vascular permeability, pulmonary edema, cell death) that ultimately lead to organ failure. Successful long-term extracorporeal organ support, measured in days, has so far only been achieved when organs are supported by the blood or plasma of a living host[1, 2]. It has been proposed that the lack of essential hormonal regulation following brain death in isolated *ex vivo* perfusion systems is deleterious and partially responsible for organ failure[3]. Similarly, we contend that *ex vivo* perfusion devices lacking systemic homeostatic regulation and intrinsic factors present in plasma or whole blood are incapable of providing the duration and quality of support required for: (*i*) the recovery and regeneration of severely damaged organs, or (*ii*) implementation of advanced therapeutics (e.g., cell replacement[4, 5, 6]) for whole organ repair.

While *ex vivo* lung perfusion (EVLP) has been shown to be effective for donor organ assessment and research, previous attempts at reconditioning severely damaged lungs utilizing conventional EVLP lack compelling evidence that amelioration of the inflammatory response, tissue recovery and cellular regeneration are feasible[7, 8, 9, 10, 11, 12] (**Supplementary Table 1-3**). Acknowledgement of this intrinsic limitation is critical, as lungs remain the least utilized organ for transplantation, with a ratio of 32:1 unused lungs to waiting list patients removed due to progression of illness or death[13]. The reasons that isolated EVLP systems have not been widely adopted or made a significant impact on donor pool expansion may be multifactorial and include technical limitations (e.g., duration and quality of support), clinical availability (e.g., high costs of devices and disposable components, staff, training), and ill-defined indications for use. These limitations highlight the need to develop organ support and recovery systems[14] with regenerative capabilities that enable the recovery of severely damaged organs beyond those deemed 'marginal'.

Additionally, while previous studies utilizing EVLP platforms have investigated recovery of global lung function (e.g., compliance, gas exchange), little has been reported about the dynamic interplay between cellular activity and regeneration of lungs maintained on extracorporeal support (**Supplementary Table 4**). Here, we describe the development and application of an interventional cross-circulation platform in the context of a gastric aspiration injury model in a large animal, with the use of single lung cannulation and intraprocedural assessment techniques, and the implementation of a clinically relevant therapeutic regimen. After 36 hours of interventional cross-circulation, injured lungs demonstrated a significant degree of recovery across molecular, cellular, and tissue milieus.

***In vivo response to gastric aspiration and validation of lung injury* –** Lung injury was induced *in vivo* by aspiration of gastric contents in a swine model according to previously established protocols[8, 15]. Standardized gastric contents contained macroscopic and microscopic particulate matter, pepsin, and bile acids (*see Methods: Gastric aspiration injury model*) to ensure a severe and reproducible lung injury, as gastric aspirate composition (total volume, pH, acidified and non-acidified particulates, enzymes including pepsin, and bile acids) is correlated with injury severity.

In addition to continuous hemodynamic monitoring, lung injury progression was assessed every 2 hours by radiography, bronchoscopy, bronchoalveolar lavage fluid analysis, and serologic and hemogas analyses. Prior to bronchoscopic delivery of gastric contents, baseline chest X-rays (**Fig. 2a**) and airway bronchoscopy (**Fig. 2b**) appeared normal. Video bronchoscopy was used to confirm unilateral delivery of gastric contents into lungs (**Fig. 2b; Supplementary Movie 2**). Six hours after *in vivo* delivery of gastric contents, chest X-rays showed marked unilateral lung injury characterized by increased radiopacity, basilar atelectasis, and areas of consolidation with air

bronchograms (**Fig. 2a**). Bronchoscopy revealed severe unilateral airway inflammation, edema, and mucus secretions (**Fig. 2b**) resulting from the insult of gastric contents.

The systemic response of the lung donor to gastric aspiration was significant: elevated heart rate (baseline, 85 ± 11 bpm; 6 h, 130 ± 7 bpm; **Fig. 2c**), decreased mean arterial pressure (MAP: baseline, 99 ± 6 mmHg; 6 h, 60 ± 8 mmHg; **Fig. 2d**), and increased neutrophil count (baseline, 38 ± 9 %WBC; 6 h, 61 ± 16 %WBC; **Fig. 2e**). Hemogas analyses confirmed reduced oxygenation (pO_2: baseline, 534 ± 74 mmHg; 6 h, 336 ± 39 mmHg; **Fig. 2f**), hypercapnia (pCO_2: baseline, 39 ± 3 mmHg; 6 h, 49 ± 5 mmHg; **Fig. 2g**), and significantly decreased gas exchange capacity (PaO_2/FiO_2: baseline, 534 ± 74 mmHg; 6 h, 336 ± 39 mmHg; $p < 0.05$; **Fig. 2h**). Significant reductions in PaO_2/FiO_2, MAP, and cardiac output correlated with the severity of lung injury and were consistent with previously described derangements following aspiration of gastric contents in similar models[16]. In response to injury, significant increases were measured in serum levels of IFNγ, IL-1rα, IL-6, P-selectin, TNFα, and free hemoglobin 6 hours after aspiration of gastric contents ($p < 0.001$). Serum levels of GM-CSF, IL-1α, IL-8, IL-10 and hemolytic markers D-Dimer and fibrinogen also increased (**Supplementary Table 5**).

The pulmonary airway response to aspiration of gastric contents was assessed by quantification of inflammatory cytokines in bronchoalveolar lavage (BAL) fluid and staining of BAL fluid smears. Levels of inflammatory cytokines IFNγ, IL-1α, IL-1β, IL-1rα, IL-2, IL-4, IL-6, IL-8, IL-10, IL-12, IL-18, and TNFα in BAL fluid were significantly elevated 6 hours after gastric aspiration ($p < 0.001$; **Fig. 2i, Supplementary Table 5**). Significantly more inflammatory cells, including lymphocytes, polymorphonuclear cells, and mast cells, were observed in BAL fluid from injured lungs compared to BAL fluid from control lungs, which appeared normal and showed no signs of airway injury or disruption (**Fig. 2j; Supplementary Figure 1j**). Lung donors experienced hemodynamic instability (increased heart rate, decreased MAP) and developed a severe inflammatory response (significant elevation in levels of IFNγ, IL-1α, IL-1β, IL-6, IL-8, TNFα in BAL fluid), consistent with previously described effects of large volume aspiration[16] (**Fig. 2, Supplementary Table 5**).

The cellular response to gastric aspiration was assessed by histology and immunohistochemical staining. Hematoxylin & eosin staining of injured lungs 6 hours after aspiration of gastric contents confirmed severe lung injury: dramatic inflammatory response, significant cellular derangement, and peribronchial edema (**Fig. 2k; Supplementary Figure 1e-h**), with localized regions of alveolar hemorrhage, alveolar edema, and pervasive infiltration of leukocytes. Pentachrome staining showed perivascular edema, vascular intimal thickening, and extravasation of inflammatory cells in injured lungs (**Fig. 2l**). Immunostaining for caspase 3, a marker of early apoptosis, showed numerous apoptotic cells throughout injured lungs 6 hours after gastric aspiration, while apoptotic cells were very rarely observed in control lungs (**Fig. 2m**). Pathologic assessment of lungs was performed in a randomized and blinded fashion according to a previously established protocol[1, 17, 18] to obtain lung injury scores of injured and control lungs at baseline and 6 hours after gastric aspiration. Lungs were scored by quantification of airway and alveolar polymorphonuclear cells, alveolar and interstitial edema, interstitial infiltrate, and early and late apoptotic cells (**Supplementary Table 6**). Injured lungs received significantly higher injury scores than control lungs across all injury categories (**Fig. 2n; Supplementary Figure 1k**).

Lungs were procured from the donor in standard fashion[19, 20] six hours after gastric aspiration and cannulated on ice using a bio-bridge and crenellated drainage cannula to manage pulmonary venous outflow (**Supplementary Figure 2a-c**) as previously described[1]. Additional cannulas were placed into the main pulmonary veins of the lower lobes of injured and control lungs (i.e., single lung venous cannulation; **Fig. 1c-g; Supplementary Figure 2d-h**) to facilitate analysis of gas exchange in injured and control lungs separately.

Tissue sampling – The gross appearance of lungs upon explant revealed extensive lung consolidation and pulmonary edema, and severe inflammation (**Supplementary Figure 1e**). As is commonly observed clinically and attributable to gravitational forces and positive pressure ventilation[21], dependent lung regions (lower lobes and posterior segments of upper lobes) were frequently more consolidated, less likely to ventilate, and thus more difficult to recruit. Consequently, randomized tissue sampling (**Supplementary Figure 4a**) and surface thermography diagnostics (**Fig. 5 d-g; Supplementary Figure 8a-f**) were restricted to lower lobes of injured and control lungs to eliminate sampling bias in superior segments of upper lobes with better aeration (*see Methods: Histopathologic analysis*).

Therapeutic interventions – After cross-circulation was established, extracorporeal lungs were continually assessed and subjected to airway lavage (**Supplementary Movie 4**), surfactant replacement (**Supplementary Movie 5**), and alveolar recruitment maneuvers (**Supplementary Movie 6**). The therapeutic interventions that were systematically performed on injured extracorporeal lungs represent standard clinical interventions and were shown to promote statistically significant recovery of injured lungs on extracorporeal support (**Fig. 3-6; Supplementary Figure 4-8**).

The rationale for including surfactant replacement in the therapeutic regimen was twofold: (*i*) While bronchoalveolar lavage can be effective in removing particulate debris and neutralizing the pH of acidic gastric contents in the lungs, repeated airway lavage has been shown to deplete endogenous surfactant and impact the blood–gas barrier[22]. (*ii*) Furthermore, decreased endogenous synthesis and secretion of surfactants as well as alteration and degradation of surfactant phospholipids by alveolar macrophages is well described in the early stages of acute lung injury[23, 24, 25, 26]. Following repeated lavage, pepsin concentration in BAL fluid decreased throughout interventional cross-circulation. Although the pepsin concentration in BAL fluid from injured lungs ranged from 1.8 – 61.7 ng mL^{-1} after 12 hours of cross-circulation, the proteolytic activity of any residual pepsin[27] would have been significantly reduced at the normalized pH of 5.5 measured at and maintained after 12 hours of cross-circulation. Microbial cultures of BAL fluid from injured and control lungs were negative for pathologic growth in all cases. This result demonstrates that (*i*) injured and control lungs did not develop infection, and (*ii*) repeated organ manipulations including bronchoscopy, diagnostic imaging, and tissue sampling can be performed without introducing pathogens into extracorporeal lungs.

Hemodynamic stability of recipients during interventional cross-circulation – Median weight of recipient animals (n = 8) was 39.2 kg (range: 30.6 – 53.5 kg). All recipients remained hemodynamically stable throughout the duration of cross-circulation support, in normal sinus rhythm, and without the need for vasopressor support. Notably, there were no significant differences between baseline and endpoint values in lactate (1.86 ± 0.41 mmol L^{-1} and 1.04 ± 0.05 mmol L^{-1}), pH (7.45 ± 0.01 and 7.38 ± 0.02), or pO_2 (500 ± 94 mmHg and 514 ± 65 mmHg), respectively. Hemolytic markers (LDH, D-dimer, fibrinogen, plasma free hemoglobin) remained within normal ranges (**Supplementary Table 7**).

No statistically significant changes in serum levels of inflammatory cytokines were detected over 36 hours of cross-circulation for GM-CSF, IFNγ, IL-1α, IL-1β, IL-1rα, IL-2, IL-4, IL-6, IL-8, IL-10, IL-12, IL-18, and TNFα (**Supplementary Table 8**). P-selectin, an indicator of platelet activation and endothelial injury, was elevated at baseline (0 h, 10.4 ± 0.6 ng mL^{-1}) but did not increase significantly and remained within normal range throughout the entire procedure (36 h, 11.7 ± 0.7 ng mL^{-1}; **Supplementary Table 8**). Notably, levels of IFNγ, IL-1rα, IL-2, IL-6, IL-10, IL-18, and TNFα increased over the first 12 hours of cross-circulation before trending downward between 12 – 36 hours of cross-circulation. All inflammatory cytokine levels in recipient serum were within previously reported normal ranges[1] (**Supplementary Table 7, 8**).

Lung recovery and regeneration – The recovery of global lung function can be attributed to many reparative mechanisms that can actively restore tissue integrity. Multi-scale analyses including global lung imaging (**Supplementary Figure 5a-c**), airway bronchoscopy (**Fig. 5k**), and histology and electron microscopy (**Fig. 5, 6; Supplementary Figure 5, 6, 7**) indicated a significant degree of recovery. After 36 hours of interventional cross-circulation, histologic analyses showed amelioration of lung injury with significantly reduced inflammation and clearance of alveolar fluid. Bronchioles and alveolar ducts were well-aerated, free of fluid and debris, with minimal interstitial thickening and clustering of neutrophils along the alveolar surface (**Fig. 5h**). Electron microscopy revealed characteristic saccular alveolar architecture (**Fig. 5i**), reduction of edematous alveolar septa, and restoration of the alveolar-capillary membrane (**Fig. 5j, Supplementary Figure 7b**). Altogether, multi-scale analyses of injured lungs by light and electron microscopy and pathologic lung injury scoring (**Fig. 5l; Supplementary Figure 5g-i; Supplementary Figure 6**) indicated significant resolution of the inflammatory process and suggested a notable degree of recovery.

Type II pneumocytes in injured lungs contained little or no surfactant stores at initiation (0 hours) of cross-circulation (**Fig. 6k**). However, by 36 hours of cross-circulation, abundant stores of surfactant, consistent with immunofluorescence staining of SPC (**Fig. 6k; Supplementary Figure 7e**), were observed in type II cells, with evidence of surfactant secretion onto the alveolar surface (**Fig. 6l; Supplementary Figure 7e**). Viability and function of type II pneumocytes were confirmed by live uptake of fluorescently labeled surfactant (BODIPY-SPB, **Fig. 6m**) following 36 hours of interventional cross-circulation.

Connexin 43-mediated gap junctions are known to play a role as conduits for the distribution of inflammatory mediators[28]. In this study, we observed notable increases in expression of connexin 43 in severely injured lungs (**Fig. 6o**), which correlated with lung recovery over 36 hours of support. These results may suggest a possible protective or reparative mechanism involving intercellular communications via the upregulation of connexin 43 following gastric aspiration injury. Pulmonary endothelial viability was confirmed at 36 hours by fluorescence microscopy of receptor-mediated uptake of acetylated low-density lipoprotein (LDL) and intracellular cleavage of carboxyfluorescein succinimidyl ester (CFSE, **Fig. 6s,t**). Immunostaining of endothelial marker CD31 and microvascular tight junction protein ZO-1 and transmission electron microscopy revealing intact pulmonary capillaries and absence of extravascular erythrocytes (**Fig. 6q-r; Supplementary Figure 7b**) altogether provide strong evidence that in severely damaged lungs the pulmonary vascular network not only remained viable but also substantially recovered and regenerated over 36 hours of interventional cross-circulation.

Advanced lung diagnostics – Thermal imaging has developed considerably since it first became available for non-military applications in the 1950s[29]. Driven by various industrial applications, infrared imaging technology has become more affordable, reliable, and portable, and computerization has made clinical translation feasible[30, 31]. Due to the unique thermodynamic properties of the lung and enhanced thermal imaging resolution of extracorporeal lungs without interference or loss of signal through the chest wall and thoracic cavity, thermography holds great promise as a diagnostic tool to aid in extracorporeal organ assessment and recovery.

In this study, average surface temperatures of injured lungs were initially elevated relative to average surface temperatures of control lungs (6 h, injured: 33.5 ± 0.5 ºC; control: 31.2 ± 0.4 ºC), but gradually decreased and approached those of control lungs, which remained constant at 31.3 ± 0.3 ºC (**Fig. 5d**). Consistent with these results, previous studies have shown that lungs function as a steady-state heat exchanger, with an internal heat source (pulmonary blood flow) and external heat sink (ventilation)[32]. As a result, during ventilation a temperature gradient develops

between the blood and lung tissue. During cross-circulation, the temperature gradient (ΔT) between average blood temperature (34.5 ± 0.7 ºC) and average surface temperatures of control lungs was constantly maintained at ΔT = 3.1 ± 0.3 ºC due to steady state heat exchange in control lungs. Notably, however, the temperature gradient between average blood temperature and the average surface temperatures of injured lungs steadily increased from ΔT = 1.0 ± 0.3 ºC to ΔT = 2.1 ± 0.5 ºC, approaching the temperature differential maintained throughout 36 hours of cross-circulation between the blood and the pleura in control lungs (**Supplementary Figure 8a,b**).

To assess the degree and amelioration of localized lung injury over time, thermographic assessments were conducted locally at the surfaces of injured and control lungs throughout 36 hours of cross-circulation. Prior to each assessment, baseline thermographs of the dorsal aspect of injured and control lungs were obtained. Then, transient conduction resulting from thermal contact between a blunt tip hypothermic probe maintained at 4ºC and the surface of injured or control lungs (contact pressure: 1.0 N; duration: 2 s) was assessed by infrared videography (**Fig. 5f**; **Supplementary Figure 8c, Supplementary Movie 7**). Average temperature differences between topically cooled regions of lungs and corresponding regions of surrounding lung parenchyma were extrapolated from thermal videos taken during the rewarming process, and determined to decay over time. Curves fitted ($R^2 \geq 0.92$) to calculated temperature differences over time decayed exponentially, and characteristic time constants were determined for injured (**Fig. 5e**) and control (**Supplementary Figure 8f**) lungs throughout 36 hours of cross-circulation.

Exosomes – The abundance of exosomes in biofluids (e.g., blood, urine) has sparked interest in their use as a biomarker of various diseases [33]. Here, we evaluated exosomes in BAL fluid from injured and control lungs (**Fig. 3g; Supplementary Figure 4d-f**) and observed a significant increase in exosome concentration that trended downward over time as injured lungs recovered, approaching exosome concentration in BAL fluid from control lungs. To our knowledge, this finding may represent the first report of the number of exosomes in BAL fluid as a potential biomarker for acute lung injury.

Envisioned clinical application of cross-circulation – Extracorporeal lungs have the distinct advantage of direct and continuous accessibility. This setting allows for gross observations not possible following transplantation and better facilitates organ manipulations (e.g., recruitment maneuvers), diagnostic assessment and imaging (e.g., hemogas analysis, repeated bronchoscopy, radiography, thermography, advanced transpleural imaging), and interventions (e.g., airway lavage, biopsy, targeted drug or cell delivery, cell replacement).

One major advantage of extracorporeal organ support by cross-circulation over proceeding directly to transplantation with high risk lungs is that cross-circulation enables therapeutic interventions without the concurrent stress of transplantation surgery. Following transplantation, any graft dysfunction would preclude therapeutic intervention because of the recipient’s vital need for gas exchange. Cross-circulation can allow for safe manipulation of donor lungs without compromising the respiratory status of the recipient.

In this study, which utilized a healthy swine host, an oxygenator was not required and was therefore not used. Venous blood from the host supplied to the extracorporeal lungs was sufficient to support the low metabolic demand of extracorporeal lungs. An oxygenator, however, could easily be added to the cross-circulation circuit if necessitated by the extracorporeal lungs or host. For example, in the envisioned clinical application of cross-circulation (**Supplementary Figure 9**), a human recipient awaiting lung transplantation could already be on extracorporeal membrane oxygenation (ECMO) support and reliant on the oxygenator in the ECMO circuit.

We envision that initial candidates for the clinical application of cross-circulation may be young patients with isolated lung disease on extracorporeal membrane oxygenation (ECMO) support as a bridge-to-transplant (**Supplementary Figure 9**). Every day transplant surgeons decline lungs that are too damaged to be accepted for transplantation. While severely damaged lungs are constantly discarded, patients on the waiting list die or become too ill to remain listed. We maintain that cross-circulation could enable the recovery of previously unsalvageable donor lungs and offer a subset of patients the opportunity to receive an organ that they may otherwise never receive.

References (Supplementary Text)

1. O'Neill JD*, et al.* Cross-circulation for extracorporeal support and recovery of the lung. *Nature Biomedical Engineering* **1**, 0037 (2017).

2. Lavender A, Forland M, Rams JJ, Thompson JS, Russe HP, Spargo BH. Extracorporeal renal transplantation in man. *Jama* **203**, 265-271 (1968).

3. Bartlett RH. Vitalin: The rationale for a hypothetical hormone. *Journal of the American College of Surgeons* **199**, 286-292 (2004).

4. Wagner DE*, et al.* Comparative decellularization and recellularization of normal versus emphysematous human lungs. *Biomaterials* **35**, 3281-3297 (2014).

5. Hogan BL*, et al.* Repair and regeneration of the respiratory system: complexity, plasticity, and mechanisms of lung stem cell function. *Cell stem cell* **15**, 123-138 (2014).

6. Guenthart BA, O'Neill JD, Kim J, Fung K, Vunjak-Novakovic G, Bacchetta M. Cell replacement in human lung bioengineering. *The Journal of Heart and Lung Transplantation* **38**, 215-224 (2019).

7. Khalifé-Hocquemiller T*, et al.* Exogenous surfactant attenuates lung injury from gastric-acid aspiration during ex vivo reconditioning in pigs. *Transplantation* **97**, 413-418 (2014).

8. Meers CM*, et al.* A model of ex vivo perfusion of porcine donor lungs injured by gastric aspiration: a step towards pretransplant reconditioning. *Journal of Surgical Research* **170**, e159-e167 (2011).

9. Inci I, Hillinger S, Arni S, Kaplan T, Inci D, Weder W. Reconditioning of an injured lung graft with intrabronchial surfactant instillation in an ex vivo lung perfusion system followed by transplantation. *journal of surgical research* **184**, 1143-1149 (2013).

10. Khalifé-Hocquemiller T, Sage E, Dorfmuller P, Eddahibi S, Fadel E. 394 Ex Vivo Perfusion Worsened Lung Injuries Induced by Gastric Acid Aspiration in Pigs. *The Journal of Heart and Lung Transplantation* **30**, S135-S136 (2011).

11. Meers CM*, et al.* Preemptive therapy with steroids but not macrolides improves gas exchange in caustic-injured donor lungs. *Journal of Surgical Research* **170**, e141-e148 (2011).

12. Nakajima D*, et al.* Lung Lavage and Surfactant Replacement During Ex Vivo Lung Perfusion for Treatment of Gastric Acid Aspiration–Induced Donor Lung Injury. *The Journal of Heart and Lung Transplantation* **36**, 577-585 (2017).

13. Giwa S*, et al.* The promise of organ and tissue preservation to transform medicine. *Nature Biotechnology* **35**, 530-542 (2017).

14. Abrams D, Bacchetta M, Brodie D. When the momentum has gone: what will be the role of extracorporeal lung support in the future? *Current opinion in critical care* **24**, 23-28 (2018).

15. Meers CM*, et al.* A porcine model of acute lung injury by instillation of gastric fluid. *Journal of Surgical Research* **166**, e195-e204 (2011).

16. Fraisse A*, et al.* Hemodynamics in experimental gastric juice induced aspiration pneumonitis. *Intensive care medicine* **33**, 300-307 (2007).

17. Reece TB*, et al.* Adenosine A 2A receptor activation reduces inflammation and preserves pulmonary function in an in vivo model of lung transplantation. *The Journal of thoracic and cardiovascular surgery* **129**, 1137-1143 (2005).

18. Mulloy DP*, et al.* Ex vivo rehabilitation of non–heart-beating donor lungs in preclinical porcine model: Delayed perfusion results in superior lung function. *The Journal of thoracic and cardiovascular surgery* **144**, 1208-1216 (2012).

19. Saxena P, Zimmet AD, Snell G, Levvey B, Marasco SF, McGiffin DC. Procurement of lungs for transplantation following donation after circulatory death: the Alfred technique. *Journal of Surgical Research* **192**, 642-646 (2014).

20. Sundaresan S, Trachiotis GD, Aoe M, Patterson GA, Cooper JD. Donor lung procurement: assessment and operative technique. *The Annals of thoracic surgery* **56**, 1409-1413 (1993).

21. Franquet T, Giménez A, Rosón N, Torrubia S, Sabaté JM, Pérez C. Aspiration diseases: findings, pitfalls, and differential diagnosis. *Radiographics* **20**, 673-685 (2000).

22. Lachmann B, Robertson B, Vogel J. In vivo lung lavage as an experimental model of the respiratory distress syndrome. *Acta anaesthesiologica Scandinavica* **24**, 231-236 (1980).

23. Long DL*, et al.* Secretory phospholipase A2-mediated depletion of phosphatidylglycerol in early acute respiratory distress syndrome. *The American journal of the medical sciences* **343**, 446-451 (2012).

24. Lopez-Rodriguez E, Gay-Jordi G, Mucci A, Lachmann N, Serrano-Mollar A. Lung surfactant metabolism: early in life, early in disease and target in cell therapy. *Cell and tissue research* **367**, 721-735 (2017).

25. Chabot S*, et al.* Inhibitory effects of surfactant protein A on surfactant phospholipid hydrolysis by secreted phospholipases A2. *The Journal of Immunology* **171**, 995-1000 (2003).

26. Ingenito EP*, et al.* Decreased surfactant protein-B expression and surfactant dysfunction in a murine model of acute lung injury. *American journal of respiratory cell and molecular biology* **25**, 35-44 (2001).

27. Johnston N, Dettmar PW, Bishwokarma B, Lively MO, Koufman JA. Activity/stability of human pepsin: implications for reflux attributed laryngeal disease. *The Laryngoscope* **117**, 1036-1039 (2007).

28. Parthasarathi K*, et al.* Connexin 43 mediates spread of Ca 2+-dependent proinflammatory responses in lung capillaries. *The Journal of clinical investigation* **116**, 2193-2200 (2006).

29. Mikulska D. Contemporary applications of infrared imaging in medical diagnostics. In: *Annales Academiae Medicae Stetinensis* (ed^(eds) (2006).

30. Ring E, Ammer K. Infrared thermal imaging in medicine. *Physiological measurement* **33**, R33 (2012).

31. Wang LT*, et al.* Similarity of chest X-ray and thermal imaging of focal pneumonia: a randomised proof of concept study at a large urban teaching hospital. *BMJ open* **8**, e017964 (2018).

32. Serikov V, Rumm M, Kambara K, Bootomo M, Osmack A, Staub N. Application of respiratory heat exchange for the measurement of lung water. *Journal of applied physiology* **72**, 944-953 (1992).

33. Poudineh M, Sargent EH, Pantel K, Kelley SO. Profiling circulating tumour cells and other biomarkers of invasive cancers. *Nature Biomedical Engineering* **2**, 72 (2018).

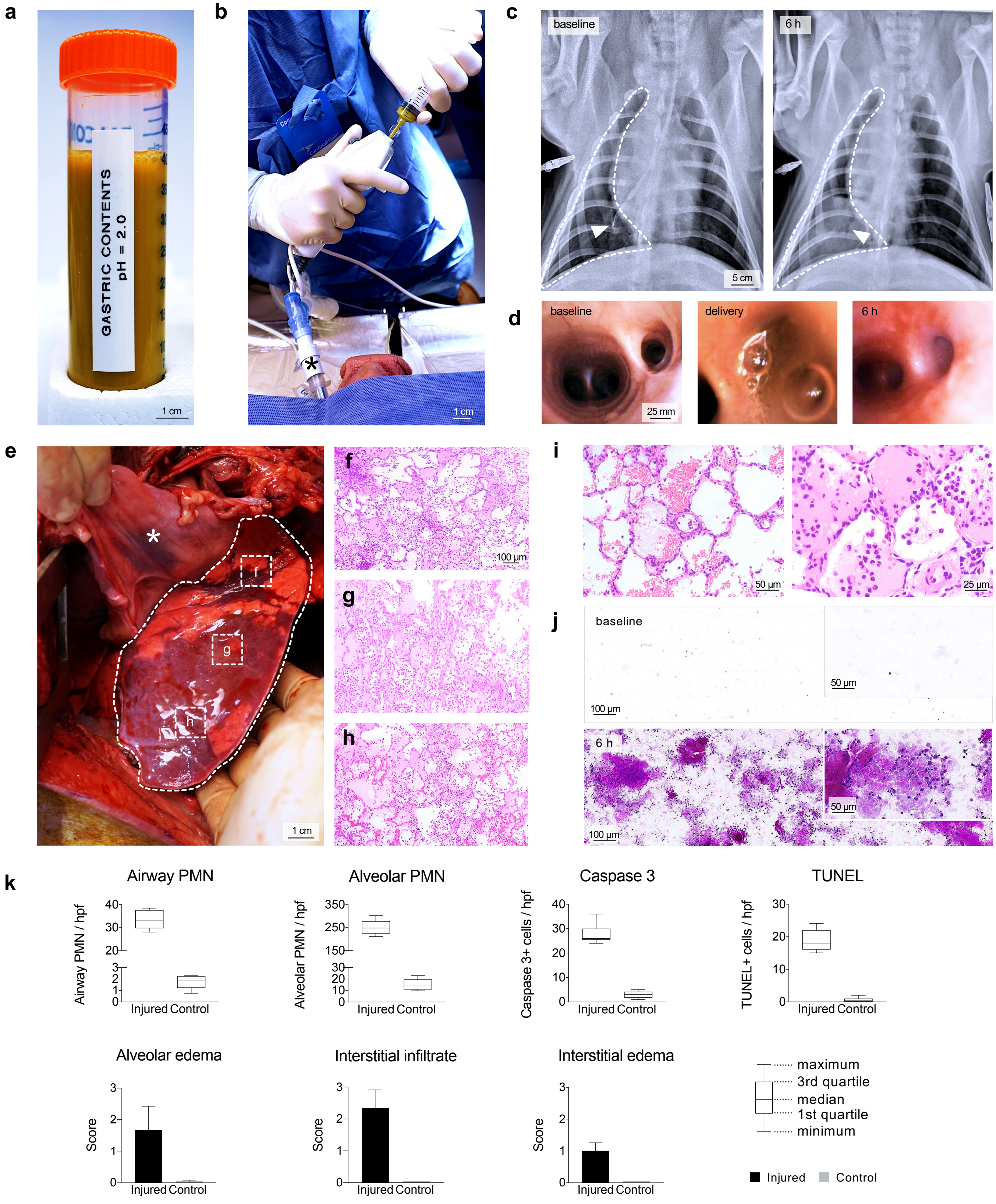

Supplementary Figure 1 | Validation and characterization of gastric aspiration injury. (**a**) Gastric contents collected from multiple donors (*n* = 6), admixed, aliquotted, and standardized to pH 2. (**b**) Bronchoscopic delivery of gastric contents into single lung of anesthetized donor swine. Bronchoscope inserted through endotracheal tube (star). (**c**) Chest X-ray of donor swine at baseline and 6 hours after delivery of gastric contents. Arrow at baseline indicates bronchoscope tip in the left lung prior to delivery, while arrow at 6 h marks an air bronchogram. (**d**) Bronchoscopic analysis of donor pulmonary airways at baseline, during delivery of gastric contents, and 6 hours after delivery of gastric contents. (**e**) Injured donor lung exposed via midline sternotomy in living donor swine 6 hours after delivery of gastric contents, pericardium (star) retracted to enable visualization of injured lung in donor swine. (**f-h**) H&E staining of upper, middle, and lower regions of injured lung demonstrating global lung injury with alveolar edema and hemorrhage. (**i**) High magnification of hemorrhagic injury, and edema with neutrophilic infiltration. (**j**) Periodic acid-Schiff staining of BAL fluid smears prepared from samples collected at baseline and 6 hours after delivery of gastric contents. (**k**) Box-and-whisker plot cell counts and pathologic scoring of donor lungs (*n* = 8, injured and control) 6 hours after delivery of gastric contents.

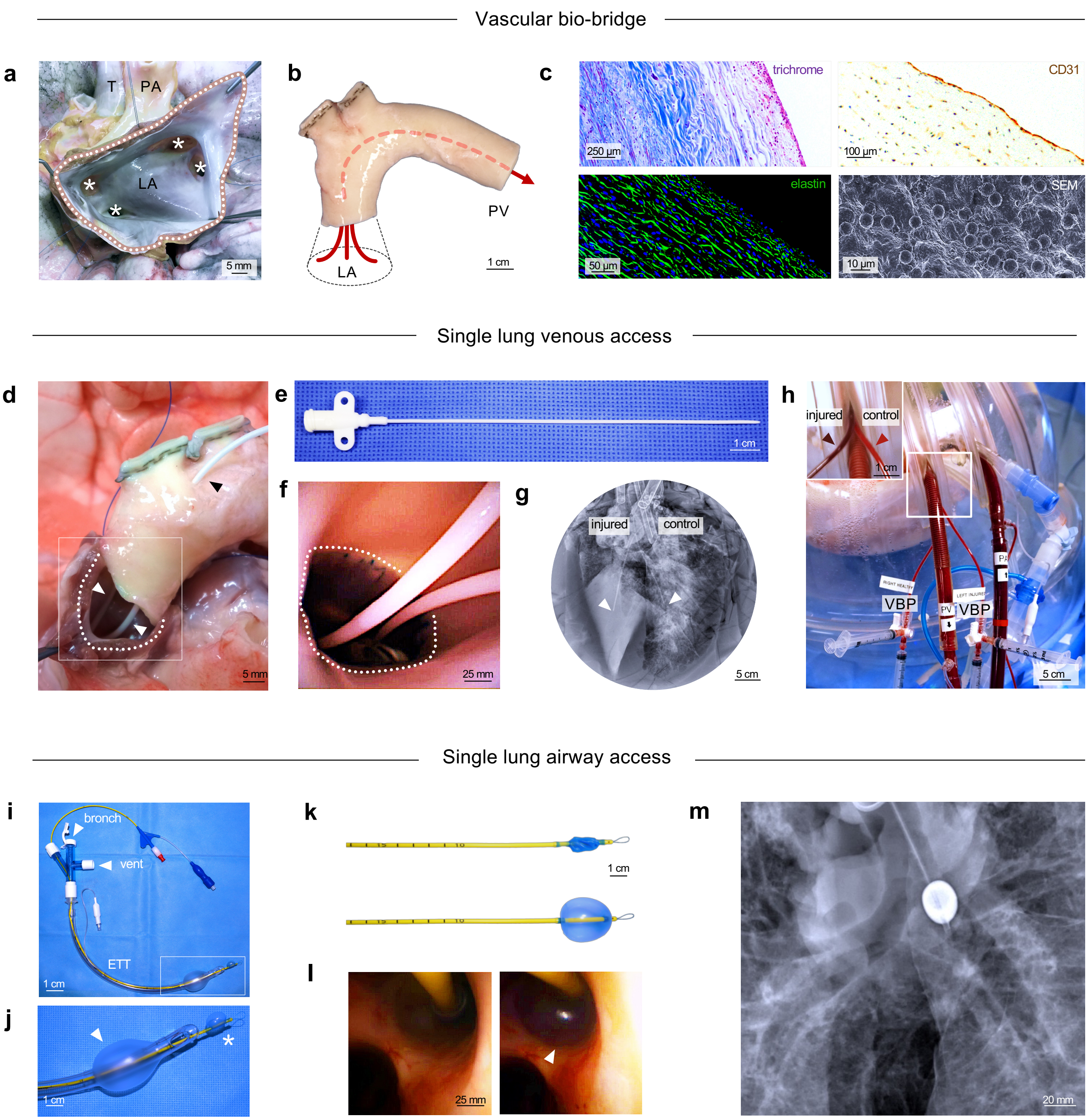

Supplementary Figure 2 | Cannulation and single lung vascular and airway analytics. (**a**) Left atrial (LA) cuff (dotted line) following removal of the heart demonstrating orifices of pulmonary veins (stars). T: trachea; PA: pulmonary artery. (**b**) Bio-bridge prepared from donor aortic arch with brachiocephalic and left subclavian branches stapled closed. Inflow from pulmonary venous drainage via the LA cuff, outflow via pulmonary venous (PV) drainage cannula. (**c**) Microscopic analyses of bio-bridge after 36 hours of cross-circulation: trichrome staining demonstrating collagenous composition, elastin immunostaining demonstrating elastic fibers in bio-bridge wall, CD31 immunostaining confirming intact endothelial lining of bio-bridge, scanning electron microscopy showing red blood cells on luminal surface of bio-bridge with notable absence of platelet aggregation or surface disruption. (**d**) Single lung venous access via bio-bridge. Bio-bridge partially sutured to LA cuff with single lung pulmonary venous blood sampling cannulae (white arrows) placed through wall of bio-bridge (black arrow). (**e**) Single lung venous blood sample cannula. (**f**) *In situ* fiber optic image of single lung venous cannulae in the bio-bridge (dotted line indicates anastomosis between LA cuff and bio-bridge). (**g**) X-ray demonstrating positions of single lung venous blood sampling cannulae in injured and control lungs. (**h**) Venous blood ports (VBP) used to sample blood from injured or control lungs. Bronchial blocker setup: (**i**) insertion through endotracheal tube (ETT) with ports for video bronchoscope, air ventilation, and bronchial blocker. (**j**) Distal tip of ETT with inflated tracheal cuff (arrow) and bronchial blocker exiting ETT, with bronchial blocker balloon inflated (star). (**k**) Bronchial blocker before and after balloon inflation. (**l**) Bronchoscopy of bronchial blocker positioned in right main stem bronchus: before and after balloon inflation (arrow). (**m**) X-ray of bronchial blocker with inflated balloon.

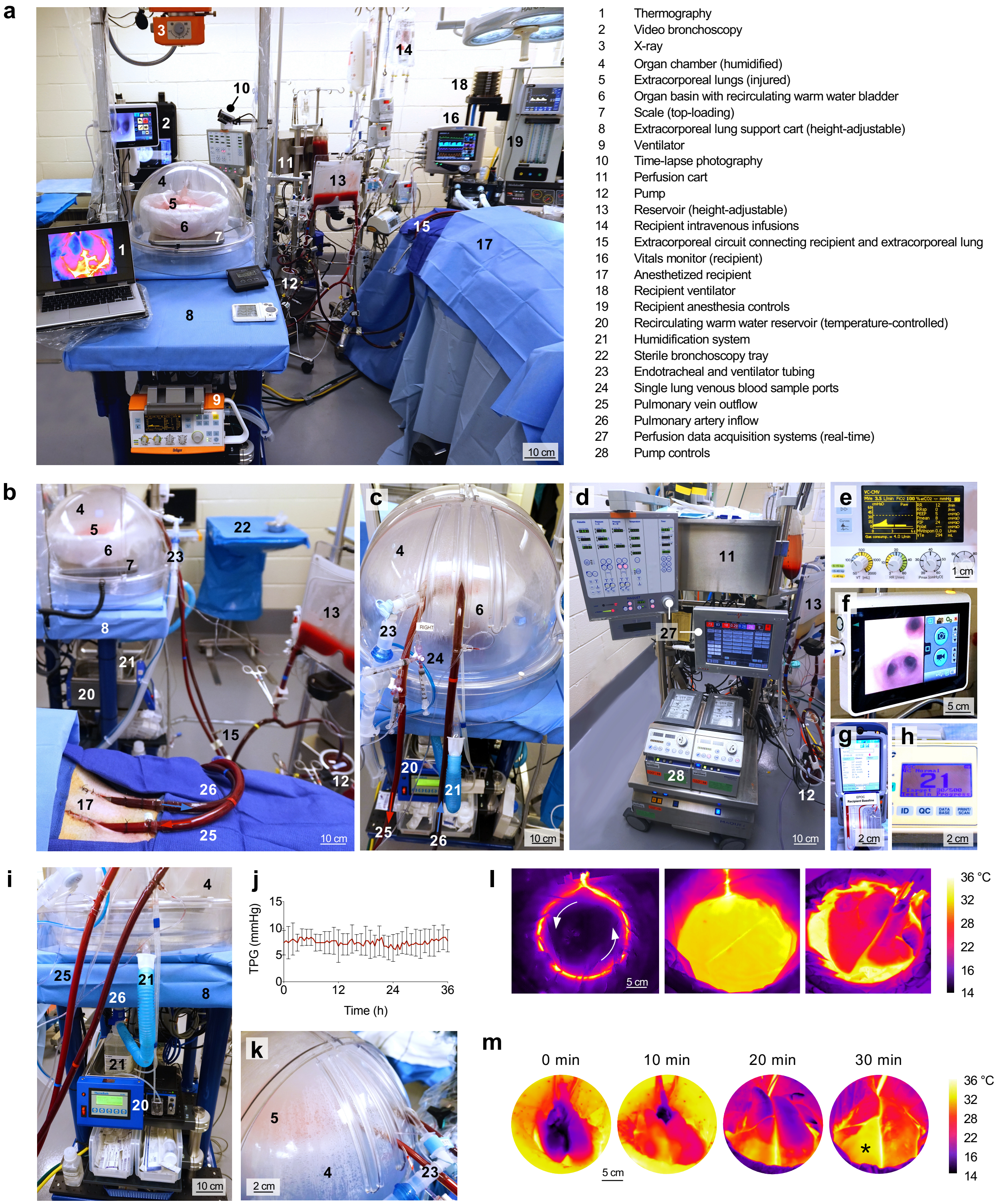

Supplementary Figure 3 | Experimental setup. (**a**) Operating room configuration. Extracorporeal lung support and recovery with (**b**) perfusion circuit, (**c**) humidified, temperature-controlled organ chamber, (**d**) perfusion cart, (**e**) ventilator, (**f**) video bronchoscope, (**g**) hemogas analyzer, and (**h**) activated clotting time (ACT) machine. Extracorporeal lung perfusion and environmental controls including (**i**) temperature-controlled reservoir of recirculating warm water supplying soft bladder on which the extracorporeal lungs are placed in the organ basin, (**j**) maintenance of trans-pulmonary pressure gradient (TPG) between 5 –15 mmHg, and (**k**) air humidification system supplying warm humidified air to organ chamber. (**l**) Thermal imaging of organ basin demonstrating normothermic recirculating warm water, thermal equilibrium of the soft bladder at 32ºC, and lungs on cross-circulation with maintenance of soft bladder at 32ºC. (**m**) Thermal imaging (time-lapse) of rewarming extracorporeal lungs with perfusion and ventilation (initiated after 15 min of perfusion) following procurement. At 30 minutes, extracorporeal lungs were significantly rewarmed, with injured lungs consistently demonstrating elevated surface temperatures, most notably in lower lobes (star).

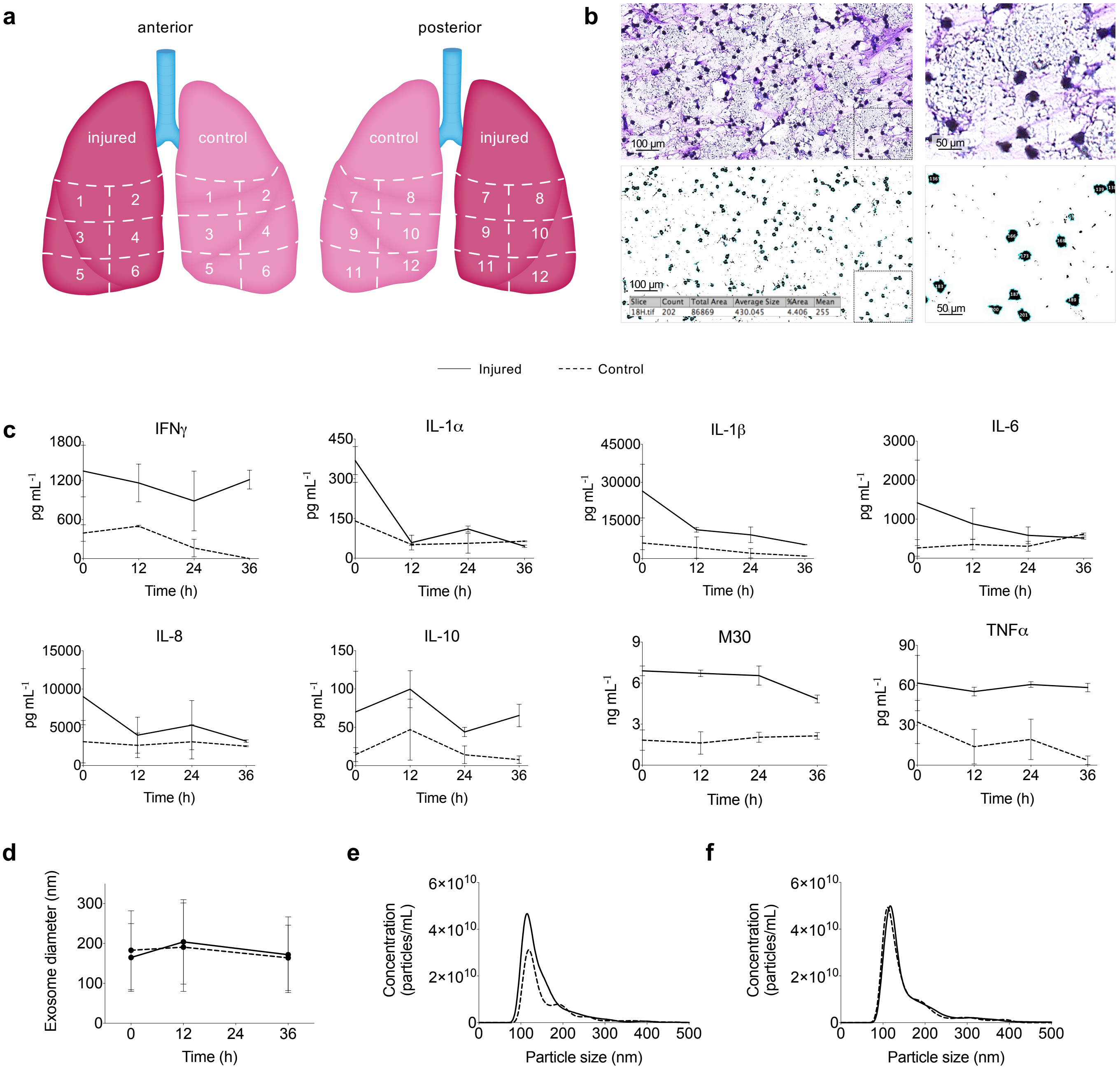

Supplementary Figure 4 | Randomized lung sampling and analyses of BAL fluid samples collected during cross-circulation. (**a**) Lung map used for randomized tissue sampling showing the lung divided into 12 numbered regions with left/right and anterior/posterior divisions. Sample bias was avoided by predetermining the geographic sampling region for each time point prior to the start of experimentation. (**b**) Demonstration of cellular quantification technique enabling quantification of cellular infiltrates in Kwik-Diff staining of BAL fluid smears collected from injured and control lungs. Low and high magnification of raw images and processed images for cell counting. (**c**) Pro-inflammatory, anti-inflammatory, and activation markers in BAL fluid samples collected from injured and control lungs throughout 36 hours of cross-circulation. (**d**) Mean diameter of exosomes isolated from BAL samples collected from injured and control lungs. Mean size distribution of exosomes isolated from BAL samples collected from injured and control lungs at (**e**) 0 hours and (**f**) 36 hours of cross-circulation.

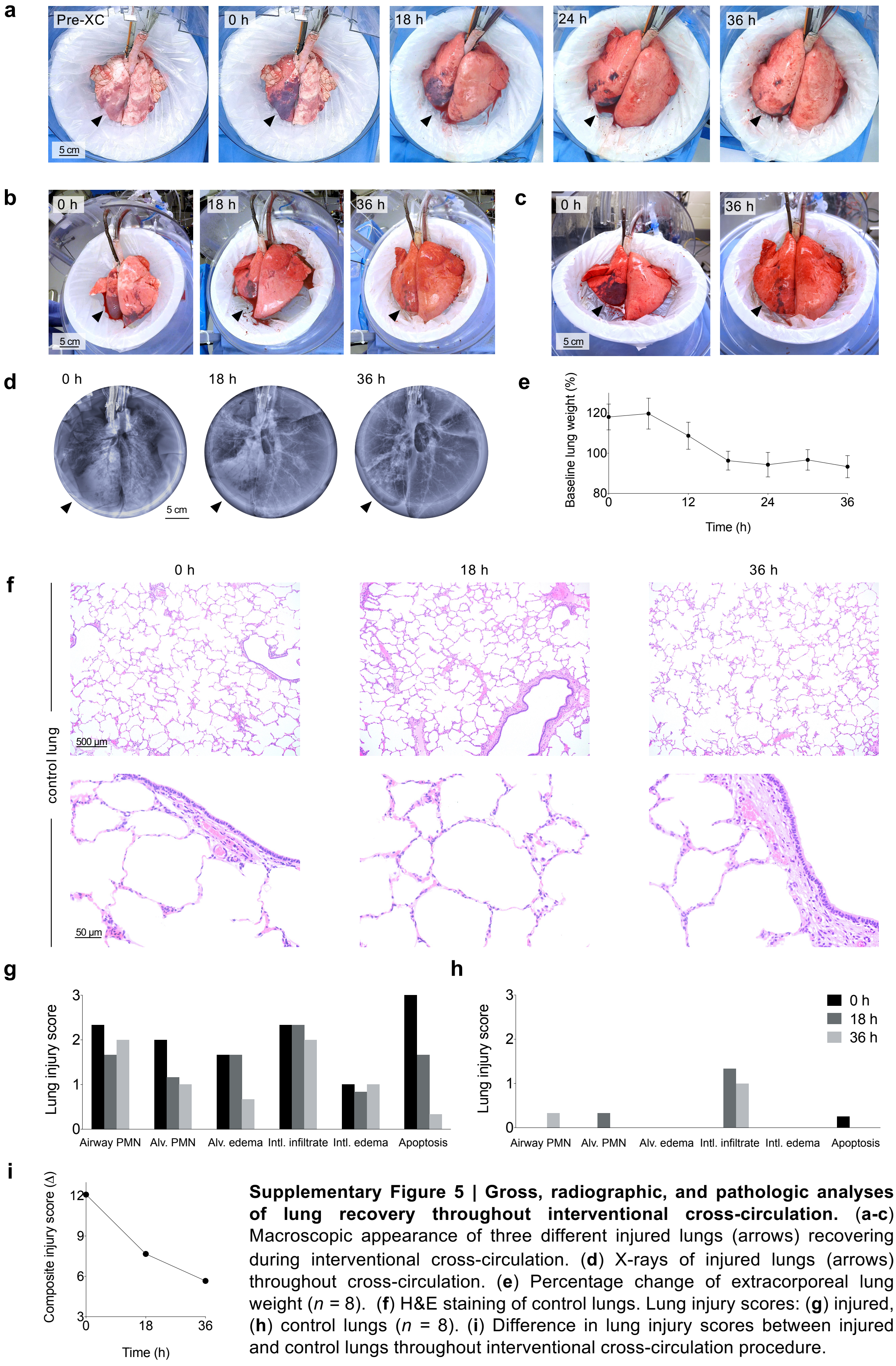

Supplementary Figure 5 | Gross, radiographic, and pathologic analyses of lung recovery throughout interventional cross-circulation. (**a-c**) Macroscopic appearance of three different injured lungs (arrows) recovering during interventional cross-circulation. (**d**) X-rays of injured lungs (arrows) throughout cross-circulation. (**e**) Percentage change of extracorporeal lung weight (n = 8). (**f**) H&E staining of control lungs. Lung injury scores: (**g**) injured, (**h**) control lungs (n = 8). (**i**) Difference in lung injury scores between injured and control lungs throughout interventional cross-circulation procedure.

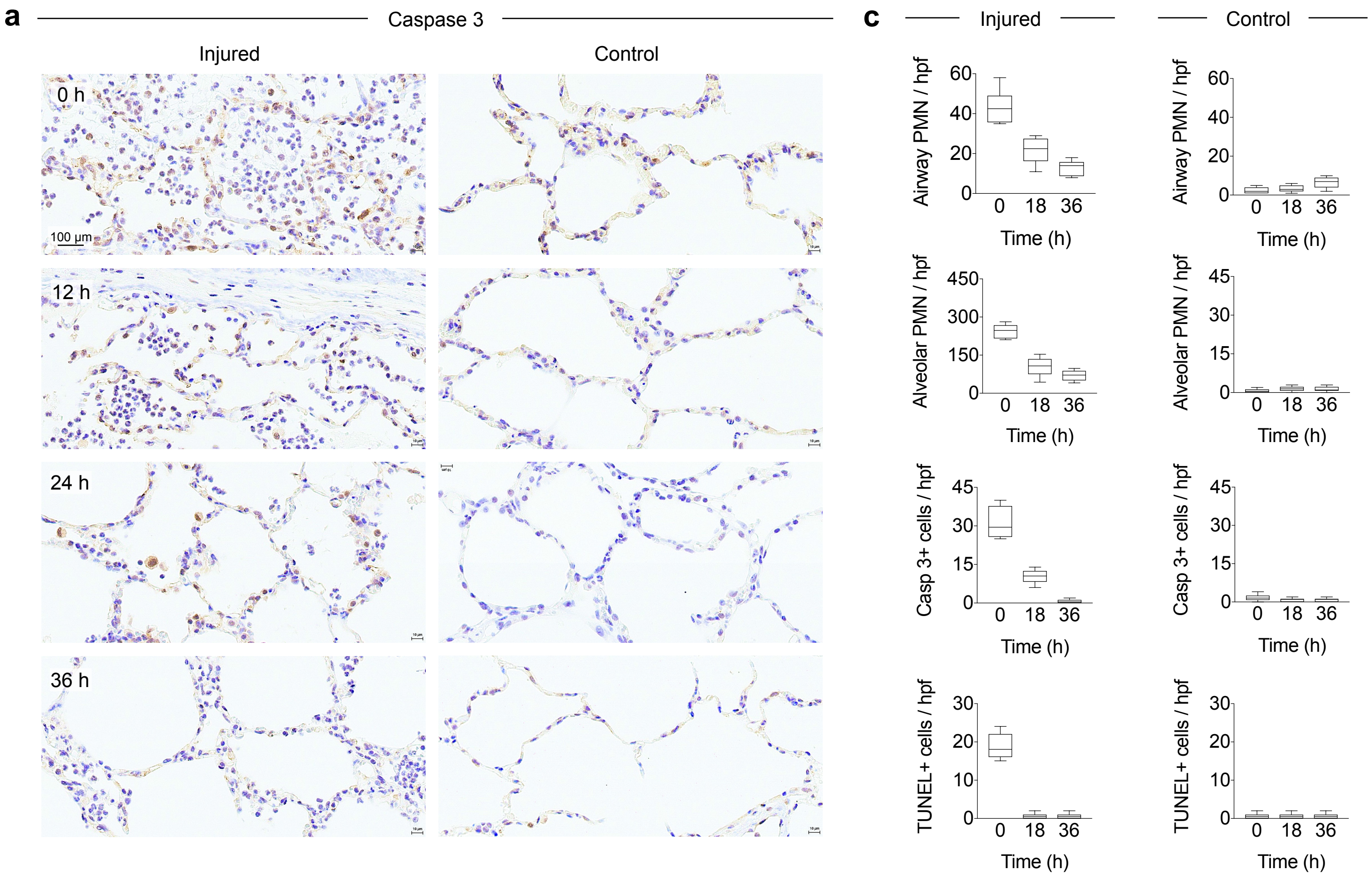

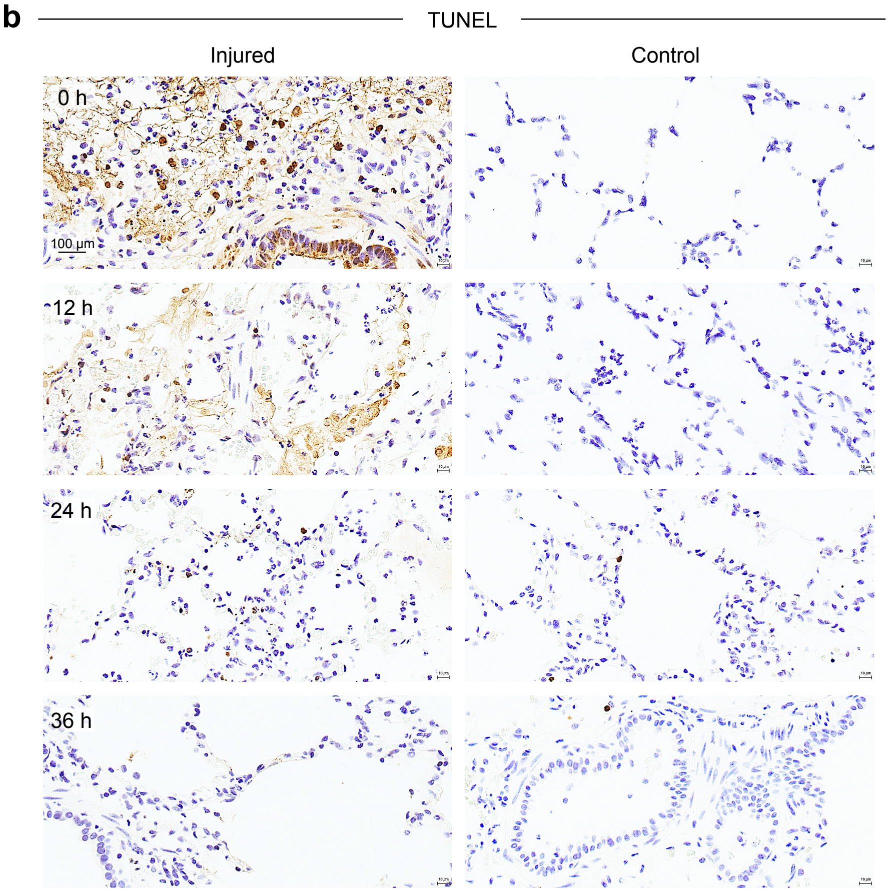

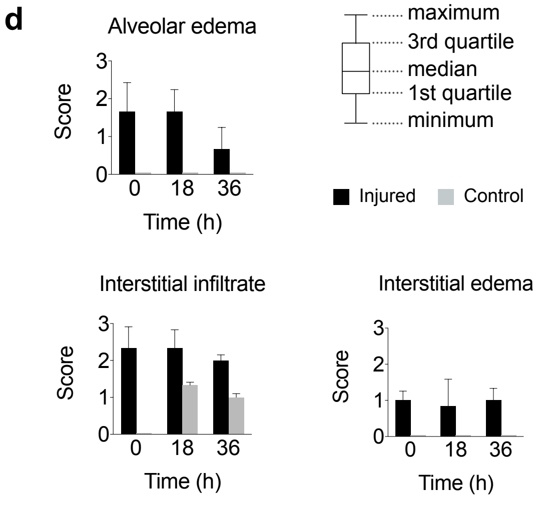

Supplementary Figure 6 | Pathologic immunohistochemical staining, injury scoring, and analysis. Immunostaining of injured and control lungs: (**a**) caspase 3, (**b**) TUNEL. (**c**) Box-and-whisker plot cell counts of airway and alveolar polymorphonuclear cells (PMN), Caspase 3+ cells, and TUNEL+ cells. (**d**) Pathologic scoring (edema, cellular infiltration) of injured and control lungs throughout interventional cross-circulation.

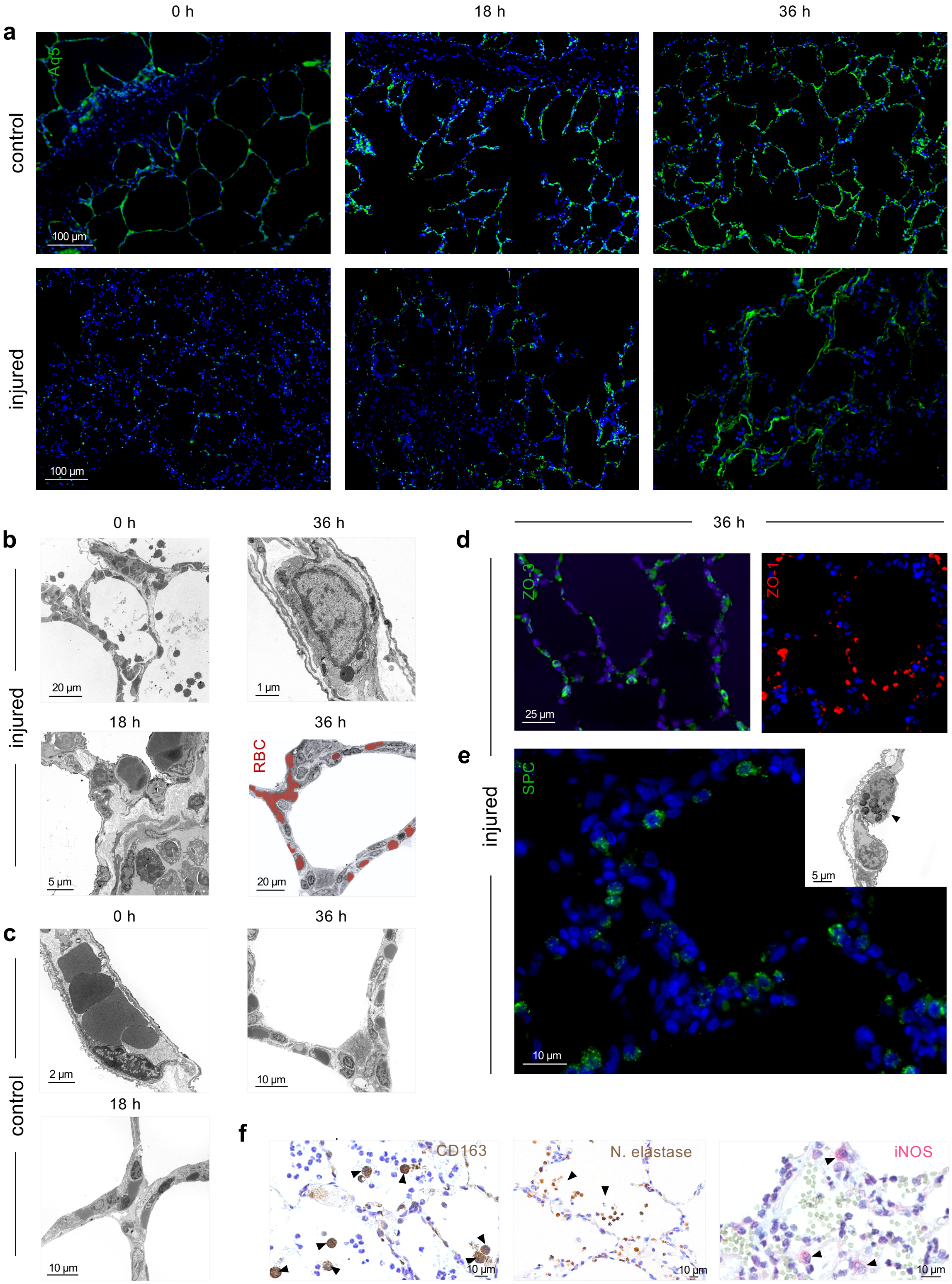

Supplementary Figure 7 | Histological analysis throughout recovery. (**a**) Aquaporin-5 (Aqp 5, type I cell marker) at 0, 18, 36 h in control and healthy lungs. (**b**) TEM of injured lung at 0 h, 18 h, and 36 h. Red blood cells (RBCs) pseudocolored in red. (**c**) TEM of control lung at 0 h, 18 h, and 36 h. (**d**) Injured lung after 36 h of cross-circulation. Immunostaining for ZO-3 (epithelial tight junction protein) and ZO-1 (microvascular tight junction protein). (**e**) Injured lung recovered at 36 h with recovery of SPC and corresponding TEM of type II cell containing lamellar bodies (arrow). (**f**) Representative immunostaining used in histopathologic analysis: CD163, neutrophil elastase, and iNOS.

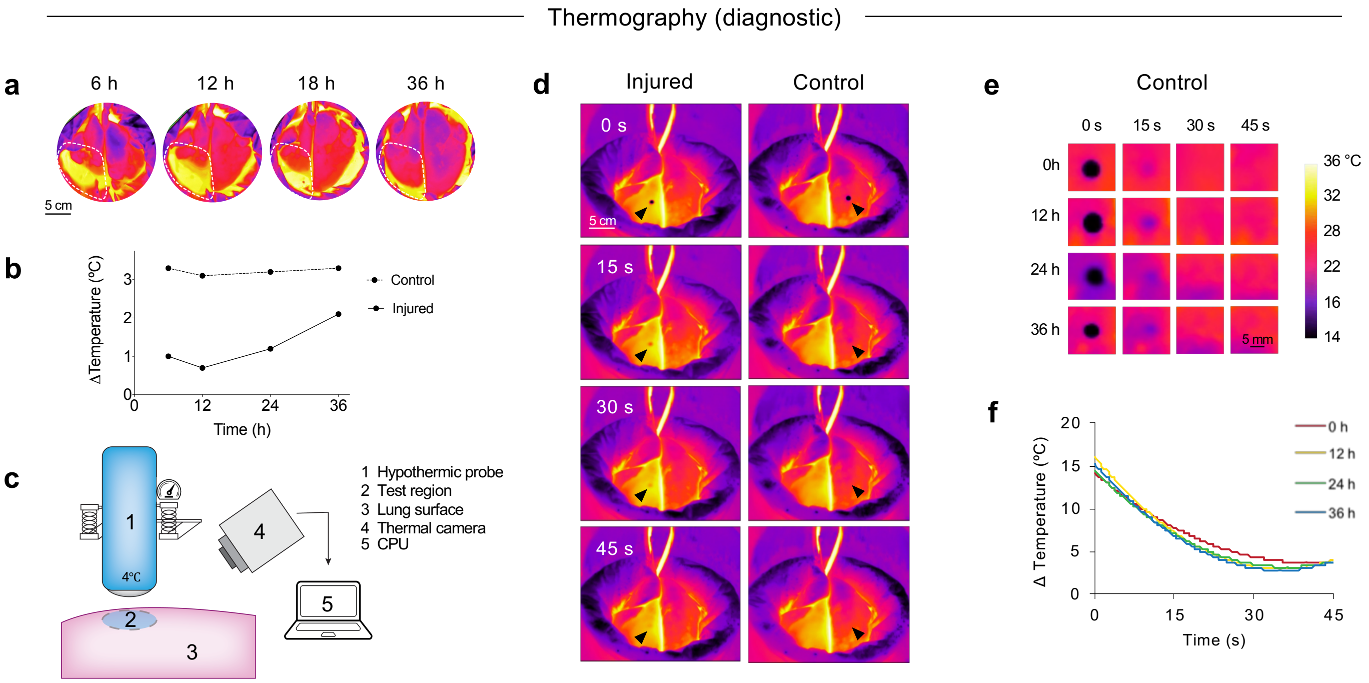

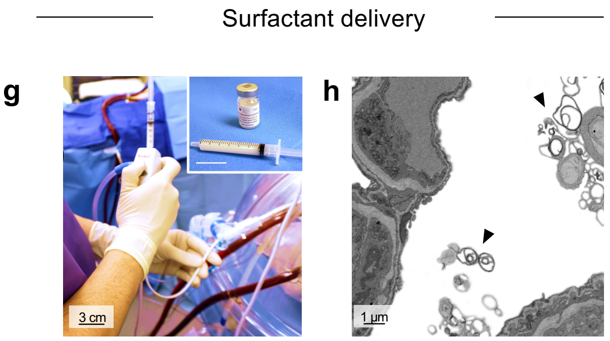

Supplementary Figure 8 Lung thermography and surfactant delivery. (**a**) Surface thermography demonstrates improvement in surface temperature, recruitment, and perfusion during lung recovery. (**b**) Lungs injured by gastric aspiration in the bilateral lower lobes. Thermography shows increased temperatures in the regions corresponding to injury. (**c**) Hypothermic recovery challenge. Time (s) for surface temperature to return to baseline following cold probe challenge after 0, 12, 24, and 36 hours of recovery on cross-circulation. Ambient temperature and humidity were constant for each time point. (**d**) Lungs injured by gastric aspiration in the bilateral lower lobes. Diagnostic thermography shows increased temperatures in injured regions. (**e**) Thermal images from hypothermic tests of control lungs. (**f**) Rewarming curves of control lungs. Surfactant replacement: (**g**) Bronchoscopic delivery of therapeutic surfactant (inset) in injured extracorporeal lungs recovering on cross-circulation. Scale bar: 3 cm. (**h**) Transmission electron microscopy confirming delivery of therapeutic surfactant (arrows) to alveoli of injured lungs.

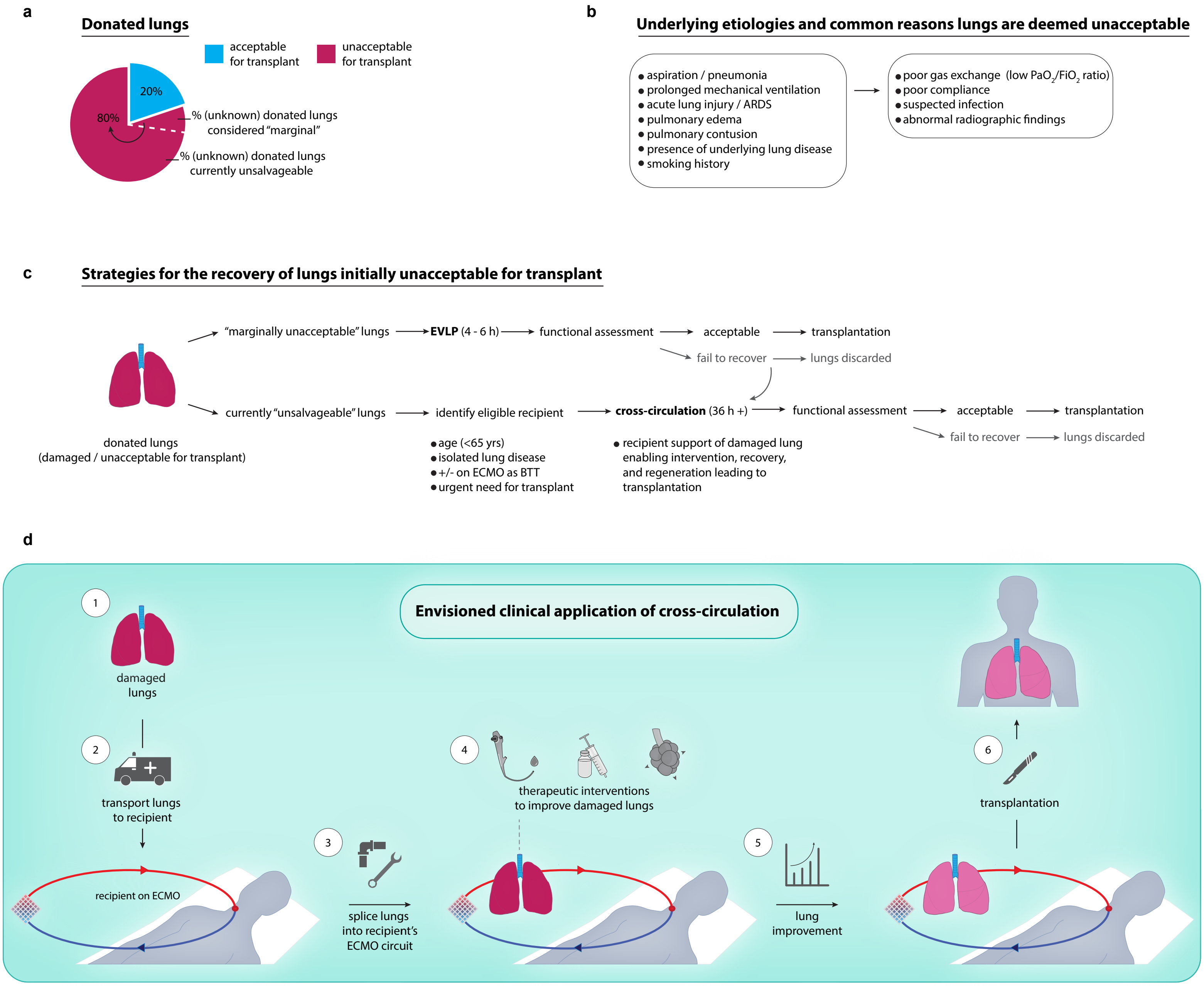

Supplementary Figure 9 | Envisioned clinical application of cross-circulation. (**a**) Percentage of donated lungs which are acceptable and unacceptable for transplantation. (**b**) Most common reasons lungs are deemed unacceptable and not utilized for transplantation, and common etiologies of poor lung function. (**c**) Potential paths for donor lungs deemed unacceptable for transplantation, including proposed use of cross-circulation to enable intervention, recovery, and regeneration in extracorporeal lungs. ECMO: extracorporeal membrane oxygenation; BTT: bridge-to-transplant. (**d**) Envisioned clinical use of cross-circulation bewteen an organ recipient and an extracorporeal organ to enable recovery and eventual transplantation of an organ (e.g., lung, heart, liver, kidney) initially unacceptable for transplantation.

	Cross-circulation	EVLP			
Authors	**Guenthart *et al.***	**Nakajima *et al.*[11]**	**Khalife-Hocquemiller *et al.*[23]**	**Meers *et al.*[21]**	**Inci *et al.*[22]**
Author affiliation	Columbia University	University of Toronto	University of Paris-Sud	Catholic University of Leuven	University of Zurich
Journal		*Journal of Heart & Lung Transplantation*	*Transplantation*	*Journal of Surgical Research*	*Journal of Heart & Lung Transplantation*
Year	(2018)	2017	2014	2011	2008
Title	Regeneration of severely damaged lungs using an interventional cross-circulation platform	Lung lavage and surfactant replace-ment during *ex vivo* lung perfusion for treatment of gastric acid aspiration-induced donor lung injury	Exogenous surfactant attenuates lung injury from gastric-acid aspiration during *ex vivo* reconditioning in pigs	A model of *ex vivo* perfusion of porcine donor lungs injured by gastric aspiration: a step towards pretransplant reconditioning	*Ex vivo* reconditioning of marginal donor lungs injured by acid aspiration
Type of lungs	Swine	Swine	Swine (piglets)	Swine	Swine
Name of experimental group	"Injured"	"Lavage + Surfactant"	"LI-Surf-EVLP"	"Gastric Juice" (GJ)	"SL-Exvivo"
Number of lungs in experimental group (*n*)	8	5	6	6	6
Injury model	Aspiration of gastric contents	Aspiration of gastric acid	Aspiration of gastric contents	Aspiration of gastric juice	Aspiration of betaine-hydrochloric acid/pepsin mixture
Distribution of injury ⁎	Unilateral	Bilateral	Unilateral	Unilateral	Bilateral
Quantity of aspirate (mL kg^{-1} $lung^{-1}$)	2	~1.5	~2	2	~2.5
Organ support device	Non-commercial	XVIVO	Non-commercial	Non-commercial	Non-commercial
Perfusate	Blood	Steen solution™	Steen solution™	Steen solution™	Blood (leukocyte-reduced) + Perfadex®
Duration of normothermic lung perfusion (h)	36	6	4	2	2
Total extracorporeal preservation time (h)	38	18	~4	3.25	5
Transplantation after lung perfusion	No	Left lung	No	No	No
Duration of post-transplant assessment (h)	–	4	–	-	–

⁎ In studies that reported intratracheal aspiration of gastric acid, distribution of injury was assumed to be bilateral with equal quantity of aspirate into the left and right lungs.

Supplementary Table 1 | Description of studies investigating the use of cross-circulation or *ex vivo* lung perfusion (EVLP) in swine models of aspiration injury. In all studies, swine lungs injured by gastric aspiration were supported by cross-circulation (36 hours) or *ex vivo* lung perfusion (2 – 6 hours). Each study is represented by the experimental group that was deemed most comparable to the "Injured" experimental group in Guenthart *et al*. Data not reported as explicit numerical values were approximated or calculated from other reported information, and are indicated by ~. All data were retrieved from main figures and text, or from Supplementary Information. EVLP, *ex vivo* lung perfusion.

	Guenthart *et al.*	Nakajima *et al.*[11]	Khalife *et al.*[23]	Meers *et al.*[21]	Inci *et al.*[22]
**Experimental parameters**					
Lung support strategy	Cross-circulation	EVLP	EVLP	EVLP	EVLP
Duration of normothermic lung perfusion (h)	36	6	4	2	2
Number of lungs in experimental group (*n*)	8	5	6	6	6
**PaO_2/FiO_2** (mmHg)					
Initial	90	NR	~350	~160	~400
Final	750	NR	~450	~130	~400
**Δ**	▲ **660**	–	▲ **~100**	▼ **~30**	**~0**
**Compliance** (mL cmH_2O^{-1})					
Initial	4	~23	NR	~6	~15
Final	15	~29	NR	~9	~12
**Δ**	▲ **11**	▲ **~6**	–	▲ **~3**	▼ **~3**
**Peak inspiratory pressure** (mmHg)					
Initial	21	NR	NR	~33	NR
Final	12	NR	NR	30	NR
**Δ**	▼ **9**	–	–	▼ **3**	–
**Pulmonary vascular resistance** (dyn•s cm^{-5})					
**Δ**	NR *	▼ **~80**	▼ **~100**	▲ **~500**	**0**
**Lung weight** (% change)					
**Δ**	▼ **20**	NR	NR	NR **	NR ***

* Guenthart *et al.* report transpulmonary pressure gradient (TPG, the difference in vascular pressure between the pulmonary artery and veins, **Supplementary Fig. 3c, ii**), which was maintained between ~5 – 10 mmHg to protect the pulmonary vasculature throughout 36 hours of cross-circulation.

** Meers *et al.* report wet to dry weight ratio of left and right lungs as a surrogate marker of lung edema. Left and right lungs in the experimental group "GJ" had significantly higher wet to dry weight ratios compared with uninjured controls.

*** Inci *et al.* report wet to dry weight ratio of the left upper lobe only as a surrogate marker of lung edema.

Supplementary Table 2 | Comparison of lung function data reported in studies investigating the use of cross-circulation or ex vivo lung perfusion (EVLP) in swine models of aspiration injury. In all studies, swine lungs injured by gastric aspiration were supported by cross-circulation (36 h) or ex vivo lung perfusion (2 – 6 h). All values represent means. Quantitative data (means) that were not reported as explicit numerical values were approximated from graphs or calculated from initial and final values approximated from graphs, and are indicated by ~. Initial values correspond to data collected at the initiation of normothermic lung perfusion. Final values correspond to data collected at the conclusion of normothermic lung perfusion. Change values (**Δ**) correspond to differences between initial and final values. Data not reported are indicated by NR. Green arrow (▲ or ▼) indicates an increase or improvement in lung function from initial to final time point. Red arrow (▲ or ▼) indicates a decrease or worsening in lung function from initial to final time point. All data were retrieved from main figures and text, or from Supplementary Information. EVLP, ex vivo lung perfusion; NR: Not reported.

	Guenthart *et al.*	Nakajima *et al.*[11]	Khalife *et al.*[23]	Meers *et al.*[21]	Inci *et al.*[22]
**Experimental parameters**					
Lung support strategy	Cross-circulation	EVLP	EVLP	EVLP	EVLP
Duration of normothermic lung perfusion (h)	36	6	4	2	2
Number of lungs in experimental group (*n*)	8	5	6	6	6
**BAL fluid**					
**pH**					
Initial	4.5	NR	NR	NR	NR
Final	5.5	NR	NR	NR	NR
**Δ**	▲ **1**	–	–	–	–
**Pepsin** (ng mL^{-1})					
**Δ**	▼ **106**	NR	NR	NR	NR
**Total protein** (mg mL^{-1})					
**Δ**	▼ **0.47**	NR *	NR	NR	▲ **0.19**
**Cells per high power field**					
**Δ**	▼ **1696**	NR	NR	**0**	NR
**IL-1β** (pg mL^{-1})					
Initial	26554	NR	NR	NR	~11
Final	5508	~1500	~1400	NR	~8
**Δ**	▼ **21046**	–	–	–	▼ **~3**
**IL-6** (pg mL^{-1})					
Initial	1418	NR	NR	NR	~80
Final	517	~1400	~65	NR	~180
**Δ**	▼ **901**	–	–	–	▲ **~100**
**IL-8** (pg mL^{-1})					
Initial	9007	NR	NR	NR	~20
Final	3188	~5000	~12	NR	~220
**Δ**	▼ **5819**	–	–	–	▲ **~200**
**IL-10** (pg mL^{-1})					
Initial	70	NR	NR	NR	~10
Final	65	NR	NR	NR	~10
**Δ**	▼ **5**	–	–	–	**~0**
**IFNγ** (pg mL^{-1})					
Initial	1348	NR	NR	NR	NR
Final	1216	NR	NR	NR	NR
**Δ**	▼ **132**	–	–	–	–
**TNFα** (pg mL^{-1})					
Initial	62	NR	NR	NR	NR
Final	58	NR	NR	NR	NR
**Δ**	▼ **4**	–	–	–	–
**Perfusate**					
**pH**					
Initial	7.45	NR	NR	NR	NR
Final	7.38	NR	NR	NR	NR
**Δ**	**0.07**	–	–	–	–
**Lactate** (mmol L^{-1})					
Initial	1.86	NR	NR	NR	NR
Final	1.04	NR	NR	NR	NR
**Δ**	▼ **0.82**	–	–	–	–
**IL-1β** (pg mL^{-1})					
**Δ**	▼ **318**	NR	NR	NR	NR
**IL-6** (pg mL^{-1})					
**Δ**	▲ **9**	NR	NR	NR	NR
**IL-8** (pg mL^{-1})					
**Δ**	▲ **206**	NR	NR	NR	NR
**IL-10** (pg mL^{-1})					
**Δ**	▼ **104**	NR	NR	NR	NR
**IFNγ** (pg mL^{-1})					
**Δ**	▼ **904**	NR	NR	NR	NR
**TNFα** (pg mL^{-1})					
**Δ**	▼ **14**	NR	NR	NR	NR

* Nakajima *et al.* does not report initial total protein, but does report final total protein: 2.1 ± 1.2 mg mL^{-1}.

Supplementary Table 3 | Comparison of bronchoalveolar lavage (BAL) fluid and perfusate data reported in studies investigating the use of cross-circulation or ex vivo lung perfusion (EVLP) in swine models of aspiration injury. In all studies, swine lungs injured by gastric aspiration were supported by cross-circulation (36 h) or ex vivo lung perfusion (2 – 6 h). All values represent means. Quantitative data (means) that were not reported as explicit numerical values were approximated from graphs or calculated from initial and final values approximated from graphs, and are indicated by ~. Initial values correspond to data collected at the initiation of normothermic lung perfusion. Final values correspond to data collected at the conclusion of normothermic lung perfusion. Change values (**Δ**) correspond to differences between initial and final values. Data not reported are indicated by NR. Green arrow (▲ or ▼) indicates an increase or improvement in lung function from initial to final time point. Red arrow (▲ or ▼) indicates a decrease or worsening in lung function from initial to final time point. All data were retrieved from main figures and text, or from Supplementary Information. EVLP, ex vivo lung perfusion; BAL, bronchoalveolar lavage; NR, Not reported.

	Guenthart *et al.*	Nakajima *et al.*[11]	Khalife *et al.*[23]	Meers *et al.*[21]	Inci *et al.*[22]
**Experimental parameters**					
Lung support strategy	Cross-circulation	EVLP	EVLP	EVLP	EVLP
Duration of normothermic lung perfusion (h)	36	6	4	2	2
Number of lungs in experimental group (*n*)	8	5	6	6	6
**Lung function analysis**					
Pressure-volume loops	8	0	0	0	0
**Lung imaging analysis**					
Photographs (gross)	14	0	1	3	0
Radiographs	11	0	0	0	0
Thermographs	21	0	0	0	0
Bronchographs	18	0	0	0	0
Total lung images	64	0	1	3	0
**BAL fluid analysis**					
Bacterial cultures	Negative	NR	Positive	NR	NR
Cytokines reported	16	3	0	0	4
**Perfusate analysis**					
Biochemical markers reported	30	0	0	0	0
Cytokines reported	14	0	6	0	0
**Cell viability and function analysis**					
Metabolic activity assays	1	0	0	0	0
Functional uptake assays	3	0	0	0	0
Airway cilia images	2	0	0	0	0
Apoptosis markers	2	0	1	0	0
**Microscopic imaging analysis**					
H&E micrographs	21	0	0	3	0
Special histologic stain micrographs	45	0	0	0	0
Scanning electron micrographs	3	0	0	0	0
Transmission electron micrographs	10	0	0	0	0
Total micrographs	79	0	0	3	0
**Histopathologic analysis**					
Injury categories reported	6	0	5	0	5
**Immunostaining analysis**					
Cell markers	6	0	0	0	0
Tight junction markers	2	0	0	0	0
Gap junction markers	1	0	0	0	0

Supplementary Table 4 | Comparison of scope of analysis and data reported in studies investigating the use of cross-circulation or ex vivo lung perfusion (EVLP) in swine models of aspiration injury. All values (unless otherwise indicated) represent the total number of countable data (e.g., number of photographs, number of cytokines, number of cell markers) across all experimental groups reported in each study. All data were retrieved from main figures and text, or from Supplementary Information. EVLP, ex vivo lung perfusion; NR, Not reported.

Donor response to gastric aspiration (*n* = 8)

	Baseline	6 h after aspiration	% Change	Fold above control
**Inflammatory cytokines: BAL fluid (injured lungs)**				
GM-CSF (pg/mL)	45.1 ± 33.4	101.5 ± 9.1	▲ 125.1%	6.5
IFNγ (pg/mL)	271.8 ± 55.3	1700.9 ± 250.9	▲ 525.8%	22.7
IL-1α (pg/mL)	15.8 ± 15.2	1019.2 ± 438.1	▲ 6350.6%	4.5
IL-1β (pg/mL)	100.9 ± 107.9	40170.7 ± 10783.3	▲ 39712.4%	8.9
IL-1rα (pg/mL)	92.3 ± 55.9	14664.3 ± 5608.5	▲ 15787.6%	12.1
IL-2 (pg/mL)	10.3 ± 2.2	70.1 ± 36.8	▲ 580.6%	6
IL-4 (pg/mL)	8.9 ± 2.2	93.1 ± 44.8	▲ 946.1%	6.9
IL-6 (pg/mL)	3.3 ± 1.6	2358.4± 1915.2	▲ 71366.7%	22.8
IL-8 (pg/mL)	336.8 ± 447.2	8838.5 ± 137.8	▲ 2524.3%	2.42
IL-10 (pg/mL)	11.1 ± 9.7	127.6 ± 72.2	▲ 1049.5%	38.9
IL-12 (pg/mL)	6.4 ± 5.4	105.6 ± 30.2	▲ 1550%	7.3
IL-18 (pg/mL)	110.9 ± 85.9	1247.9 ± 75.9	▲ 1025.2%	6.3
TNFα (pg/mL)	5.1 ± 8.9	67.6 ± 8.8	▲ 1225.5%	3
**Inflammatory cytokines: BAL fluid (control lungs)**				
GM-CSF (pg/mL)	45.1 ± 33.4	53.7 ± 13.7	▲ 19.1%	–
IFNγ (pg/mL)	271.8 ± 55.3	334.9 ± 234.7	▲ 23.2%	–
IL-1α (pg/mL)	15.8 ± 15.2	240.0 ± 157.5	▲ 1419%	–
IL-1β (pg/mL)	100.9 ± 107.9	4622.5 ± 4699.9	▲ 4481.2%	–
IL-1rα (pg/mL)	92.3 ± 55.9	1295.3 ± 1265.6	▲ 1303.4%	–
IL-2 (pg/mL)	10.3 ± 2.2	20.3 ± 12.3	▲ 97.1%	–
IL-4 (pg/mL)	8.9 ± 2.2	21.1 ± 21.9	▲ 137.1%	–
IL-6 (pg/mL)	3.3 ± 1.6	106.6 ± 97.6	▲ 3130.3%	–
IL-8 (pg/mL)	336.8 ± 447.2	3847.7 ± 796.1	▲ 1042.2%	–
IL-10 (pg/mL)	11.1 ± 9.7	14.1 ± 9.7	▲ 27%	–
IL-12 (pg/mL)	6.4 ± 5.4	19.9 ± 13.8	▲ 210.9%	–
IL-18 (pg/mL)	110.9 ± 85.9	290.6 ± 159.2	▲ 162%	–
TNFα (pg/mL)	5.1 ± 8.9	25.6 ± 24.5	▲ 402%	–
**Inflammatory cytokines: serum**				
GM-CSF (pg/mL)	28.3 ± 32.8	38.4 ± 30.9	▲ 35.5%	–
IFNγ (pg/mL)	67.3 ± 105.6	2201.8 ± 1746.9	▲ 3171.6%	–
IL-1α (pg/mL)	17.1 ± 9.6	18.64 ± 10.1	▲ 9%	–
IL-1β (pg/mL)	228.5 ± 67.8	230.5 ± 58.5	▲ 0.9%	–
IL-1rα (pg/mL)	2334.5 ± 2414.6	6680.1 ± 5080.9	▲ 186.1%	–
IL-2 (pg/mL)	109.8 ± 47.2	104.5 ± 34.2	▼4.8%	–
IL-4 (pg/mL)	173.7 ± 119.3	174.3 ± 121.6	▲ 0.3%	–
IL-6 (pg/mL)	25.1 ± 32.2	64.5 ± 26.3	▲ 157%	–
IL-8 (pg/mL)	166.2 ± 108.2	330.1 ± 301.6	▲ 98.6%	–
IL-10 (pg/mL)	142.8 ± 84.2	149.1 ± 84.3	▲ 4.4%	–
IL-12 (pg/mL)	339.6 ± 104.6	293.1 ± 40.3	▼ 13.7%	–
IL-18 (pg/mL)	2576.1 ± 1639.7	1805.9 ± 1161.9	▼ 29.9%	–
P-Selectin (ng/mL)	2.1 ± 0.5	22.5 ± 0.6	▲ 971.4%	–
TNFα (pg/mL)	26.6 ± 16.9	62.2 ± 57.5	▲ 133.8%	–
**Hemolytic markers**				
D-Dimer (μg/L)	122 ± 43.2	166 ± 61.5	▲ 36%	–
Fibrinogen (mg/dL)	425 ± 27.3	478 ± 16.6	▲ 12.5%	–
Free Hgb (ng/mL)	1.13 ± 1.08	12.82 ± 1.49	▲ 1034.5%	–

Supplementary Table 5 | Donor response to gastric aspiration. Analysis of inflammatory cytokines in donor bronchoalveolar lavage (BAL) fluid collected from injured and control lungs. Additionally, inflammatory cytokines and hemolytic markers were quantified in donor serum (*n* = 8). Direction of arrow indicates increase (up arrow) or decrease (down arrow) in concentration of cytokine or hemolytic marker 6 hours after gastric aspiration. Yellow arrow (▲) indicates percent change in concentration between 0.1% and 499.9%. Red arrow (▲) indicates percent change in concentration greater than 500%. All values represent mean ± standard deviation. Hgb: hemoglobin.

Score	0	1	2	3
Airway PMN / hpf [1]	0	1 – 25	26 – 50	> 50
Alveolar PMN / hpf [2]	0	1 – 25	26 – 50	> 50
Alveolar edema (%) [3]	< 5	6 – 25	26 – 50	> 50
Interstitial infiltrate / hpf [4]	none	< 50	50 – 100	> 100
Interstitial edema [5]	0	1x width vessel media	≥ 2x width vessel media	–
Apoptotic cells / hpf	< 2	3 – 5	6 – 10	> 10

[1] Airway PMN: % bronchi and bronchioles containing any neutrophils
[2] Alveolar PMN: % alveoli more than half-filled with neutrophils
[3] Alveolar edema: % alveoli with edema
[4] Interstitial infiltrate: lymphocytes/neutrophils in interstitium around vessels and airways and in alveolar septa and pleura
[5] Interstitial edema: perivascular and peribronchial spaces expanded with edematous fluid

Supplementary Table 6 | Scoring rubric of lung injury score. PMN: polymorphonuclear cells, hpf: high-power field.

Recipient safety data (n = 8)

Time (h)	0	6	12	18	24	30	36
**Vitals**							
Heart rate (bpm)	109 ± 19	104 ± 14	93 ± 13	95 ± 12	83 ± 24	83 ± 34	89 ± 46
Systolic BP (mmHg)	90 ± 9	102 ± 11	93 ± 17	92 ± 6	96 ± 12	88 ± 5	84 ± 10
Temperature (ºF)	95.7 ± 2.5	97.8 ± 2.9	96.5 ± 0.4	98.3 ± 0.5	97.9 ± 0.2	97.5 ± 0.7	96.4 ± 1.7
SpO_2 (%)	96 ± 0.1	98 ± 0.7	98 ± 0.4	97 ± 1.0	98 ± 1.4	96 ± 1.8	96 ± 3.7
**Hemogas**							
pH	7.45 ± 0.01	7.43 ± 0.03	7.44 ± 0.02	7.43 ± 0.01	7.40 ± 0.04	7.41 ± 0.03	7.38 ± 0.02
pO_2 (mmHg)	500 ± 94	544 ± 39	514 ± 31	464 ± 27	548 ± 42	526 ± 23	514 ± 65
pCO_2 (mmHg)	42 ± 6.4	46 ± 2.0	44 ± 7.3	44 ± 0.8	48 ± 4.7	47 ± 2.0	50 ± 1.7
HCO_3 (mmol/L)	31 ± 3.3	31 ± 1.6	30 ± 5.6	30 ± 1.4	30 ± 1.3	30 ± 1.1	30 ± 1.9
Lactate (mmol/L)	1.86 ± 0.41	2.21 ± 0.61	1.71 ± 0.33	1.37 ± 0.12	0.94 ± 0.1	1.05 ± 1.4	1.04 ± 0.05
Glucose (mg/dL)	83 ± 9	107 ± 11	123 ± 27	130 ± 23	120 ± 26	105 ± 25	102 ± 39
**Biochemical Analysis**							
WBC (10^9/L)	12.7 ± 3.1	10.8 ± 0.7	10.3 ± 2.4	10.1 ± 0.6	8.9 ± 0.5	9.2 ± 0.9	7.2 ± 1.7
% Neutrophils	33 ± 12	57 ± 20	69 ± 7	71 ± 2	62 ± 3	59 ± 28	60 ± 17
% Reticulocytes	2 ± 1	3 ± 2	3 ± 1	4 ± 1	2 ± 2	3 ± 2	2 ± 1
Platelets (10^9/L)	352 ± 90.6	311.5 ± 45.9	328 ± 63.8	234 ± 46.7	242.5 ± 34.7	205.5 ± 51.6	199 ± 8.5
Hgb/Hct (gdL^{-1} / %)	6.7/22.2 ± 1.1/2.7	7.6/25.1 ± 0.4/0.6	7.5/24.3 ± 1.2/3.3	6.2/21.1 ± 0.2/0.7	8.0/26.4 ± 1.4/1.5	5.2/16.9 ± 1.6/2.5	6.3/20.4 ± 1.6/1.9
AST/ALT (U/L)	33/37 ± 12.5/14.5	39/27 ± 21.6/4.9	50/34 ± 24.1/13.7	26/18 ± 10.6/2.83	54/35 ± 16.3/12.7	139/30 ± 14/10.6	539/66 ± 141/31.8
Creatinine (mg/dL)	0.87 ± 0.12	0.85 ± 0.07	0.8 ± 0.26	0.85 ± 0.07	0.65 ± 0.35	0.75 ± 0.07	0.7 ± 0.14
**Hemolysis**							
LDH (U/L)	387 ± 100	291 ± 28	339 ± 97	202 ± 10	334 ± 35	397 ± 91	458 ± 31
D-Dimer (µg/L)	213 ± 12.7	–	152 ± 7.5	–	167 ± 9.9	–	171 ± 9.1
Fibrinogen (mg/dL)	215± 22.7	–	262 ± 20.8	–	241 ± 10.8	–	255 ± 11.3
P-Selectin (ng/mL)	10.4 ± 0.6	–	11.8 ± 0.6	–	11.5 ± 0.5	–	11.7 ± 0.7
Free Hgb (ng/mL)	5.8 ± 1.7	–	7.9 ± 2.1	–	7.8 ± 1.5	–	8.7 ± 1.2
ACT (s)	–	218 ± 97	344 ± 47	330 ± 66	291 ± 75	299 ± 47	306 ± 58
**Electrolytes and Other**							
Na^+ (mmol/L)	135 ± 0.9	134 ± 1.8	133 ± 0.7	134 ± 0.7	136 ± 1.1	139 ± 1.4	139 ± 2.6
K^+ (mmol/L)	3.8 ± 0.3	3.6 ± 0.4	3.6 ± 0.4	3.8 ± 0.3	4 ± 0.5	4.1 ± 0.3	4.4 ± 0.4
Ca^{++} (mg/dL)	9.8 ± 1.5	9.9 ± 1.7	10.2 ± 1.4	8.1 ± 0.2	10.4 ± 0.1	9.1 ± 1.7	9.6 ± 1.3
Phosphate (mmol/L)	9.1 ± 1.8	9.1 ± 2.6	9.6 ± 2.6	7.2 ± 0.4	10.9 ± 0.9	7.9 ± 2.1	10.9 ± 0.4
CPK (U/L)	459 ± 266	313 ± 53	571 ± 329	257 ± 26	823 ± 86	1211 ± 533	1159 ± 59
PT (s)	15.4 ± 0.6	13.7 ± 2.7	14.1 ± 2.1	13.1 ± 1.3	14.9 ± 0.2	14.8 ± 2.2	16 ± 0.6

Supplementary Table 7 | Recipient safety data. Analysis of recipient vitals, hemogas, biochemistry, hemolysis, and electrolytes throughout 36 hours of cross-circulation (n = 8). All values represent mean ± standard deviation. BP: blood pressure, WBC: white blood cells, Hgb: hemoglobin, Hct: hematocrit, AST: aspartate transaminase, ALT: alanine transaminase, LDH: lactate dehydrogenase, ACT: activated clotting time, CPK: creatine phosphokinase, PT: prothrombin time.

Cytokines: Recipient serum (*n* = 8)

Time (h)	0	12	24	36
**Inflammatory cytokines**				
GM-CSF (pg/mL)	53.5 ± 19.5	82.2 ± 59.9	55.5 ± 57.5	39.2 ± 44.2
IFNγ (pg/mL)	3829.8 ± 1098.5	5941.9 ± 5868.4	3328.2 ± 3329.2	2925.5 ± 2932.3
IL-1α (pg/mL)	21.4 ± 19.1	20.9 ± 19.4	6.5 ± 2.9	11.9 ± 2.5
IL-1β (pg/mL)	329.8 ± 200.3	296.2 ± 183.8	163.3 ± 48.6	162.3 ± 38.7
IL-1rα (pg/mL)	9197.6 ± 5086.5	37923.1 ± 29363.2	3825.9 ± 3019.1	4033.7 ± 3348.5
IL-2 (pg/mL)	105.2 ± 65.9	123.6 ± 92.9	51.7 ± 12.3	52.4 ± 8.4
IL-4 (pg/mL)	303.9 ± 288.5	275.4 ± 278.3	75.6 ± 24.3	62.7 ± 7.1
IL-6 (pg/mL)	62.6 ± 60.5	101.5 ± 75.5	41.3 ± 14.1	71.2 ±19.5
IL-8 (pg/mL)	102.7 ± 100.7	83.1 ± 46.1	108.9 ± 41.6	309.6 ± 241.8
IL-10 (pg/mL)	127.6 ± 116.9	137.5 ± 138.3	23.1 ± 9.3	23.9 ± 5.2
IL-12 (pg/mL)	201.2 ±140.4	119.4 ± 80.3	38.7 ± 27.3	52.8 ± 3.7
IL-18 (pg/mL)	893.1 ± 375.8	925.3 ± 491.6	634.4 ± 43.5	373.3 ± 62.1
P-selectin (ng/mL)	10.4 ± 0.6	11.8 ± 0.6	11.5 ± 0.5	11.7 ± 0.7
TNFα (pg/mL)	130.6 ± 27.5	180.7 ± 124.1	122.6 ± 120.2	116.12 ± 111.7

Supplementary Table 8 | Inflammatory cytokines in recipient serum. Analysis of inflammatory cytokines in recipient serum throughout 36 hours of cross-circulation (*n* = 8). All values represent mean ± standard deviation.

Cytokines: BAL fluid collected from extracorporeal lungs (*n* = 8)

Time (h)	0	12	24	36
**Injured lungs**				
GM-CSF (pg/mL)	102.1 ± 20.4	96.5 ± 12.3	81.7 ± 13.6	92.1 ±12.9
IFNγ (pg/mL)	1347.7 ± 397.5	1164.9 ± 289.9	887.3 ± 458.8	1215.6 ± 144.6
IL-1α (pg/mL)	368.9 ± 52.9	58.9 ± 27.8	110.4 ± 11.2	45.1 ± 4.6
IL-1β (pg/mL)	26554.3 ± 10589.2	11361.7 ± 915.7	9355.2 ± 3090.4	5508.8 ± 20.5
IL-1rα (pg/mL)	12844.5 ± 7229.3	14176.4 ± 5176.2	8037.5 ± 4431.9	13371.9 ± 39.9
IL-2 (pg/mL)	43.6 ± 16.9	34.9 ± 2.6	32.4 ± 5.7	32.4 ± 5.1
IL-4 (pg/mL)	56.9 ± 20.6	46.8 ± 21.1	45.7 ± 11.5	48.9 ± 14.4
IL-6 (pg/mL)	1418.4 ± 999.8	881.5 ± 346.5	585.9 ± 185.6	517.1 ± 20.3
IL-8 (pg/mL)	9007.6 ± 3671.3	3968.2 ± 2353.6	5285.5 ± 3215.5	3188.4 ± 169.2
IL-10 (pg/mL)	70.1 ± 53.1	99.7 ± 24.2	43.9 ± 6.1	65.4 ± 4.7
IL-12 (pg/mL)	76.9 ± 40.5	73.7 ± 19.5	40.1 ± 8.6	51.6 ± 0.6
IL-18 (pg/mL)	573.1 ± 58.8	234.3 ± 40.1	625.8 ± 251.9	359.3 ± 4.3
M30 (pg/mL)	6895.6 ± 353.2	6714.5 ± 233.4	6549.1 ± 698.5	6039.5 ± 287.9
TNFα (pg/mL)	61.7 ± 20.8	55.3 ± 3.2	60.6 ± 2.1	58.3 ± 3.2
**Control lungs**				
GM-CSF (pg/mL)	83.1 ± 12.6	77.1 ± 21.6	80.6 ± 18.7	47.8 ± 30.9
IFNγ (pg/mL)	393.1 ± 128.5	500.4 ±13.9	162.9 ± 134.4	0.0 ± 0.0
IL-1α (pg/mL)	140.9 ± 146.4	51.5 ± 3.6	56.4 ± 37.1	64.4 ± 2.1
IL-1β (pg/mL)	6142.5 ± 7626.7	4349.7 ± 4219.9	2109.8 ± 1847.6	1003.6 ± 1.9
IL-1rα (pg/mL)	1827.7 ± 2239.7	2207.5 ± 2090.4	1690.9 ± 1269.9	258.3 ± 18.1
IL-2 (pg/mL)	22.1 ± 9.6	31.9 ± 4.5	9.5 ± 8.5	0.0 ± 0.0
IL-4 (pg/mL)	29.8 ± 19.4	28.5 ± 21.9	12.1 ± 13.4	11.7 ± 5.1
IL-6 (pg/mL)	264.3 ± 285.9	350.9 ±118.9	305.1 ± 111.9	617.8 ± 20.3
IL-8 (pg/mL)	3113.5 ± 2780.7	2639.5 ± 1600.2	3100.1 ± 2244.9	2529.6 ± 77.3
IL-10 (pg/mL)	14.4 ± 9.2	46.9 ± 49.7	14.2 ± 11.6	7.9 ± 0.5
IL-12 (pg/mL)	20.4 ± 8.6	20.1 ± 15.1	14.6 ± 3.9	2.3 ± 3.2
IL-18 (pg/mL)	312.8 ± 239.3	178.8 ± 130.3	122.9 ± 68.2	52.2 ± 0.8
M30 (pg/mL)	1832.1 ± 744.3	1622.8 ± 819.5	2045.3 ± 367.8	2143.7 ± 242.7
TNFα (pg/mL)	32.5 ± 16.2	13.9 ± 13.1	19.4 ± 15.2	3.7 ± 5.3

Supplementary Table 9 | Inflammatory cytokines in BAL fluid collected from injured and control lungs. Analysis of inflammatory cytokines in BAL fluid throughout 36 hours of cross-circulation (*n* = 8). All values represent mean ± standard deviation.

Primary antibodies	Application	Product number	Dilution
Rabbit anti-aquaporin 5	IHC	ab78486	1:100
Rabbit anti-caspase 3	IHC	ab13847	1:100
Rabbit anti-CD31	IHC	ab28364	1:100
Rabbit anti-CD163	IHC	ab87099	1:100
Rabbit anti-connexin 43	IHC	ab11370	1:100
Rabbit anti-neutrophil elastase	IHC	ab68672	1:100
Rabbit anti-surfactant protein C	IHC	ab196677	1:100
Rabbit anti-ZO-1	IHC	sc-10804	1:100
Rabbit anti-ZO-3	IHC	ab205882	1:100
**Secondary antibodies**			
Goat Anti-Mouse IgG (519)	IHC	ab6785	1:200
Goat Anti-Mouse IgG (647)	IHC	ab150115	1:200
Goat Anti-Rabbit IgG (594)	IHC	ab150080	1:200

Supplementary Table 10 | Primary and secondary antibodies.

Marker	Application	Product number
D-Dimer	Blood	PD0130
Fibrinogen	Blood	PF0216
M30	BAL/Blood	E07C0762
Pepsin	BAL	LS-F20833
Plasma Free Hemoglobin	Blood	PF0230
P-Selectin	BAL/Blood	PP0118

Supplementary Table 11 | ELISA product kits.

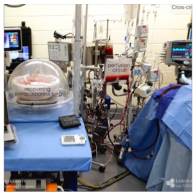

Supplementary Movie 1 | Interventional cross-circulation: experimental and procedural overview. Delivery of gastric contents and resulting *in-situ* lung injury, lung procurement and cannulation, isolated *ex-vivo* lung perfusion (EVLP) leading to initiation of interventional cross-circulation with the use of a recipient swine providing prolonged normothermic support and homeostasis, performance of therapeutic interventions including bronchoalveolar lavage, surfactant replacement, and alveolar recruitment, and subsequent recovery and regeneration of severely damaged lungs.

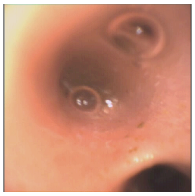

Supplementary Movie 2 | Delivery of gastric contents via video bronchoscopy. Delivery of standardized gastric contents into a single lung of living donor swine.

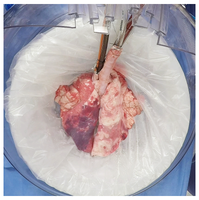

Supplementary Movie 3 | Reperfusion of severely damaged lungs. Injured lung (screen left), control lung (screen right).

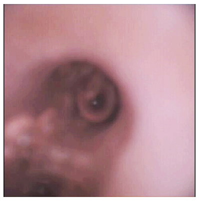

Supplementary Movie 4 | Airway lavage. Bronchoscopic delivery and subsequent removal of normal saline in segment of injured lung.

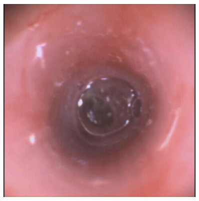

Supplementary Movie 5 | Surfactant replacement. Bronchoscopic delivery of surfactant following airway lavage.

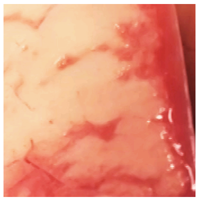

Supplementary Movie 6 | Alveolar recruitment. Close-up view of the lung as distal alveoli are recruited with ventilatory maneuvers.

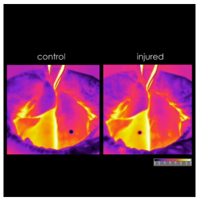

Supplementary Movie 7 | Hypothermic probe test. Video thermography of hypothermic probe test in injured and control lungs.

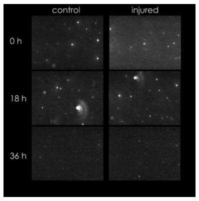

Supplementary Movie 8 | NanoSight exosome analysis. Exosomes isolated from BAL fluid of injured and control lungs at 0, 18, and 36 h of interventional cross-circulation.
