# Supplementary material for: Regeneration of severely damaged lungs using an interventional cross-circulation platform
Source: Nat Commun. 2019 May 7;10:1985. doi: 10.1038/s41467-019-09908-1 (PMC6504972; doi:10.1038/s41467-019-09908-1)
Supplement: Supplementary file 2 — Description of Additional Supplementary Files [file 41467_2019_9908_MOESM2_ESM.docx]

**Description of Additional Supplementary Files**

**File Name: Supplementary Movie 1**

**Description:** Interventional cross-circulation: experimental and procedural overview. Delivery of gastric contents and resulting in-situ lung injury, lung procurement and cannulation, isolated ex-vivo lung perfusion (EVLP) leading to initiation of interventional crosscirculation with the use of a recipient swine providing prolonged normothermic support and homeostasis, performance of therapeutic interventions including bronchoalveolar lavage, surfactant replacement, and alveolar recruitment, and subsequent recovery and regeneration of severely damaged lungs.

**File Name: Supplementary Movie 2**

**Description:** Delivery of gastric contents via video bronchoscopy. Delivery of standardized gastric contents into a single lung of living donor swine.

**File Name: Supplementary Movie 3**

**Description:** Reperfusion of severely damaged lungs. Injured lung (screen left), control lung (screen right).

**File Name: Supplementary Movie 4**

**Description:** Airway lavage. Bronchoscopic delivery and subsequent removal of normal saline in segment of injured lung.

**File Name: Supplementary Movie 5**

**Description:** Surfactant replacement. Bronchoscopic delivery of surfactant following airway lavage.

**File Name: Supplementary Movie 6**

**Description:** Alveolar recruitment. Close-up view of the lung as distal alveoli are recruited with ventilatory maneuvers.

**File Name: Supplementary Movie 7**

**Description:** Hypothermic probe test. Video thermography of hypothermic probe test in injured and control lungs.

**File Name: Supplementary Movie 8**

**Description:** NanoSight exosome analysis. Exosomes isolated from BAL fluid of injured and control lungs at 0, 18, and 36 h of interventional cross-circulation.
