# Supplementary material for: Regeneration of severely damaged lungs using an interventional cross-circulation platform
Source: Nat Commun. 2019 May 7;10:1985. doi: 10.1038/s41467-019-09908-1 (PMC6504972; doi:10.1038/s41467-019-09908-1)
Supplement: Supplementary file 11 — Reporting Summary [file 41467_2019_9908_MOESM11_ESM.pdf]

natureresearch

Corresponding author(s): Gordana Vunjak-Novakovic, Matthew Bacchetta

Last updated by author(s): Feb 11, 2019

Reporting Summary

Nature Research wishes to improve the reproducibility of the work that we publish. This form provides structure for consistency and transparency in reporting. For further information on Nature Research policies, see Authors & Referees and the Editorial Policy Checklist.

Statistics

For all statistical analyses, confirm that the following items are present in the figure legend, table legend, main text, or Methods section.

n/a	Confirmed	
☐	☒	The exact sample size (*n*) for each experimental group/condition, given as a discrete number and unit of measurement
☐	☒	A statement on whether measurements were taken from distinct samples or whether the same sample was measured repeatedly
☐	☒	The statistical test(s) used AND whether they are one- or two-sided<br>*Only common tests should be described solely by name; describe more complex techniques in the Methods section.*
☐	☒	A description of all covariates tested
☐	☒	A description of any assumptions or corrections, such as tests of normality and adjustment for multiple comparisons
☐	☒	A full description of the statistical parameters including central tendency (e.g. means) or other basic estimates (e.g. regression coefficient) AND variation (e.g. standard deviation) or associated estimates of uncertainty (e.g. confidence intervals)
☐	☒	For null hypothesis testing, the test statistic (e.g. *F*, *t*, *r*) with confidence intervals, effect sizes, degrees of freedom and *P* value noted<br>*Give P values as exact values whenever suitable.*
☒	☐	For Bayesian analysis, information on the choice of priors and Markov chain Monte Carlo settings
☒	☐	For hierarchical and complex designs, identification of the appropriate level for tests and full reporting of outcomes
☒	☐	Estimates of effect sizes (e.g. Cohen's *d*, Pearson's *r*), indicating how they were calculated

Our web collection on statistics for biologists contains articles on many of the points above.

Software and code

Policy information about availability of computer code

Data collection	ResearchIR v. 853 4.40.6 software (FLIR)
Data analysis	ImageJ v. 1.51m9<br>MATLAB v. R2016b (Mathworks)

For manuscripts utilizing custom algorithms or software that are central to the research but not yet described in published literature, software must be made available to editors/reviewers. We strongly encourage code deposition in a community repository (e.g. GitHub). See the Nature Research guidelines for submitting code & software for further information.

Data

Policy information about availability of data

All manuscripts must include a data availability statement. This statement should provide the following information, where applicable:

- Accession codes, unique identifiers, or web links for publicly available datasets
- A list of figures that have associated raw data
- A description of any restrictions on data availability

The authors declare that all data reported in the paper and Supplementary Information will be made available to other investigators if requested.

Field-specific reporting

Please select the one below that is the best fit for your research. If you are not sure, read the appropriate sections before making your selection.

☒ Life sciences ☐ Behavioural & social sciences ☐ Ecological, evolutionary & environmental sciences

For a reference copy of the document with all sections, see nature.com/documents/nr-reporting-summary-flat.pdf

Life sciences study design

All studies must disclose on these points even when the disclosure is negative.

Sample size	The study was designed as a proof-of-concept study (n = 8 animals) for the purposes of testing interventional procedures and demonstrating the ability to support and recover the function of acutely injured lungs with cross-circulation for 36 hours, during which standard therapeutic interventions could be performed and the functional recovery of lungs assessed. Our hypothesis was that cross-circulation could provide a sufficiently robust level of extracorporeal support to enable multiple therapeutic interventions and recovery of extracorporeal lung function. The study was conducted with the minimum number of animals needed to demonstrate reproducibility and achieve statistical significance between time points. Data from this initial study will be used to conduct power analysis for subsequent investigations.
Data exclusions	No data were excluded from analysis.
Replication	All samples and data from n = 8 experiments were processed and analyzed in parallel. As established experimental models and methods were used, all attempts at replication were successful.
Randomization	Randomization of sampling. Samples of extracorporeal lung collected for histologic, microscopic, and pathologic analyses were collected randomly during procedures according to a pre-determined lung map (Supplementary Fig. 4a) with 12 regions arbitrarily numbered for each lung.
Blinding	Blinded review. All analytical assessments were blinded to the maximum practical extent. Pathologic analysis was performed by an independent expert to eliminate bias.

Reporting for specific materials, systems and methods

We require information from authors about some types of materials, experimental systems and methods used in many studies. Here, indicate whether each material, system or method listed is relevant to your study. If you are not sure if a list item applies to your research, read the appropriate section before selecting a response.

Materials & experimental systems

n/a	Involved in the study	
☐	☒	Antibodies
☒	☐	Eukaryotic cell lines
☒	☐	Palaeontology
☐	☒	Animals and other organisms
☒	☐	Human research participants
☒	☐	Clinical data

Methods

n/a	Involved in the study	
☒	☐	ChIP-seq
☒	☐	Flow cytometry
☒	☐	MRI-based neuroimaging

Antibodies

Antibodies used	Please see Supplementary Table 10 for a complete list of all primary and secondary antibodies, and Supplementary Table 11 for a complete list of all ELISA product kits used in this study.<br><br>Primary antibodies: Rabbit, aquaporin 5 (Abcam, ab78486), Rabbit anti-caspase 3 (Abcam, ab13847), Rabbit anti-CD31 (Abcam, ab28364), Rabbit anti-CD163 (Abcam, ab87099), Rabbit anti-connexin 43 (Abcam, ab11370), Rabbit anti-neutrophil elastase (Abcam, ab68672), Rabbit anti-surfactant protein C (Abcam, ab196677), Rabbit anti-ZO-1 (Santa Cruz Biotechnologies, sc-10804), Rabbit anti-ZO-3 (Abcam, ab205882)<br><br>Secondary antibodies: Goat Anti-Mouse IgG 519 (ab6785), Goat Anti-Mouse IgG 647 (ab150115), Goat Anti-Rabbit IgG 594 (ab150080)
Validation	Validation statements of primary antibodies are available on the manufacturers' websites.

Animals and other organisms

Policy information about studies involving animals; ARRIVE guidelines recommended for reporting animal research

Laboratory animals	Species: Sus scrofa domesticus<br>Sex: male, female<br>Strain: Partially inbed swine (described in PMID: 137560)<br>Age: 5 – 8 months
Wild animals	This study did not involve wild animals.

Field-collected samples	This study did not involve samples collected from the field.
Ethics oversight	The study received approval from the Institutional Animal Care and Use Committee at Columbia University. Animal care and procedures were conducted in accordance with the US National Research Council of the National Academies Guide for the Care and Use of Laboratory Animals, Eighth Edition.

Note that full information on the approval of the study protocol must also be provided in the manuscript.
